# Anti-inflammatory and anti-oxidant properties of Melianodiol on DSS-induced ulcerative colitis in mice

**DOI:** 10.7717/peerj.14209

**Published:** 2022-10-25

**Authors:** Jinhuang Shen, Xinhua Ma, Yubin He, Yanjun Wang, Tianhua Zhong, Yonghong Zhang

**Affiliations:** 1 Fujian Medical University, Fuzhou, China; 2 Key Laboratory of Marine Biogenetic Resources, Third Institute of Oceanography, State Oceanic Administration, Xiamen, China

**Keywords:** Melianodiol, Triterpenoid, Ulcerative colitis, RAW264.7, Anti-inflammatory, Anti-oxidant

## Abstract

**Background:**

Ulcerative colitis is a unique inflammatory bowel disease with ulcerative lesions of the colonic mucosa. Melianodiol (MN), a triterpenoid, isolated from the fruits of the Chinese medicinal plant *Melia azedarach*, possesses significant anti-inflammatory properties.

**Objective:**

The present study investigated the protective effects of MN on lipopolysaccharide (LPS)-induced macrophages and DSS-mediated ulcerative colitis in mice.

**Methods:**

In the study, mice were given MN (50, 100, and 200 mg/kg) and 5-ASA (500 mg/kg) daily for 9 days after induction by DSS for 1 week. The progress of the disease was monitored daily by observation of changes in clinical signs and body weight.

**Results:**

The results showed that MN effectively improved the overproduction of inflammatory factors (IL-6, NO, and TNF-*α*) and suppressed the activation of the NF-*κ*B signalling cascade in LPS-mediated RAW264.7 cells. For DSS-mediated colitis in mice, MN can reduce weight loss and the disease activity index (DAI) score in UC mice, suppress colon shortening, and alleviate pathological colon injury. Moreover, MN treatment notably up regulated the levels of IL-10 and down regulated those of IL-1*β* and TNF-*α*, and inhibited the protein expression of p-JAK2, p-STAT3, iNOS, NF-*κ*B P65, p-P65, p-IKK*α/β*, and p-I*κ*B*α* in the colon. After MN treatment, the levels of MDA and NO in colonic tissue were remarkably decreased, whereas the levels of GSH, SOD, Nrf-2, Keap-1, HO-1, I*κ*B*α*, and eNOS protein expression levels were significantly increased.

**Conclusion:**

These results indicate that MN can activate the Nrf-2 signalling pathway and inhibit the JAK/STAT, iNOS/eNOS, and NF-*κ*B signalling cascades, enhance intestinal barrier function, and effectively reduce the LPS-mediated inflammatory response in mouse macrophages and DSS-induced intestinal injury in UC.

## Introduction

Ulcerative colitis (UC) is an inflammatory intestinal disease that mainly affects the colon (Høivik et al., 2013). The pathological features of UC include mucosal inflammation and ulcerative lesions (Yuan et al., 2019). The aetiology of UC remains unclear, which is associated with individual genetic susceptibility, immune dysfunction, damage to the intestine epithelium, and changes in gut microbiota (Yang et al., 2013). Persistent abdominal pain, blood in the stool, loss of appetite, and fatigue are the major symptoms of UC, which seriously affect the work and life of the patient. Patients with long-term recurrence are at an increased risk of reinfection and colon cancer (Ungaro et al., 2011). As the aetiology and pathogenesis are still unclear, the clinical treatment of UC is difficult. Although aminosalicylic acid, steroids, immunosuppressants, and biologics can relieve UC, they are prone to side effects such as toxicity, allergies, and upper gastrointestinal bleeding (Curro, Pugliese & Armuzzi, 2017). Therefore, there is an urgent requirement for more reliable and effective drugs.

In physiological responses to infection or damage, macrophages influence the progression of inflammatory processes (Alivernini et al., 2020). The production of proinflammatory mediators and aggravation of inflammation are inseparable from the action of macrophages; therefore, the course of UC is also inseparable from macrophages (Eissa et al., 2018). When activated, NF-*κ*B leads to the production of proinflammatory cytokines like TNF-*α*, IL-10, and IL-1*β*. Given the potential relevance of inflammation and macrophages, it is important to find a way to modulate the expression of inflammatory cytokines and control macrophage activation. Lipopolysaccharide (LPS) has been widely used to stimulate macrophages in inflammatory models in experiments on anti-inflammatory mechanisms. After LPS stimulation, the NF-*κ*B signalling cascade is activated, resulting in changes in the expression of related protein expression (Ren et al., 2020; Wang et al., 2021).

The NF-*κ*B signalling cascade and its induced expression of proinflammatory cytokines, oxidative stress, and apoptotic and antiapoptotic genes play vital roles in the pathogenesis of UC (Niu et al., 2015). NF-*κ*B is a heterodimeric protein complex comprising multiple subunits such as P65. It is inactive in the cytoplasmic complex with the inhibitory protein kappa B kinase alpha/beta (IKK*α/β*). Inhibitory protein kappa B (I*κ*B) kinase is activated by multiple extracellular stimuli to integrin receptors, catalysing the phosphorylation and ubiquitination of I*κ*B*α*, dissociation from NF-*κ*B, and final proteolytic degradation. This results in phosphorylation and translocation of NF-*κ*B P65 into the nucleus. In the nucleus, P65 transcription factors bind specific DNA sequences and modulate gene transcriptions (Sun et al., 2015).

It has been found that nuclear factor erythrocyte 2 associated factor 2 (Nrf-2) affects the transcription of the gene that regulates cellular anti-inflammatory and antioxidant mechanisms (Li et al., 2008). Interactions of Nrf-2 and NF-*κ*B have been proposed. Nrf-2/MEF studies have shown that phosphorylation induces I*κ*B*α* degradation and ubiquitination enhances IKK*β* activity (Thimmulappa et al., 2006). Increased oxidative stress due to Nrf-2 deficiency leads to increased expression of NF-*κ*B-mediated cytokines, as NF-*κ*B is readily activated under oxidative conditions (Yerra et al., 2013). Furthermore, Nrf-2 and NF-*κ*B have been shown to compete with the transcriptional coactivator, CBP (Sun, Chin & Zhang, 2009). Downregulation of NF-*κ*B P65 promotes Nrf-2-CBP binding (Liu, Qu & Shen, 2008). Therefore, regulating the crosstalk of Nrf-2 and the NF-*κ*B pathway may be an effective method for UC treatment.

Maliaceae plants are known for their highly diverse limonoid and triterpenoid structures with a broad range of bioactivities (Tundis, Loizzo & Menichini, 2014; Fang, Di & Hao, 2011; Zhao et al., 2010). *Melia azedarach* L. (chinaberry tree) belongs to the Meliaceae family. It is native to China, India, and Japan, and is widely cultivated in southern China and Southeast Asia. Its bark and fruit have been used for pesticides, pain relief, and skin diseases (Carpinella et al., 2006). Regardless, most studies have focused on the biological activities of the crude extracts of *M. azedarach*. The specific effects of a single biologically active constituent have, however, not yet been defined.

Previous studies have suggested that triterpenoids are suitable candidates as novel anti-inflammatory drugs. Pre-treatment of LPS-stimulated microglia with triterpenoids significantly blocked ROS formation and prevented the increase in NO, TNF-*α*, and IL-6 levels. Triterpenoid pre-treatment also significantly reduces iNOS and COX-2 expression (Lu et al., 2020). Additionally, the intervention of the NF-*κ*B signalling cascade alters the expression of genes involved in inflammation and apoptosis (Costa et al., 2012). Triterpenoids are widely distributed in the plant kingdom and represent a large class of compounds with highly interesting biological effects, including anti-inflammatory effects. Triterpenoids can effectively reduce the inflammatory response, decrease the expression of cytokines and inflammatory related protein pathway proteins, and reduce the adverse effects of inflammatory proteins on the body (Fernandez-Arche et al., 2010). Ginsenosides are triterpenoids (Chen, 2020). Ginsenoside Rf, which is only present in ginseng, has been reported to be critical for the regulation of lipid metabolism (Liang et al., 2019), neuroprotective (Chen et al., 2021; Ye et al., 2016), analgesic and anti-allergic activities (Liu et al., 2019). Anti-inflammatory activities of ginsenoside Rf have been reported in the context of inflammatory bowel disease using different cell types, including intestinal epithelial cells (HT-29) and mouse macrophages (RAW264.7) (Im, 2020). In particular, ginsenoside Rf inhibited inflammatory cytokine production by downregulating p38-MAPK-, JNK-, and ERK-mediated NF-κB signalling pathways in HT-29 cells and RAW264.7 cells.

Melianodiol (MN), a monomeric compound isolated from *M. azedarach*, is a tertiarytriterpenoid. MN has shown anti-inflammatory (Pan et al., 2014), anti-radical, cytotoxic (Kurimoto et al., 2014), and antibacterial activities (Biavatti et al., 2002) in previous studies. In this study, we comprehensively explained the anti-inflammatory activities and underlying mechanism of MN in LPS-induced macrophages and DSS-stimulated UC mouse models. For *in vitro* experiments, the level of pro-inflammatory cytokine and associated protein expression levels in JAK/STAT, iNOS/eNOS and NF-*κ*B signalling cascade was measured in RAW264.7 cells mediated by LPS. We also evaluated the therapeutic effects of MN on DSS-mediated UC in mice. Furthermore, the anti-ulcer mechanisms of MN were investigated through mucosal protection and modulation of the JAK/STAT, iNOS/eNOS, NF-*κ*B, and Nrf-2 signalling cascades.

## Materials and Methods

### Chemicals, reagents and antibodies

**Figure 1:**
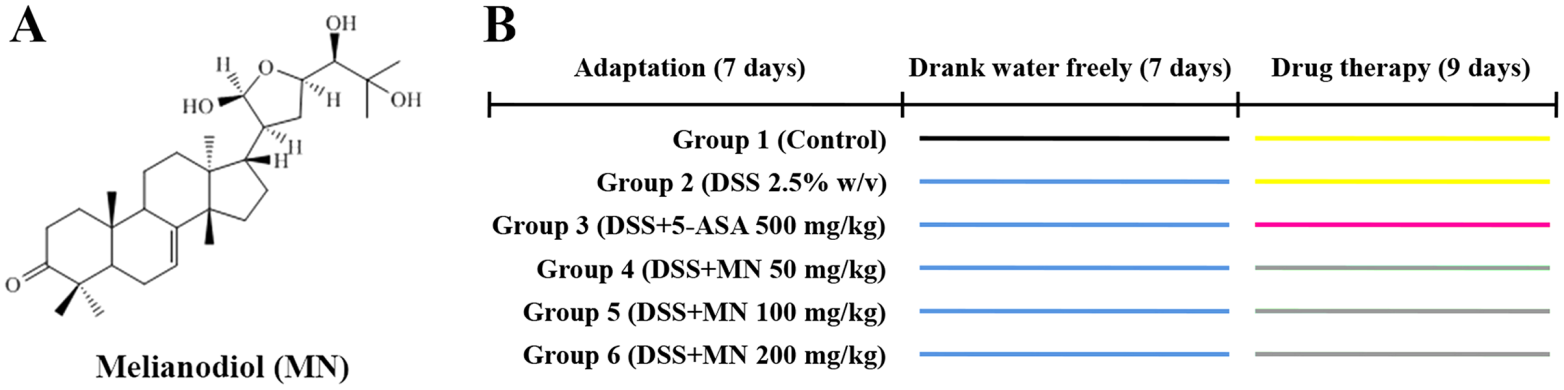
**(A) The structure of MN (Melianodiol) drawn by ChemDraw Professional 15.0 of ACS Document 1996. **(B) The process of experimental model of DSS-induced colitis in mice (Black color: Control mice received distilled water for 7 days; Blue color: Mice received DSS (2.5%) w/v in distilled water for 7 days; Grey color: Mice received DSS (2.5%) in distilled water and MN (50, 100, and 200 mg/kg) orally for 7 days, and only MN (50, 100, and 200 mg/kg) orally for another 2 days; Pink color: Mice received DSS (2.5%) in distilled water and 5-ASA (500 mg/kg) orally for 7 days, and only 5-ASA (500 mg/kg) orally for another 2 days; Yellow color: Mice received CMC-Na 0.5% for 2 days.

Melianodiol (MN) was isolated from the fruits of *M. azedarach* by our research group. The structure of MN is shown in Fig. 1A (Xu et al., 2008). NO, IL-6, IL-10, and TNF-*α* ELISA kits were provided by Shanghai MLBIO Biotechnology Co. Ltd. (Shanghai, China). LPS was obtained from Sigma (Sigma Aldrich, St. Louis, MO, USA). 5-Aminosalicylic acid was provided by Shanghai Aladdin Biochemical Co. Ltd. (Shanghai, China). DSS was provided by MP Biomedicals (USA) and indomethacin by Aladdin (USA). Antibodies against *β*-actin, JAK2, STAT3, IKK*α/β*, NF-*κ*BP65, IKB*α*, p-IKK*α*/*β*, p-I*κ*B*α*, p-P65, iNOS, eNOS, Nrf-2, HO-1, and Keap1 were provided by Cell Signaling Technology (Danvers, MA, USA). SOD, NO, GSH, and malondialdehyde (MDA) were obtained from the Nanjing Jian-Cheng Bioengineering Institute (Jiangsu, China).

### Cell culture

RAW264.7 cells obtained from the China Center for Cultivated Studies (Shanghai, China) were maintained in DMEM containing 1% penicillin and streptomycin, 10% FBS, and under 5% CO_2_ at 37 °C. The cells were stimulated by LPS. Briefly, the cells were seeded in a 96-well plate (1 × 10^5^ cells/well). After 8 h of pre-incubation under 5% CO_2_ at 37 °C, LPS (2 µg/mL) was added after compounds for 12 h. The cells or supernatant of the cell culture were collected 24 h later (Gasparotto et al., 2013).

### Determination of cytokine secretion levels of RAW.264.7 cells

To determine NO, IL-10, TNF-*α*, and IL-6 production, RAW264.7 cells were pretreated with MN for 1 h and then stimulated with 1 μg/mL of LPS in the presence of the MN for 48 h. The levels of NO, IL-10, TNF-*α*, and IL-6 were determined using ELISA based on the manufacturer’s instruction. The absorbance was measured at 450 nm by a microplate reader.

### Animals

Male C57BL/6 mice (6 weeks old, 18 ± 2 g), specific pathogen free, were purchased from the Laboratory Animal Center of Hangzhou Medical College, Zhejiang, China (Animal Production License Number: SCXK (Zhe) 2019-0002). All animals were adapted to the environment for 3 days before the experiment and fed and drank *ad libitum* at 22 ± 2 °C, 50 ± 5% relative humidity, and light dark cycle for 12 h. Mice were raised in SPF barrier environment. The animal experiment and welfare were in strict accordance with the relevant Guidelines, and the Experimental Animal Ethical and Welfare Committee of Fujian Medical University approved the study (IACUC FJMU 2017-0102).

### DSS-mediated colitis and drug therapy design

Sixty (60) mice were divided into six groups of 10 each: control, model (2.5% DSS), 5-ASA (500 mg/kg), MN low-dose (50 mg/kg), MN medium-dose (100 mg/kg), and MN high-dose (200 mg/kg) groups. Except for control mice, 2.5% DSS was given to the mice in drinking water for 7 days to induce UC (Fig. 1B). For MN and 5-ASA groups, MN and 5-ASA were administrated orally once a day at 0.1 mL/10 g body weight. The DSS and normal groups received the same amount of 0.5% carboxymethylcellulose (CMC) sodium solution.

### Disease activity index (DAI)

The mice in each group were weighed, faecal characteristics were observed, and the faecal occult blood test was performed daily. DAI was evaluated based on a previously described method (Jia et al., 2021): 0 points for normal stool and negative occult blood test, 0% weight loss; one point for weak positive detection of occult blood, 1–5% weight loss; two points for a soft stool and occult blood test positive, 6–9% weight loss; three points for a soft stool, 10–15% weight loss; occult blood test strongly positive; four points for diarrhoea, gross bleeding, >15% weight loss.

### Haematoxylin & eosin (H & E) staining

After 9 days of administration, the mice were anaesthetised with an intraperitoneal injection of 5% pentobarbital (0.2 mL/10 g). Orbital blood samples were also obtained. The mice were sacrificed by cervical dislocation. Colonic tissue from the anus to the ileocecal area was collected and cut into three sections. One part was fixed immediately with 4% neutral formalin, decalcified, and embedded in paraffin wax. Colon tissues were observed using haematoxylin and eosin (H & E) staining for morphological examination. The other was stored at −80 °C for cytokine analysis, and the remaining intestinal tissue was frozen for western blotting assays.

### Immunofluorescence staining

Paraffin-embedded colonic tissue was sectioned into a 3-μm thick slice. The section was de-paraffinised with xylene, rehydrated in a gradient alcohol solution, and subjected to antigen retrieval. The colon section was washed with PBS five times for 3 min each and blocked with 5% albumin from BSA for 0.5 h at 22–26 °C. The tissue was incubated with primary antibody (NF-*κ*B, 1:400 dilution) for 10 h at 4 °C. After being washed with PBS solution 5 min and four times, the sections were incubated with fluorescent secondary antibody (1:500) for 50 min at 37 °C avoiding light. After washing with PBS solution, the sections were incubated with DAPI solution for 10 min to stain the cell nuclei. After passage with anti-fading medium, a fluorescence microscope was used for microscopic examination and image collection.

### Assessment of oxidative and cytokine damage levels *in vivo*

For the analysis of MDA, GSH, SOD, NO, TNF-*α*, IL-1*β*, and IL-10 in DSS-mediated colon tissue, the colon tissue samples were suspended in lysis buffer and ground using a homogeniser. The supernatant was collected *via* centrifugation (3,000 rpm, 10 min). The contents of MDA, GSH, SOD, and NO were determined using the corresponding kits. The IL-1*β*, IL-10, and TNF-*α* levels in the supernatant were determined by ELISA using mouse kits based on the manufacturer’s instructions.

### Western blot analysis

The effects of MN on the protein levels of IKK/*β*, NF-*κ*B P65, IKB*α*, p-IKK*α*/*β*, p-I*κ*B*α*, p-P65, JAK2, STAT3, iNOS, eNOS, Nrf-2, HO-1, and Keap1 in RAW 264.7 cells or UC mouse intestinal tissue were investigated by western blotting (Li et al., 2011). For the extraction of total protein *in vitro*, RAW 264.7 cells were plated in 6-well plates at a concentration of 6 × 10^5^ cells and cultured at 37 °C with or without MN for 12 h, then with LPS (2 μg/mL) for 24 h. After treatment, the cells were collected and lysed with lytic buffer. The total protein content was extracted from the mouse colon, and 30 mg of colon tissue from each group was ground with lytic buffer to obtain the total protein solution of the mouse colon. The total protein solution was mixed with bromophenol blue and denatured in boiling water for 10 min. The *in vitro* and *in vivo* protein solutions were separated using 10% SDS PAGE and passed to a PVDF membrane. Then, the membrane was incubated at 4 °C for 12 h with a 1:1,000 diluted primary antibody to specifically identify *β*-actin, IKK*α*/*β*, NF-*κ*B P65, I*κ*B*α*, p-IKK*α*/*β*, p-I*κ*B*α*, p-P65, JAK2, STAT3, iNOS, endothelial NOS (eNOS), Nrf-2, HO-1, and Keap1. After washing with TBST buffer, the membrane was incubated with HRP-linked secondary antibodies at 37 °C for 1 h. Antibody-specific proteins on the PVDF membrane were prepared using an enhanced chemiluminescence kit. The level of IKK/*β*, NF-*κ*BP65, I*κ*B*α*, p-IKK*α*/*β*, p-I*κ*B*α*, p-P65, JAK2, STAT3, iNOS, eNOS, Nrf-2, HO-1, and Keap1 in RAW264.7 cells and UC mouse intestinal tissues were measured using *β*-actin as a loading control.

### Data and statistical analysis

The measurement data were expressed as an average ± SD. SPSS version 16.0 was used for statistical analyses. One-way analysis of variance was performed to compare statistically significant differences among groups. Statistical significance was set at *p < 0.05, and high statistical significance at **p < 0.01.

## Results

### MN alleviated DSS-mediated UC symptoms

**Figure 2:**
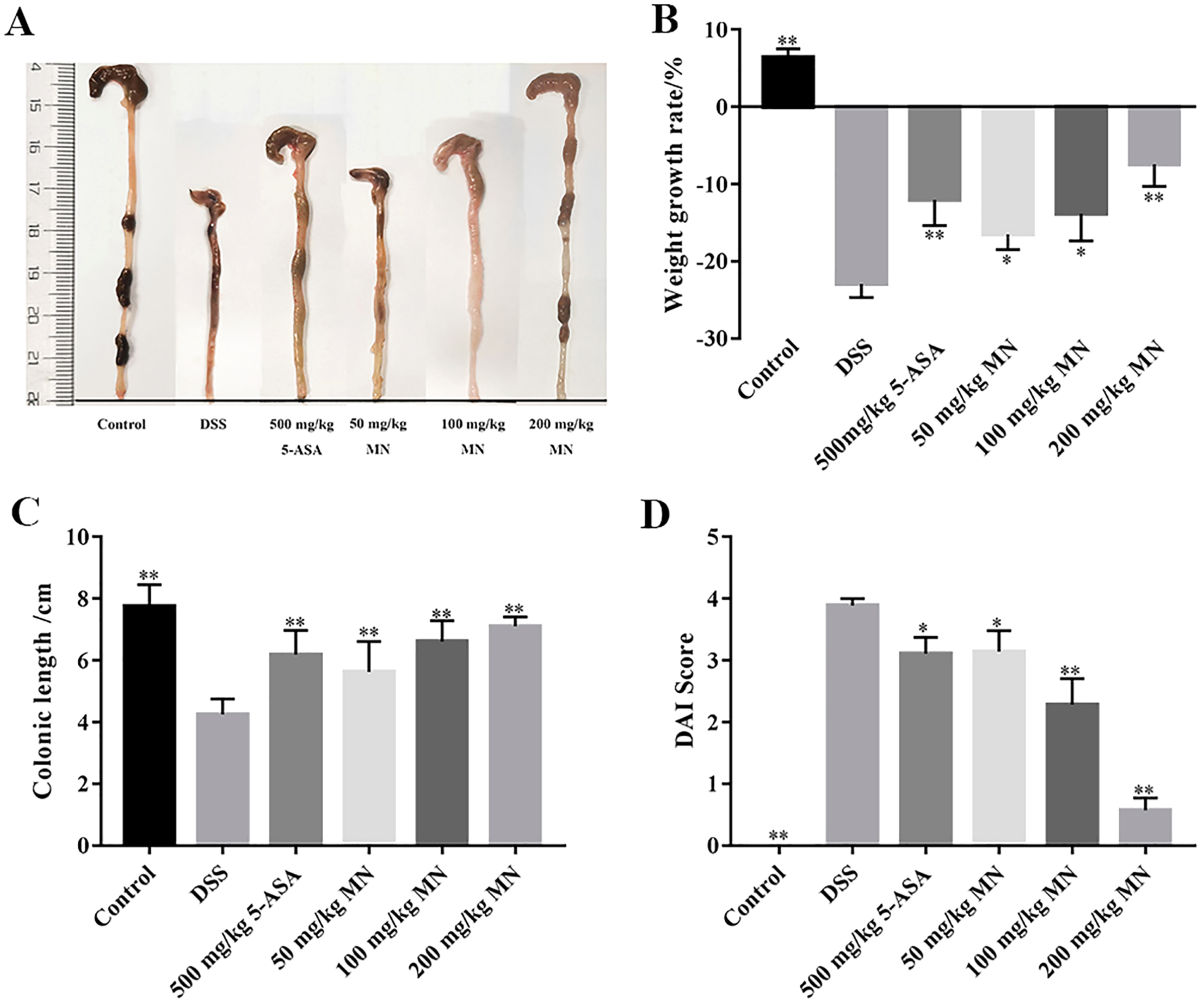
**Protective effects** **of MN against DSS-induced UC in mice.** (A) Colon macroscopic damage and length. (B) MN treatment (50, 100, and 200 mg/kg) effectively decreased UC related weight loss on day 9. (C) MN treatment resulted in significantly longer colonic length compared with the DSS-only models. (D) MN (50, 100, and 200 mg/kg) significantly reduced DAI score in mice. **p < 0.01, *p < 0.05 compared with the DSS group. Mean values ± SEM are shown. *n* = 10. (5-ASA (5-Aminosalicylic acid), as positive control).

In this experiment, a DSS-mediated inflammation model in mice was established, and the effect of MN on colitis was studied. 5-Aminosalicylic acid (5-ASA) is an effective drug for UC treatment. In contrast to the control group, which showed steady weight gain and soft stools, the UC group showed significant changes on day 5. The weight loss of the mice in the MN and 5-ASA groups was not as significant as that in the UC group (Fig. 2B). Diarrhoea and blood in the stool occurred on days 3–8 in the 2.5% DSS-treated groups (UC, MN, and 5-ASA groups). The difference was that diarrhoea and blood in the stool in the MN and 5-ASA groups were lighter than those in the UC group. The DAI was notably higher in the UC group than in the control group, while the DAI was lower in the MN and 5-ASA groups (Fig. 2D). As shown in Figs. 2A and 2C, the colonic length of the UC group was notably shortened, and there was obvious congestion, oedema, and atrophy compared to the control group. These symptoms were much milder in the MN and 5-ASA groups. These results showed that MN notably improved UC symptoms in mice, including weight loss, diarrhoea, blood in the stool, and oedema.

### MN reduced the secretion levels of cytokines in RAW264.7 cells

**Figure 3:**
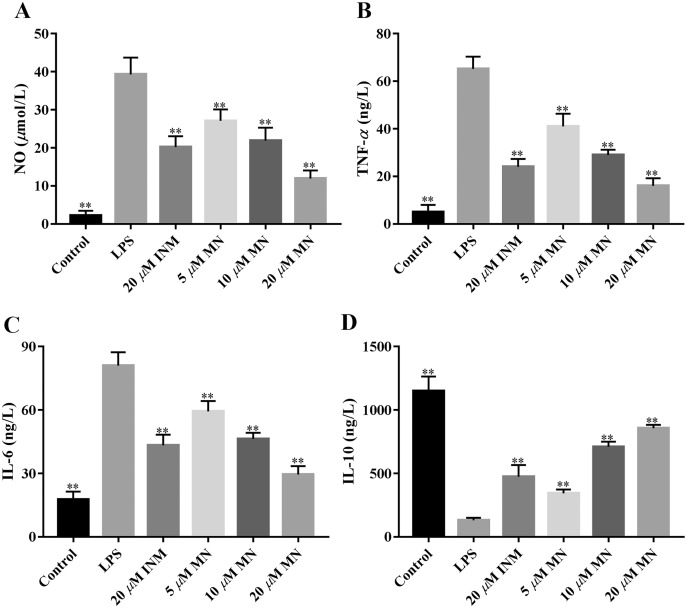
**Effects of MN on cytokines in RAW264.7 cells.** Treatment with MN down-regulated colon tissue NO (A), TNF-*α* (B), IL-6 (C), and up-regulated IL-10 (D) levels in RAW264.7 cells. Graphs represent Mean values ± SEM. **p < 0.01 compared with the LPS group. *n* = 5. (INM (Indomethacin), as positive control).

NO, IL-6, IL-10, and TNF-*α* are frequently used as indicators of the anti-inflammatory effect of compounds (Huang et al., 2021). We determined the secretion of NO, IL-6, TNF-*α*, and IL-10 by macrophages treated with MN. As shown in Figs. 3A–3C, the secretion of NO, TNF-*α* and IL-6 in the LPS group was notably increased compared to that in the control group. MN treatment notably reduced the secretion of TNF-*α*, IL-6 and NO in macrophages. As shown in Fig. 3D, after 24 h of LPS treatment, the level of IL-10 in the LPS group exhibited a notable decrease compared to the control group. After adding MN, the level of IL-10 in RAW264.7 cells was notably improved. The above experimental result indicated that MN pre-treatment could effectively reduce the secretion of NO, IL-6, and TNF-*α* and increase the secretion of IL-10 in a dose-dependent manner, showing good LPS-induced inflammation mitigation effects in macrophages. Indomethacin (INM), a non-steroidal drug with anti-inflammatory and analgesic properties, was used as the positive control.

### MN improved oxidative and inflammatory damage in UC mice

**Figure 4:**
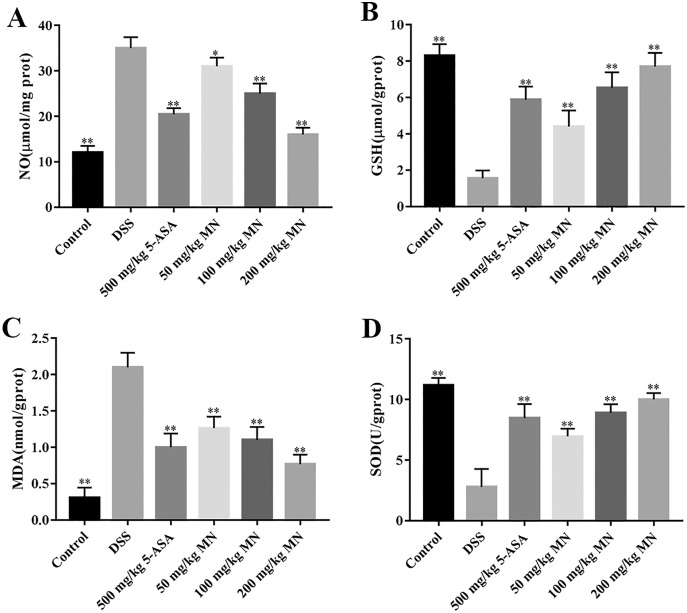
**Antioxidant effect of MN on DSS-induced UC.** Levels of NO (A), GSH (B), MDA (C), and SOD activity (D) in colon homogenate. Graphs represent Mean values ± SEM. *p < 0.05, **p < 0.01 compared with the DSS group. *n* = 10.

**Figure 5:**
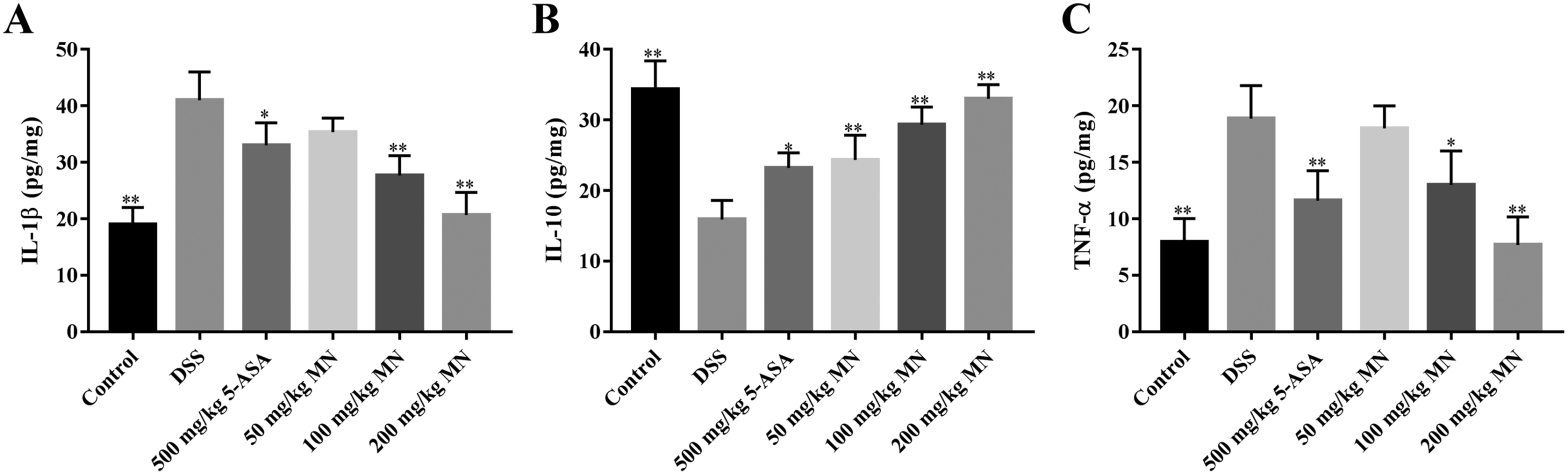
**Effects of MN on colon tissue cytokines in UC mice.** Treatment with MN down-regulated colon tissue IL-1*β* (A), up-regulated IL-10 (B), and TNF-*α* (C) levels in UC mice. Graphs represent Mean values ± SEM. *p < 0.05, **p < 0.01 compared with the DSS group. *n* = 10.

The intestines of mice with colitis were collected to measure the degree of oxidative stress. From the results in Figs. 4A and 4B, DSS induced a notable increase in colonic NO and MDA contents compared with the control group. In addition, the levels of NO and MDA in MN (100 and 200 mg/kg)-treated mice were notably decreased compared with the DSS group. As shown in Figs. 4C and 4D, GSH and SOD levels in the DSS-induced mice were notably reduced, while the levels of GSH and SOD were notably improved after MN treatment compared to the DSS group. Intestinal inflammatory factors were also included in the experiment, as shown in Figs. 5A–5C. Compared to the control group, DSS promoted the secretion of pro-inflammatory factors (TNF-*α* and IL-1*β*) and decreased the secretion of IL-10, while the MN group reversed this phenomenon. The levels of TNF-*α* and IL-1*β* in the intestinal tract of the MN group showed a notable downward trend, whereas IL-10 showed a significant upward trend. Generally, MN is beneficial for improving DSS-induced intestinal pressure and inflammatory response in mice.

### Inhibitory effect of MN on the NF- κB signalling cascade in UC mice and RAW264.7 cells

**Figure 6:**
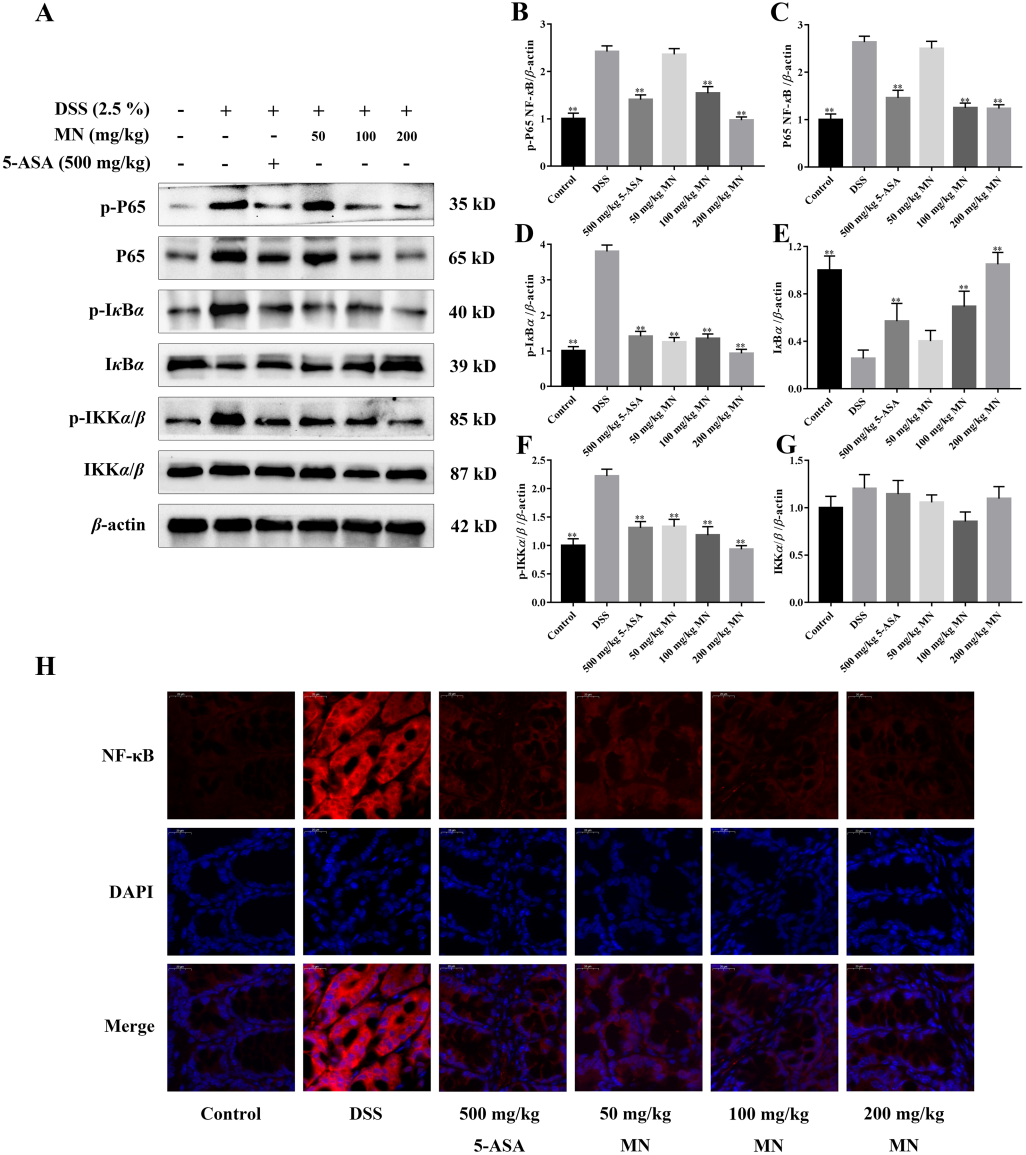
**Inhibitory effect of MN on NF-*κ*B signaling pathway in UC mice.** (A) Western blot analysis of protein expression levels of NF-*κ*B P65, p-IKK*α*/*β*, and p-I*κ*B*α* in colon tissue. (B) Immunofluorescence analysis of NF-*κ*B P65 (red) in colon mucosa (600 × magnification). DAPI was used for nuclear staining (blue). Data are expressed as the mean values ± SEM, *n* = 10 per group. **p < 0.01 compared with the DSS group.

**Figure 7:**
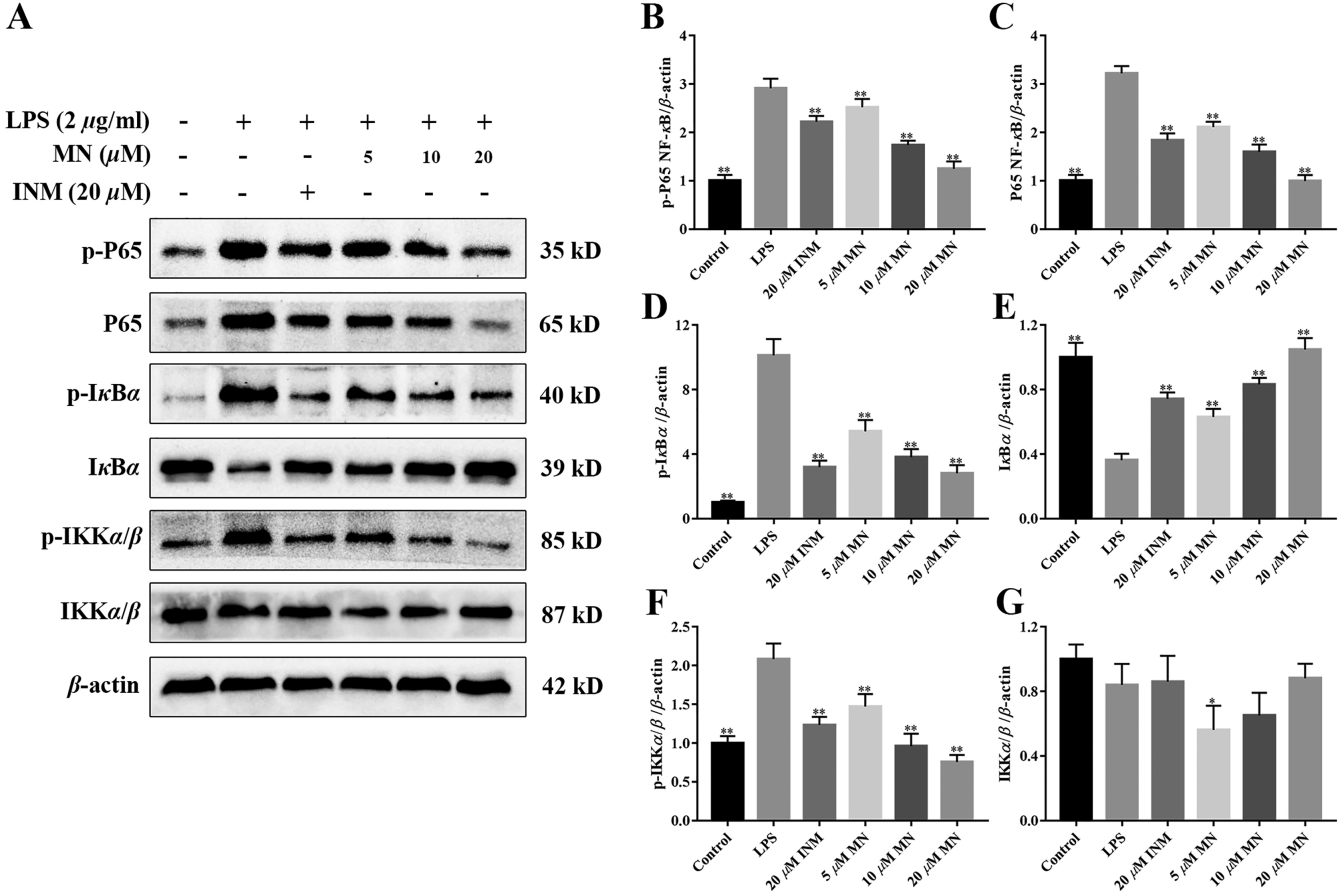
**Inhibitory effect of MN on NF-*κ*B signaling pathway in LPS-induced RAW264.7 cells.** Western blot analysis of protein expression levels of NF-*κ*B P65, p-P65, IKK*α*/*β*, p-IKK*α*/*β*, I*κ*B*α*, and p-I*κ*B*α* in cell homogenate. Data are expressed as the mean values ± SEM, *n* = 5 per group. *p < 0.05, **p < 0.01 compared with the LPS group.

The expression of NF-*κ*B P65, I*κ*B*α*, IKK*α*/*β*, p-P65, p-I*κ*B*α*, and p-IKK*α*/*β* was determined by western blotting to investigate the anti-inflammatory mechanisms of MN in colon tissues of the model group and LPS-mediated RAW264.7 cells (Staal, Bekaert & Beyaert, 2011; Fallahi-Sichani, Kirschner & Linderman, 2012). The results showed that MN downregulated the expression of NF-*κ*BP65 and p-P65 (Figs. 6A–6C and 7A–7C). Moreover, MN suppressed the phosphorylation and degradation of p-I*κ*B*α* (Figs. 6A, 6D, 6E, 7A, 7D and 7E) and p-IKK*α*/*β* (Figs. 6A, 6F, 6G, 7A, 7F and 7G). These data suggest that MN can block UC in mice and induce LPS-stimulated activation of the NF-*κ*B signalling cascade. NF-*κ*BP65 protein immunofluorescence analysis yielded similar results (Fig. 6H). This indicates that MN exhibits an inhibitory activity on the NF-*κ*B P65 pathway in UC mice.

### Activation effects of MN on the Nrf-2 signalling cascade in UC mice

**Figure 8:**
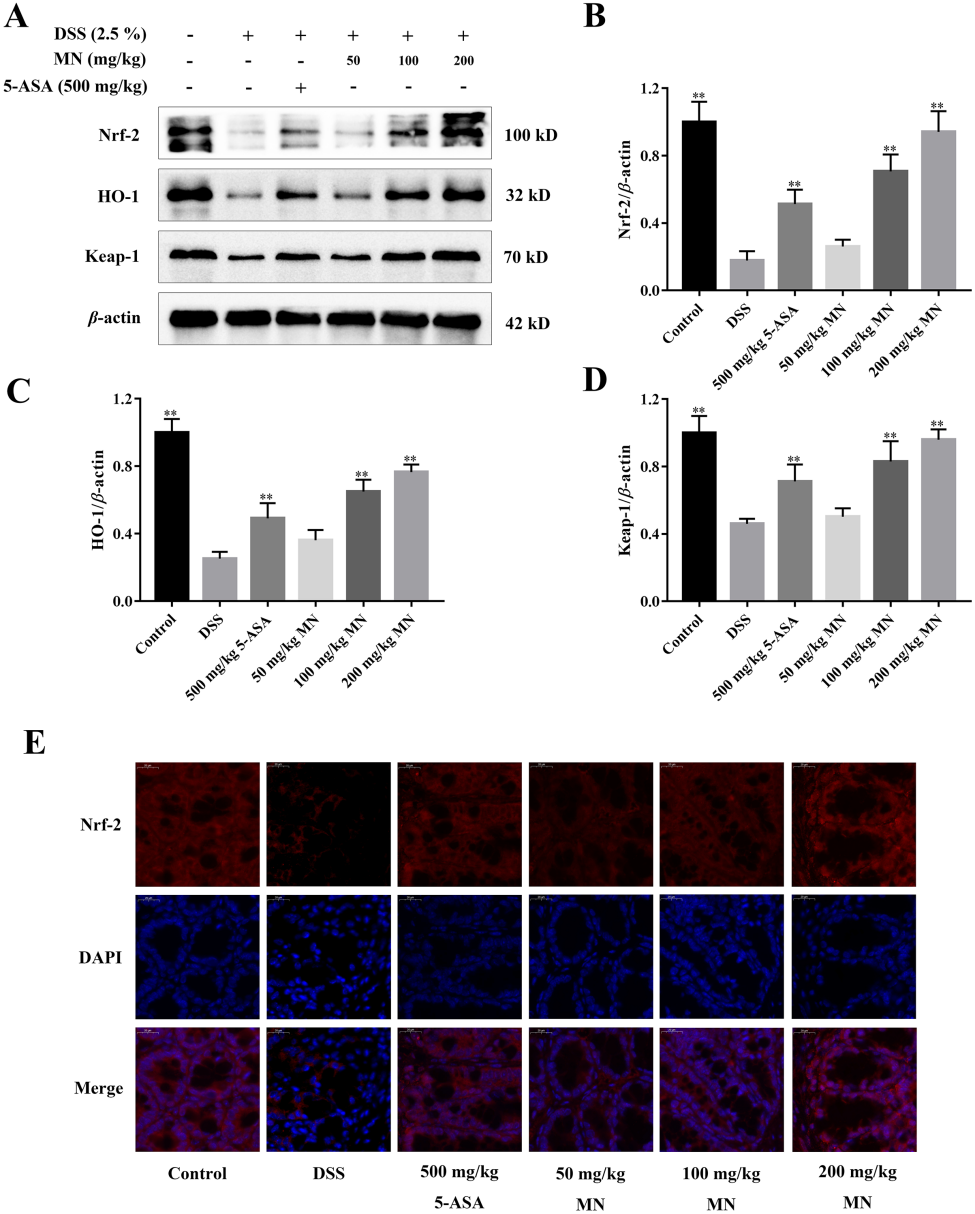
**The regulation effect of MN on Nrf-2 signaling pathway.** (A) MN therapy up-regulates the expression of key proteins Nrf-2, HO-1 and Keap-1 involved in the Nrf-2 signaling pathway in colon. (B) Immunofluorescence analysis of Nrf-2 (red) in colon mucosa (600 × magnification). DAPI was used for nuclear staining (blue). Data are expressed as the mean values ± SEM, *n* = 10 per group. **p < 0.01 compared with the DSS group. *n* = 10.

As shown in Figs. 8A–8D, our result indicated that compared to the control group, the levels of Nrf-2, HO-1, and Keap1 in the colon tissues of the model group were notably decreased, indicating that the antioxidant signalling cascade of the model group was notably suppressed. This is coherent with the result of the antioxidant parameter experiment (Figs. 3A–3D). MN intervention notably upregulated Nrf-2, HO-1, and Keap1 protein levels in the mouse colon (Figs. 8A–8D), indicating that MN intervention can notably activate the Nrf-2 signalling cascade. Nrf-2 protein immunofluorescence analysis showed similar results (Fig. 8E). This indicates that MN has an increased effect on the Nrf-2 pathway in UC mice.

### MN regulated the JAK2/STAT3 signalling cascade in UC mice

**Figure 9:**
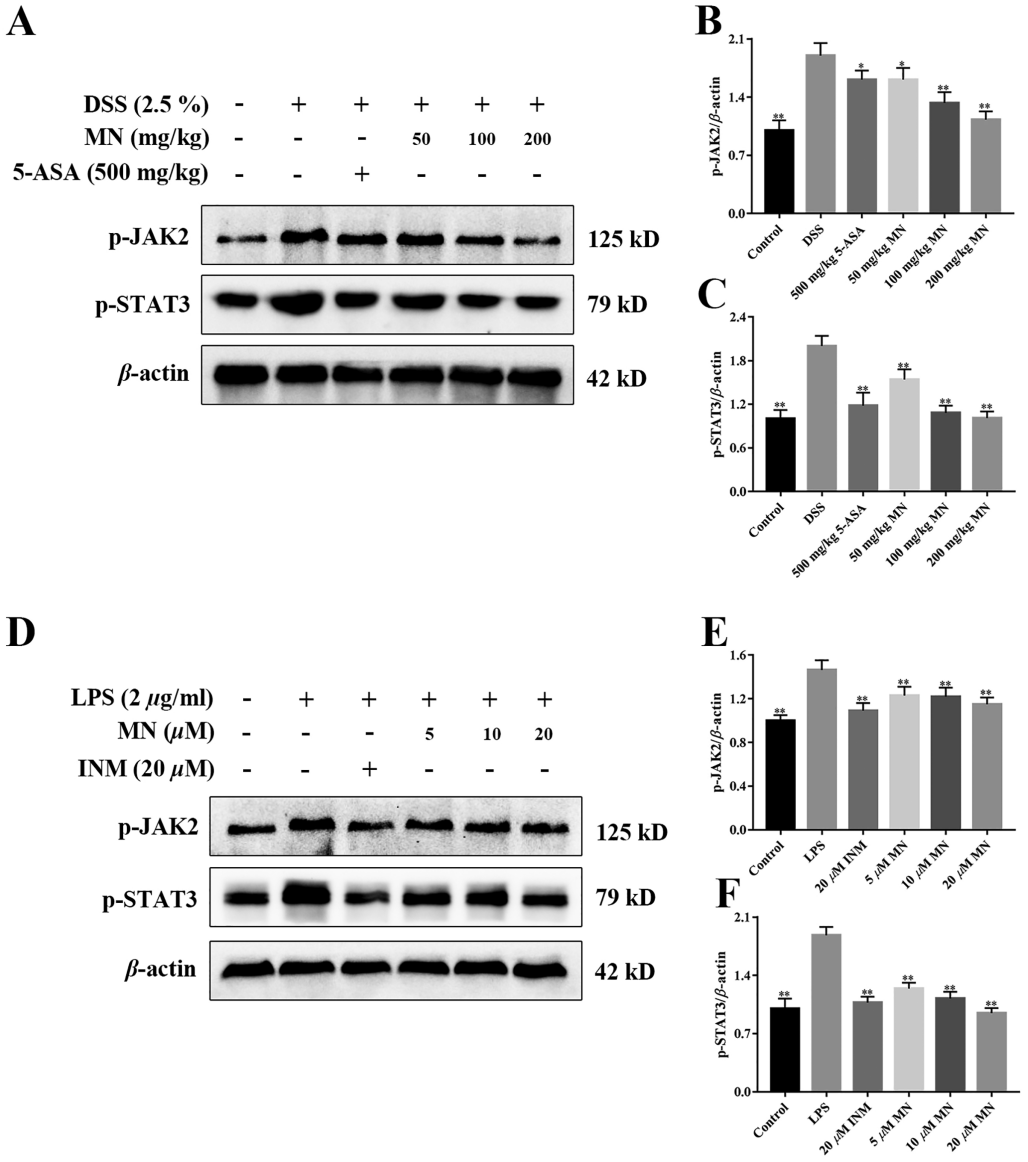
**Effect of MN on the protein expression of p-JAK2 and p-STAT3 in colon tissue and RAW264.7 cells.** (A) Western blotting of p-JAK2 and p-STAT3 protein and relative expression of p-JAK2 and p-STAT3 in colon. *n* = 10. (B) Relative expression of p-JAK2 and p-STAT3 in RAW264.7 cells. *n* = 5. Data are expressed as the mean values ± SEM. *p < 0.05, **p < 0.01 compared with the DSS or LPS group.

To investigate the effect of MN on the JAK2 and STAT3 signalling pathways in mice with colitis and macrophages, the levels of p-JAK2 and p-STAT3 were determined (Xiong et al., 2020). As shown in Figs. 9A–9F, compared with the control group, the expression levels of p-JAK2 and p-STAT3 in the intestine of UC mice (Figs. 9A–9C) and RAW264.7 cells (Figs. 9D–9F) increased notably, while the expression in the 5-ASA and MN groups decreased notably.

### MN regulated the iNOS/eNOS signalling cascade in UC mice

**Figure 10:**
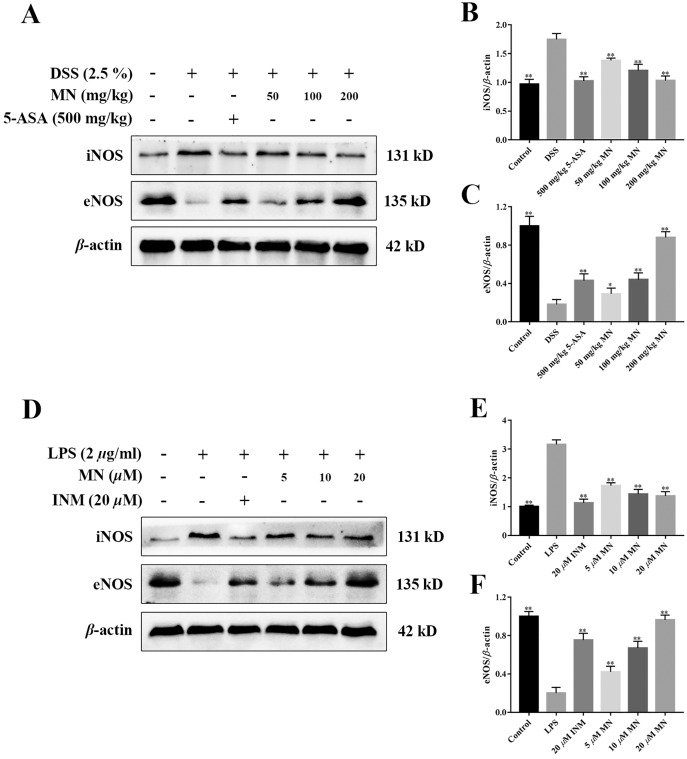
**Effect of MN 1 on the protein expression of iNOS and eNOS in colon and RAW264.7 cells.** (A) Western blotting of iNOS and eNOS protein and relative expression of iNOS and eNOS in colon. *n* = 10. (B) Relative expression of iNOS and eNOS in RAW264.7 cells. *n* = 5. Data are expressed as the mean values ± SEM. *p < 0.05, **p < 0.01 compared with the DSS or LPS group.

To study the effect of MN on the iNOS and eNOS signalling cascade in mice with colitis and RAW264.7 cells, the levels of iNOS and eNOS were measured. As shown in Figs. 10A–10F, compared to the control group, the high dose of MN and 5-ASA groups in the intestinal tract of UC mice (Figs. 10A, 10C) and MN and INM groups in RAW264.7 cells (Figs. 10D, 10F) increased eNOS protein expression, suggesting that the eNOS pathway was upregulated, while iNOS protein expression in the MN group (Figs. 10A, 10B, 9D and 9E) was downregulated in a dose-dependent manner.

### MN suppressed DSS-induced colitis development

**Figure 11:**
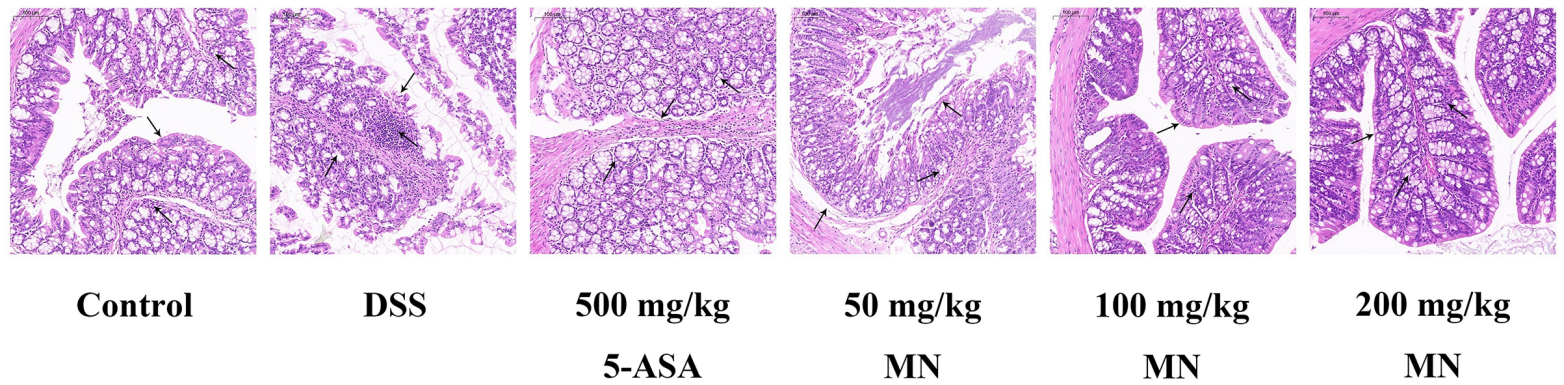
**Protective effect of MN against DSS-induced UC in mice.** Image of H & E staining obtained at 100× magnification in the colon.

Expectedly, compared with the control group, DSS stimulated the colon of mice to shrink notably due to inflammation and tissue injury. It is worth noting that compared with the DSS group, the MN group showed significantly less colonic contraction, indicating that MN inhibited bowel inflammation and tissue injury. To further support our data, we used H & E staining to evaluate tissue damage in DSS-induced colon tissue sections (Fig. 11). Expectedly, the DSS treatment group showed broad tissue injury, cell necrosis, and crypt loss compared to the control group. It is worth noting that the MN and 5-ASA groups showed reduced crypt loss, whereas the DSS group showed extensive areas of inflammation, causing tissue damage and inflammatory cell infiltration (Fig. 11). This finding, combined with previous observations, shows that MN exhibits effective results, indicating that it has a strong anti-inflammatory effect and can partially inhibit DSS-mediated colitis in mice.

## Discussion

Melianodiol (MN), the main component of *M. azedarach* fruit, has been reported to have anti-inflammatory and anti-cancer effects (Akihisa et al., 2017). In this study, we explored the anti-UC effect and mechanisms of action of MN. We first evaluated the therapeutic activity of MN in DSS-mediated UC. The results indicated that MN improved weight loss, colonic contracture, and DAI scores in UC mice in a dose-dependent manner. Histopathological findings indicated that MN (100 and 200 mg/kg) notably decreased DSS-mediated inflammatory infiltration, oedema, and exudation of eosinophils and neutrophils, indicating that MN has good therapeutic activity in mice with ulcerative colitis.

We also tried to fully explore the activity of MN on the inflammation of RAW264.7 cells *in vitro* through further experiments. Our results showed that MN notably decreased the secretion level of NO, IL-10, IL-6, and TNF-*α* in LPS-mediated RAW264.7 cells (Figs. 3A–3D). Simultaneously, as the dose of MN increased (from 50 to 200 μg/mL), the secretion of NO, IL-6, and TNF-*α* in macrophages gradually decreased, whereas the secretion of IL-10 gradually increased, suggesting that MN helps reduce the inflammatory response of RAW264.7 cells mediated with LPS.

To a certain degree, IBD is considered an autoimmune disease such as psoriasis, lupus erythematosus, and rheumatoid arthritis (Moller et al., 2015). Pro-inflammatory cytokines such as IL-1*β* and TNF-*α* play a vital role in inflammation. However, excessive synthesis and secretion of pro-inflammatory factors such as IL-1*β* and TNF-*α* often cause injury to the body (Silva et al., 2017). IL-10 is the main anti-inflammatory factor in the body. Studieshave demonstrated that IL-10-lacking mice could voluntarily develop UC, indicating that IL-10 plays a key role in bowel immune modulation (Ip et al., 2017). The results indicated that the levels of IL-1*β* and TNF-*α* in the colon tissue of the DSS group were notably improved and IL-10 was notably reduced (Figs. 5A–5C), indicating the significance of inflammatory responses in the model group. As expected, MN (100 and 200 mg/kg) notably decreased TNF-*α* and IL-1*β* levels and notably improved the IL-10 level in the colon tissues of mice (Figs. 5A–5C), suggesting that MN has notable anti-inflammatory effects and can inhibit abnormal immune responses.

In addition, to study the anti-inflammatory mechanisms of MN, we detected NF-*κ*B signalling cascade proteins in mouse colon tissues and RAW264.7 cells. Studies have exhibited that the NF-*κ*B signalling cascade is unusually activated in a DSS-mediated UC mouse model (You et al., 2017; Noort, Tak & Tas, 2015). When the body is irritated by an exogenous substance or pro-inflammatory cytokines, the relevant receptor on the cell membrane is activated and the signal is transferred to the cytoplasm to phosphorylate I*κ*B kinase, which further catalyses the phosphorylation of I*κ*B. In the nucleus, NF-*κ*B binds to a specific DNA sequence, which in turn promotes the transcription of genes in the cell and encodes large amounts of inflammatory response proteins, thereby promoting the inflammatory response (Mi & Jin, 2016). The results indicate that compared to the control group, NF-*κ*B P65, p-IKK*β*, and p-I*κ*B*α* levels were notably increased in colon tissues and LPS-induced RAW264.7 cells in the model group (Figs. 6A–6G), indicating that the NF-*κ*B signalling cascade is notably activated in the model group mice and LPS-induced RAW264.7 cells. MN treatment notably reduced the levelof these proteins and had notable anti-inflammatory effects (Figs. 6A–6G). The anti-inflammatory mechanisms of MN may be to reduce the phosphorylation of NF-*κ*B P65 and I*κ*B*α* by inhibiting the phosphorylation of IKK kinase in the colon, thus reducing the hydrolysis of the NF-*κ*B/I*κ*B complex. To reduce the free activated NF-*κ*B to enter the nuclear membrane, reduce the downstream NO, TNF-*α*, IL-1β, and increase the transcription and expression of factors such as IL-10, reduce the inflammatory response, and regulate the body’s immune homeostasis. These results suggest that MN can inhibit DSS-mediated inflammatory responses in UC mice and LPS-treated RAW264.7 cells inflammation by suppressing the NF-*κ*B signaling cascade.

Experimental data show that, in addition to inflammation, mice with colitis also have significant oxidative stress (Almeer et al., 2018). Therefore, this antioxidant strategy also has broad application prospectsfor UC. In this study, we demonstrated notable oxidative stress in DSS-mediated UC mice by measuring oxidative stress parameters in the colon tissues of mice. MN intervention can notably decrease the content of NO and MDA in colon tissues of UC mice and improve the levels of GSH and the effect of SOD antioxidant enzyme (Figs. 3A–3D), indicating that the therapeutic effects of MN on UC are due to its antioxidant activity.

The Nrf-2 signalling cascade is a security system that usually modulates the expressions of antioxidant protein in the body. In the cell nucleus, Nrf-2 is heterodimerized with small proteins Maf or Jun, which links with the antioxidant response element ARE and causes the expression of antioxidant proteins to remove oxidants, thus keeping cells from oxidative stress injury (Amini et al., 2019). It has been reported that the Nrf-2 signalling cascade is remarkably suppressed in UC rats (Saber et al., 2019). Coherent with this report, the results of our study indicated that Nrf2 expression was notably decreased in the colon tissues of the DSS group. Our results indicated that the Keap1 level in UC mice was remarkably decreased, while MN notably reversed these abnormal changes (Figs. 8A–8D).

Moreover, JAK2/STAT3 signalling are the two main pathways, including transcription factors involved in pro-inflammatory cytokine responses in IBD intestinal mucosal inflammation (Coskun et al., 2013; Takada et al., 2010). Proinflammatory cytokines are basic moderators of colitis development (Nguyen, Putoczki & Ernst, 2015). The improved expression of colonic cytokines, JAK2, and STAT3 was closely related to the severity of DSS-induced colitis. The main finding of our study was the observed reduction in JAK2/STAT3 phosphorylation after MN treatment, indicating that inhibition of the JAK and STAT pathways may be involved in MN treatment of colitis, which is associated with pro-inflammatory cytokine gene expression. At the molecular level, MN-induced pro-inflammatory cytokines are downregulated, which is supplemented by the downregulation of the downstream STAT3 signaling pathway. MN downregulates STAT3 phosphorylation, which may be related to the disruption of its upstream kinase JAK.

iNOS is a marker of inflammation, and several previous studies have shown that in DSS models (Beck et al., 2004), iNOS is upregulated in mucosal samples, indicating ulcerative colitis (Palatka et al., 2005). In an animal model of ulcerative colitis (Miller et al., 1995), iNOS was defined as infiltrating macrophages and neutrophils in the colonic mucosa and submucosa (Palatka et al., 2005). The increase in iNOS expression in tissues is associated with disease severity (Vento et al., 2001). Recent results indicate that eNOS may be related to the preservation of the colon from mucosal inflammation due to increased disease activity induced by DSS in eNOS-deficient mice (Sasaki et al., 2003). Animals lacking eNOS had more severe colitis. This study found that MN improved the expression of eNOS and reduced the expression of iNOS in colitis tissues, suggesting that MN can suppress the apoptosis of colitis mucosal epithelial cells and plays a biological role, and that there is a positive correlation with concentration.

## Conclusion

Our results suggested that MN administration improves DSS-mediated colitis in mice. The protective activity of MN seems to be related to the reduction in inflammation and oxidative stress by attenuating pro-inflammatory cytokines induced by JAK and STAT, iNOS/eNOS, and NF-*κ*B signaling cascades. These results indicate that MN may be an effective botanical drug with advantages in prospective clinical applications for the treatment of IBD or related diseases in the future.

## Supplemental Information

10.7717/peerj.14209/supp-1 Supplemental Information 1 ARRIVE 2.0 Checklist.

10.7717/peerj.14209/supp-2 Supplemental Information 2 Fig. 2 raw data. Weight growth, colonic length, DAI Score

10.7717/peerj.14209/supp-3 Supplemental Information 3 Fig. 3 raw data. NO, TNF-α, IL-6, IL-10

10.7717/peerj.14209/supp-4 Supplemental Information 4 Fig. 4 raw data. NO, GSH, MDA, SOD

10.7717/peerj.14209/supp-5 Supplemental Information 5 Fig. 5 raw data. IL-1β, IL-10, TNF-a

10.7717/peerj.14209/supp-6 Supplemental Information 6 Fig. 6 raw data original Western Blot images. Immunofluorescence analysis of NF-kB

10.7717/peerj.14209/supp-7 Supplemental Information 7 Fig. 7 raw data original Western Blot images.

10.7717/peerj.14209/supp-8 Supplemental Information 8 Fig. 8 raw data original Western Blot images. Immunofluorescence analysis of Nrf-2

10.7717/peerj.14209/supp-9 Supplemental Information 9 Fig. 9 raw data original Western Blot images.

10.7717/peerj.14209/supp-10 Supplemental Information 10 Fig. 10 raw data original Western Blot images.

10.7717/peerj.14209/supp-11 Supplemental Information 11 Fig. 11 raw data. H&E data

10.7717/peerj.14209/supp-12 Supplemental Information 12 Supplemental Figures.

## Additional Information and Declarations

### Data Availability

The following information was supplied regarding data availability: The raw measurements are available in the Supplemental Files.
