# Supplementary figures and images for: Anti-inflammatory and anti-oxidant properties of Melianodiol on DSS-induced ulcerative colitis in mice
Source: PeerJ. 2022 Oct 25;10:e14209. doi: 10.7717/peerj.14209 (PMC9615967; doi:10.7717/peerj.14209)

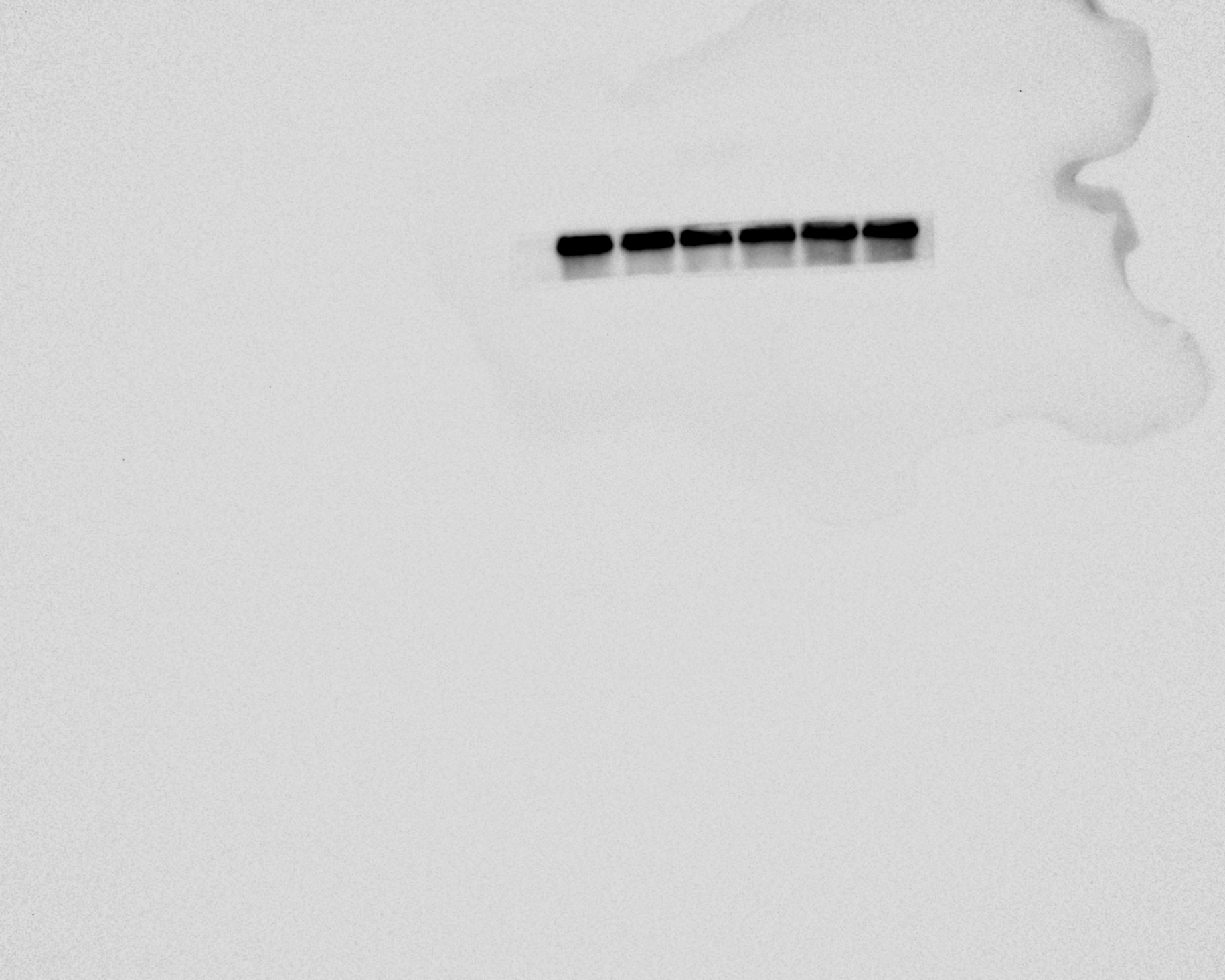

Supplement: Supplemental Information 6 — Immunofluorescence analysis of NF-kB [file peerj-10-14209-s006.zip › Fig. 6 raw data/Figure 6A original Western Blot images/Figure6A,8A,9A,10A a┬-actin.jpg]

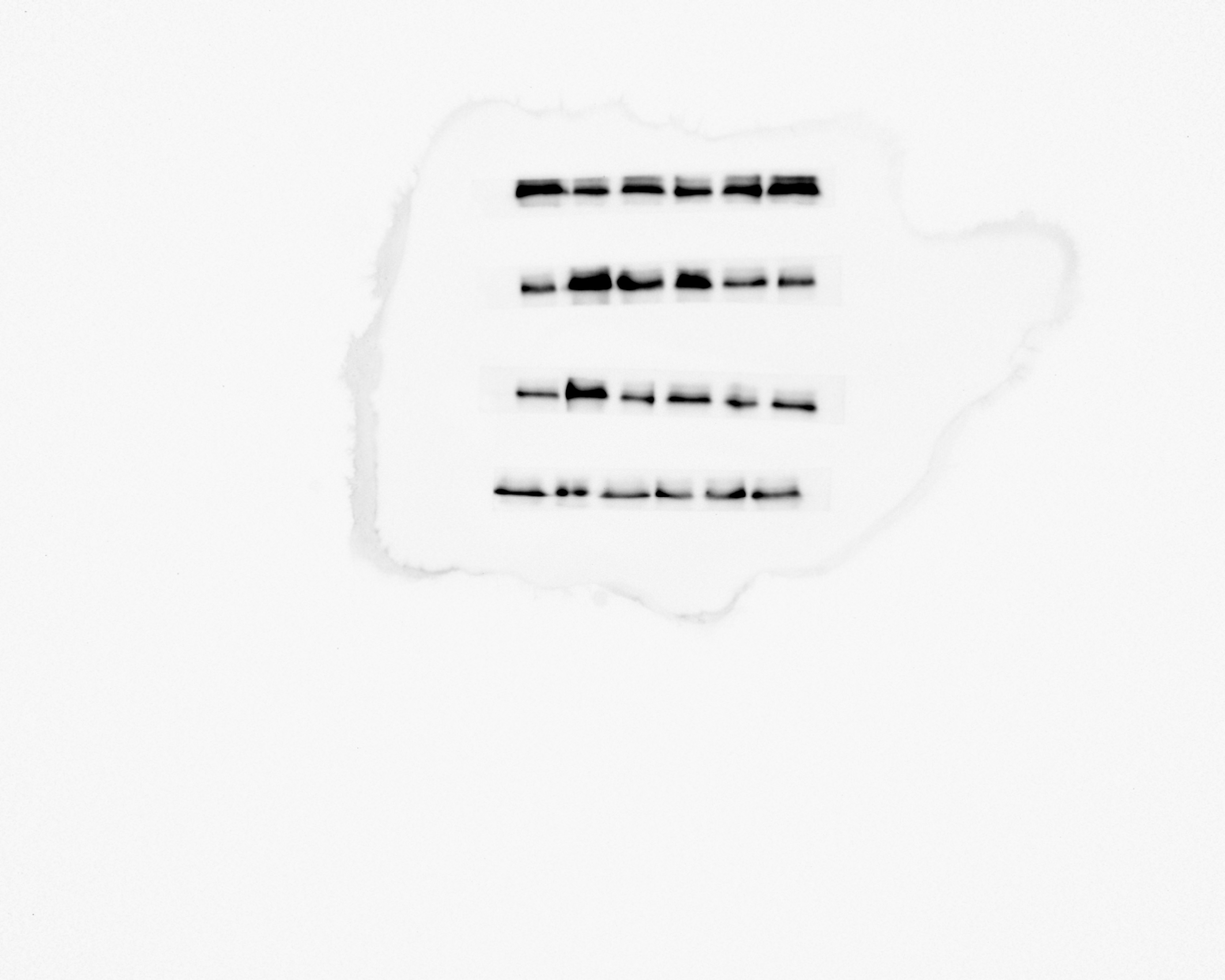

Supplement: Supplemental Information 6 — Immunofluorescence analysis of NF-kB [file peerj-10-14209-s006.zip › Fig. 6 raw data/Figure 6A original Western Blot images/IKB-a┴ First Blot from the top down.jpg]

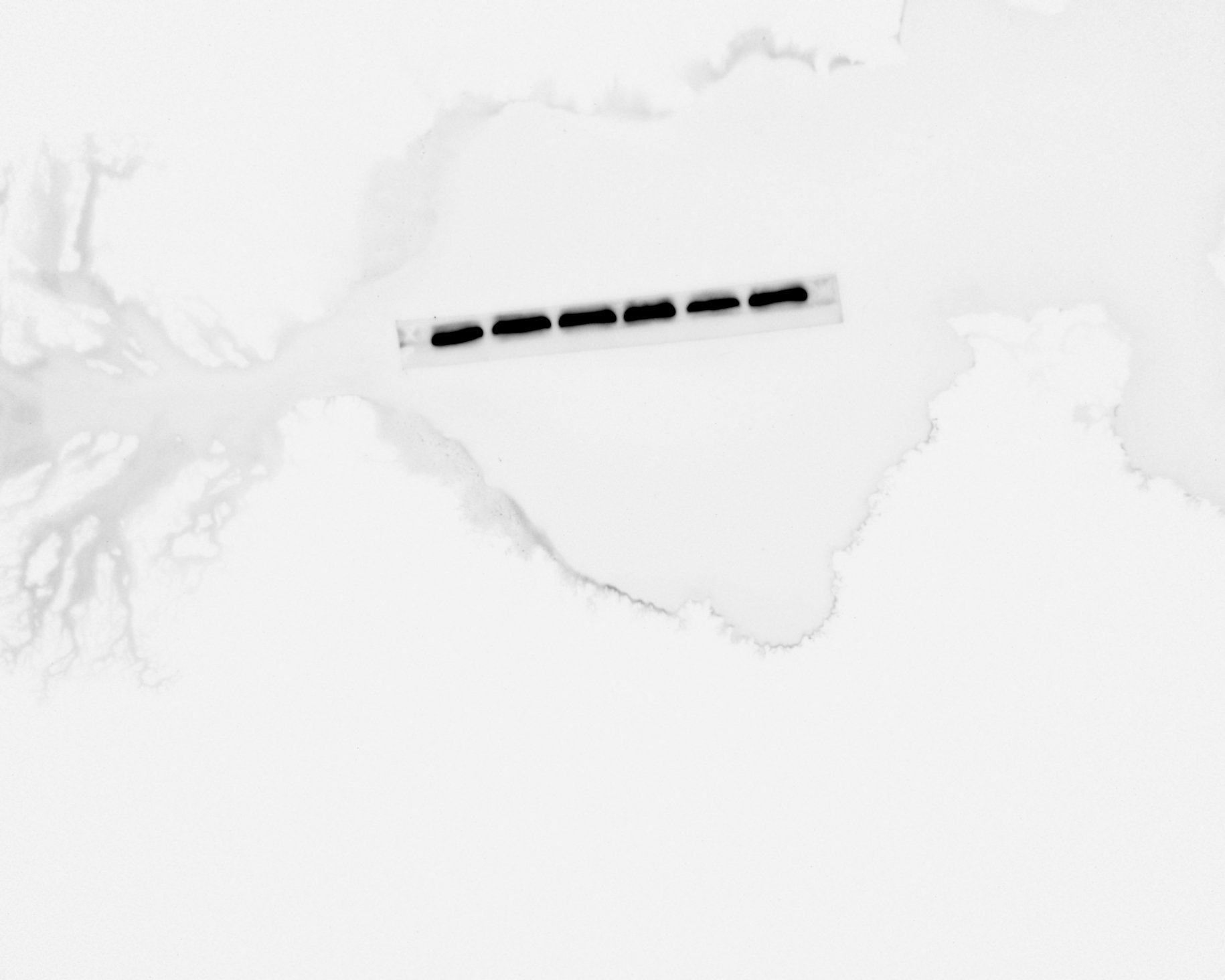

Supplement: Supplemental Information 6 — Immunofluorescence analysis of NF-kB [file peerj-10-14209-s006.zip › Fig. 6 raw data/Figure 6A original Western Blot images/IKKa┴a┬.jpg]

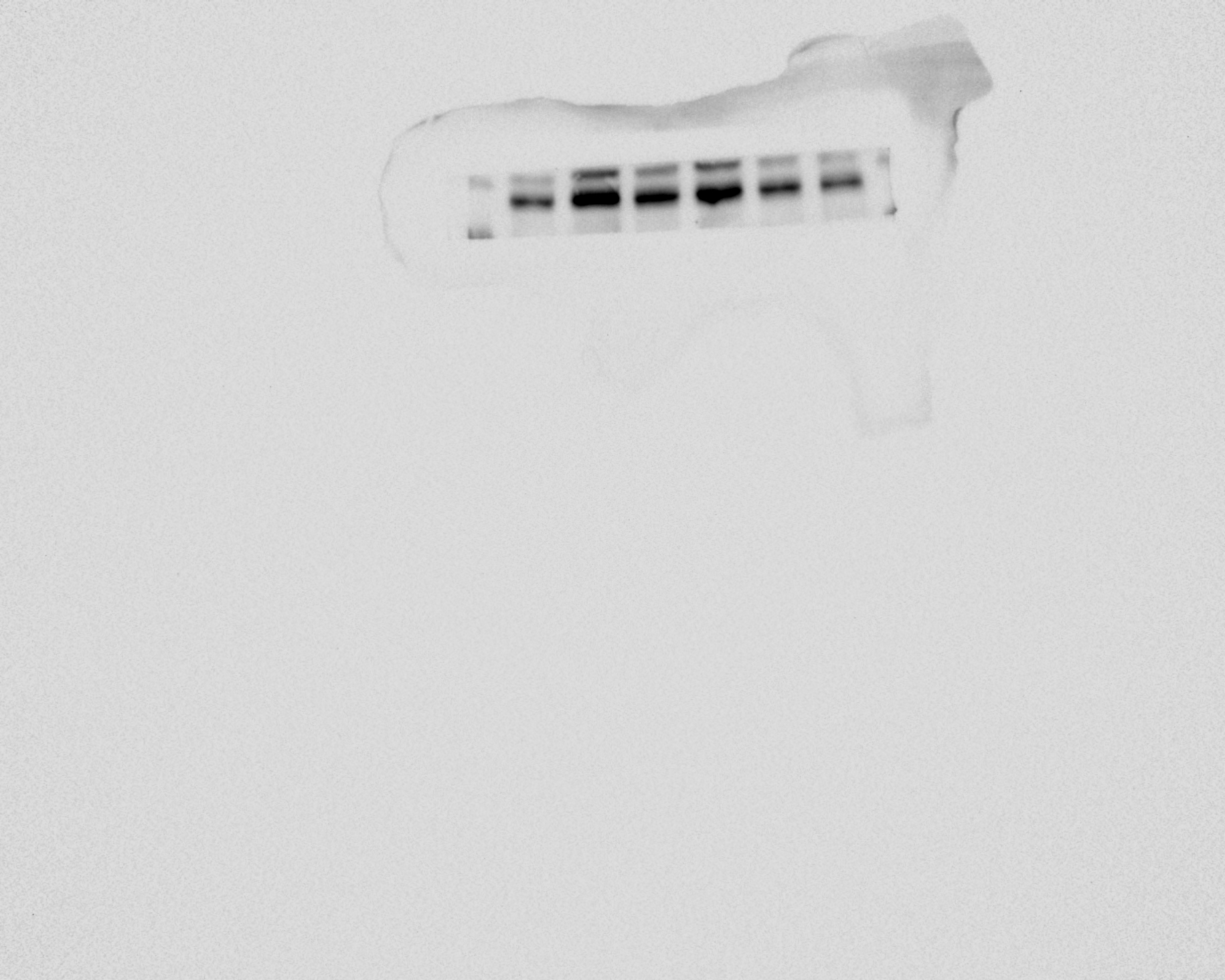

Supplement: Supplemental Information 6 — Immunofluorescence analysis of NF-kB [file peerj-10-14209-s006.zip › Fig. 6 raw data/Figure 6A original Western Blot images/P65.jpg]

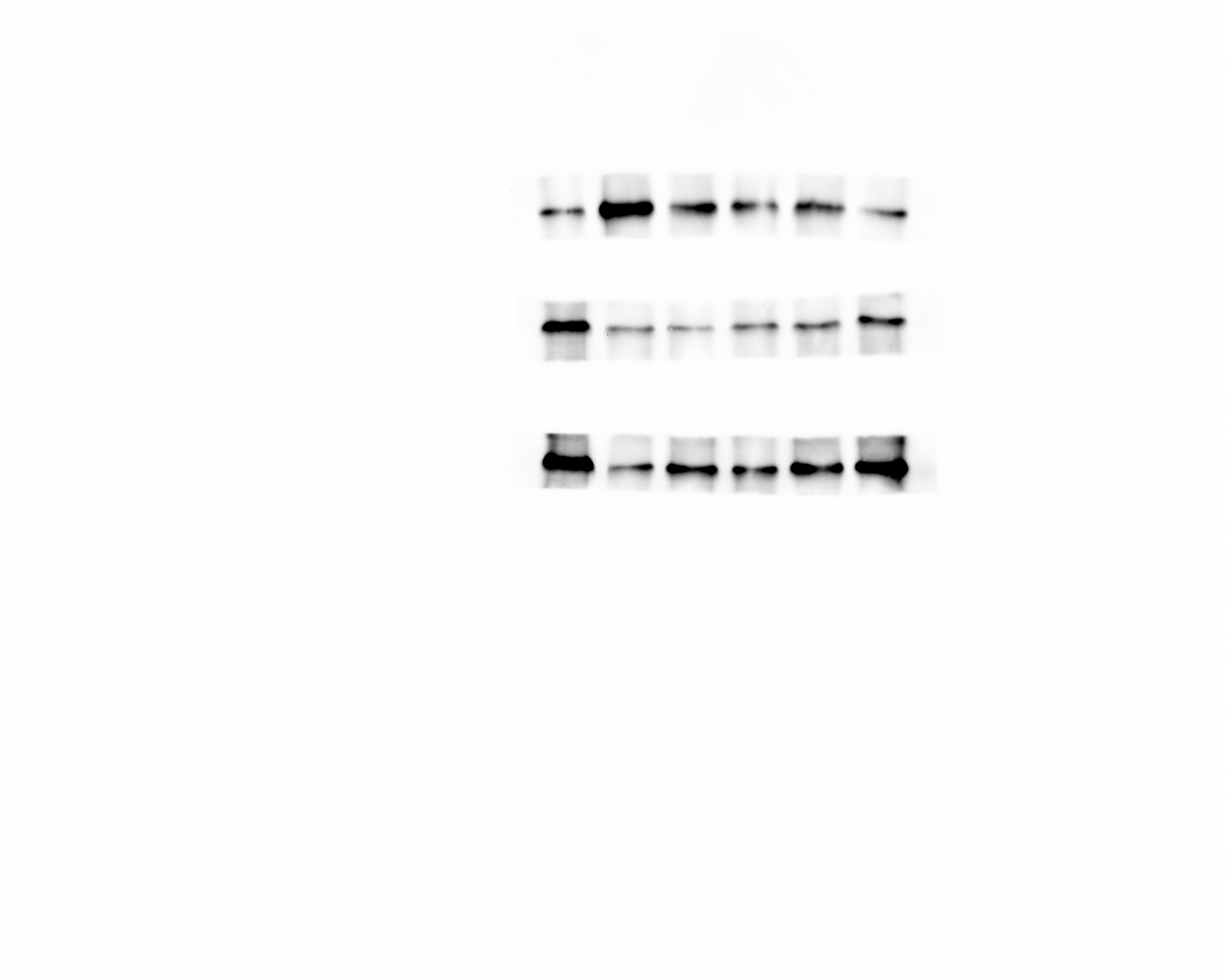

Supplement: Supplemental Information 6 — Immunofluorescence analysis of NF-kB [file peerj-10-14209-s006.zip › Fig. 6 raw data/Figure 6A original Western Blot images/pIKB-a┴ First Blot from the top down.jpg]

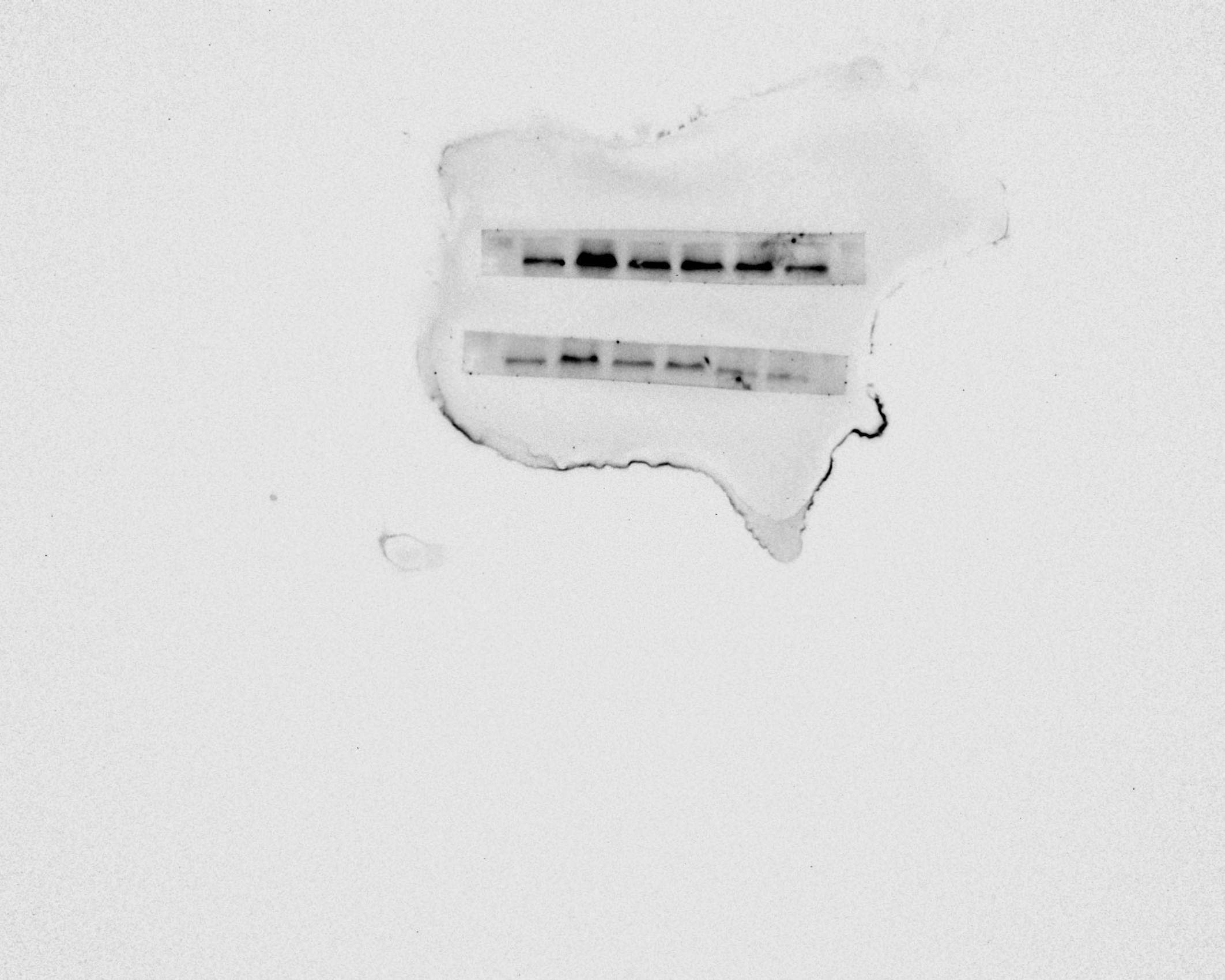

Supplement: Supplemental Information 6 — Immunofluorescence analysis of NF-kB [file peerj-10-14209-s006.zip › Fig. 6 raw data/Figure 6A original Western Blot images/p-IKKa┴,a┬ First Blot from the top down.jpg]

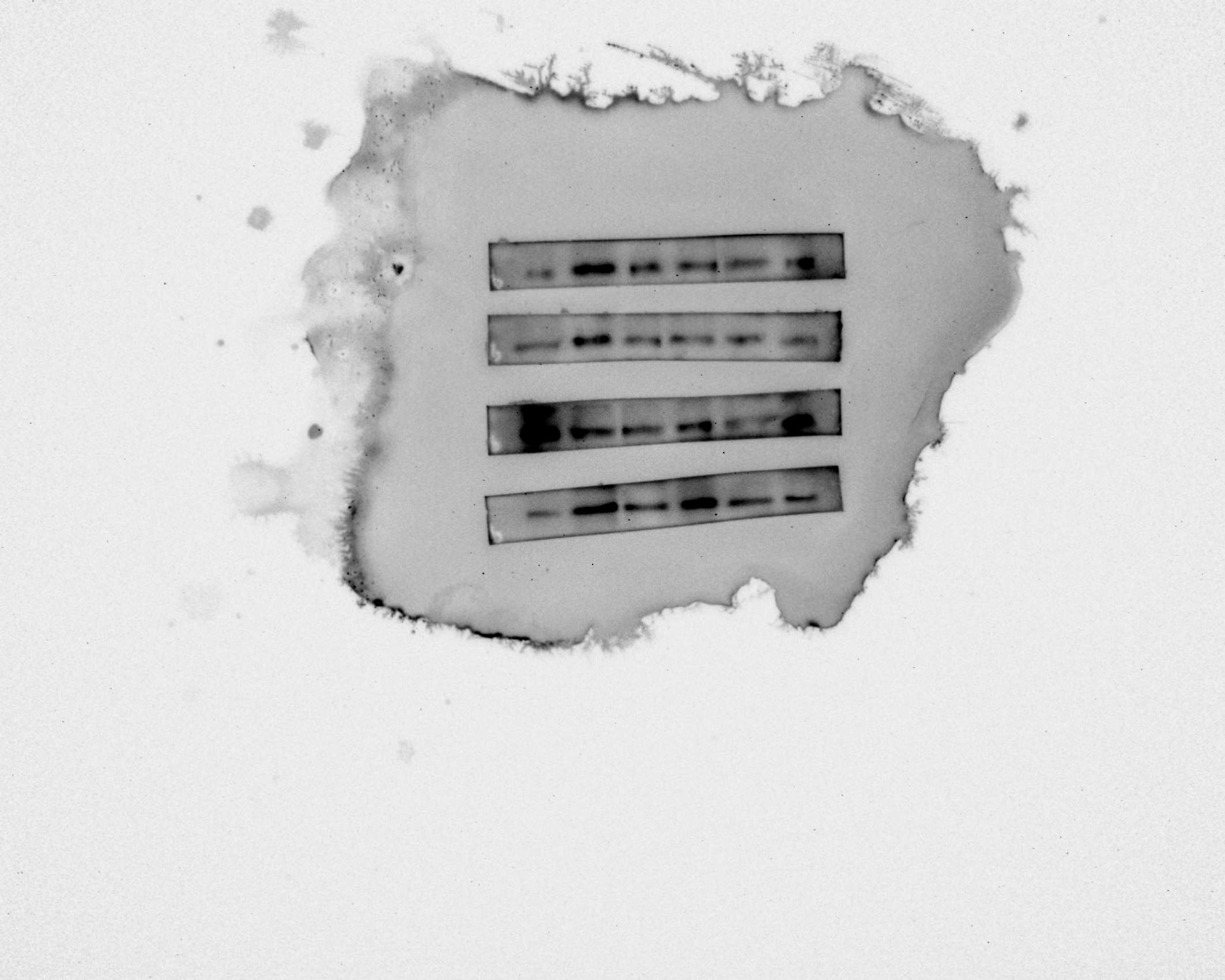

Supplement: Supplemental Information 6 — Immunofluorescence analysis of NF-kB [file peerj-10-14209-s006.zip › Fig. 6 raw data/Figure 6A original Western Blot images/p-P65 Fourth Blot from the top down.jpg]

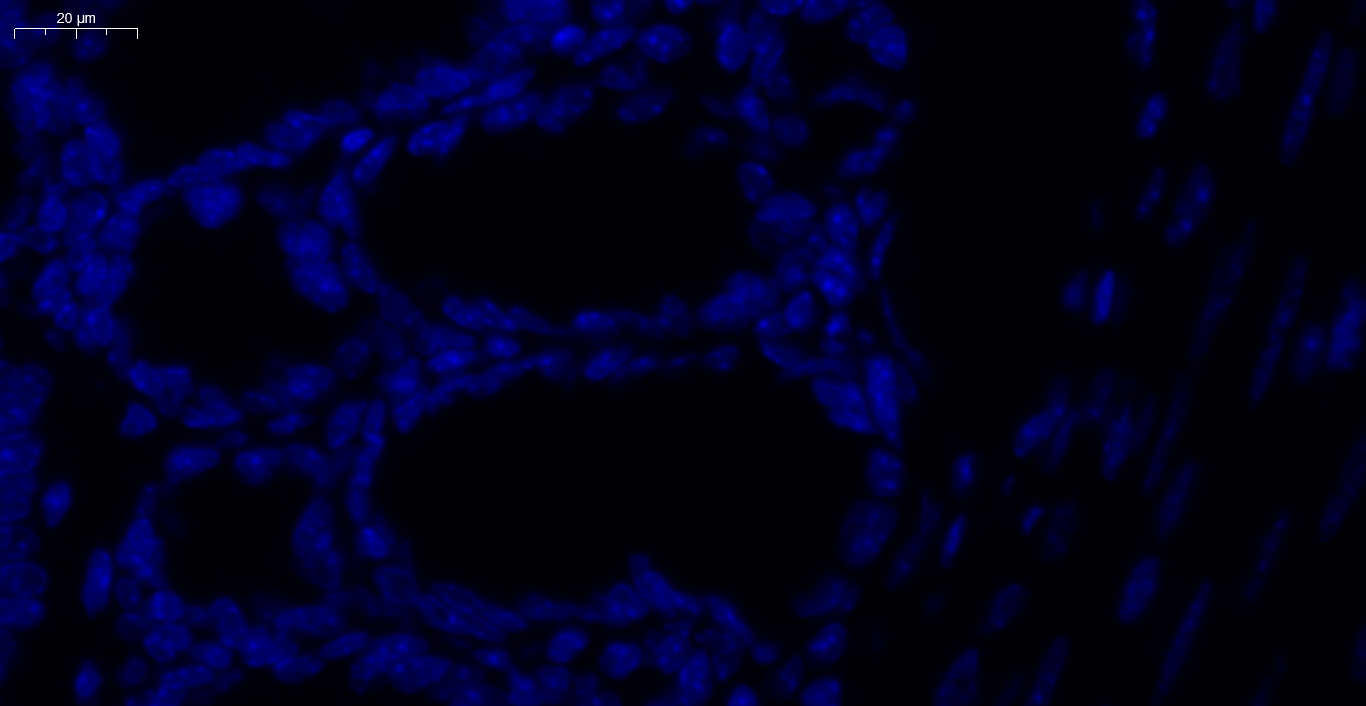

Supplement: Supplemental Information 6 — Immunofluorescence analysis of NF-kB [file peerj-10-14209-s006.zip › Fig. 6 raw data/Immunofluorescence analysis of NF-kB/The group of control/The DAPI picture of control group.jpg]

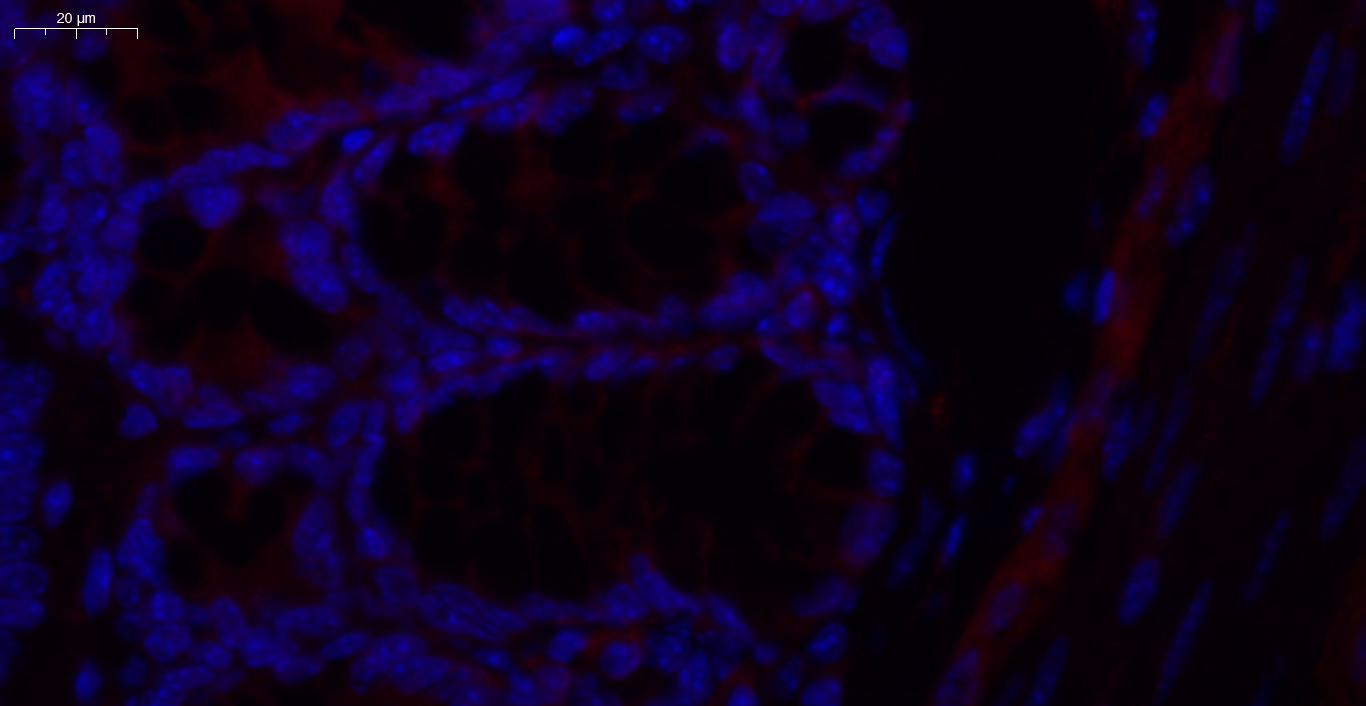

Supplement: Supplemental Information 6 — Immunofluorescence analysis of NF-kB [file peerj-10-14209-s006.zip › Fig. 6 raw data/Immunofluorescence analysis of NF-kB/The group of control/The Merge picture of control group.jpg]

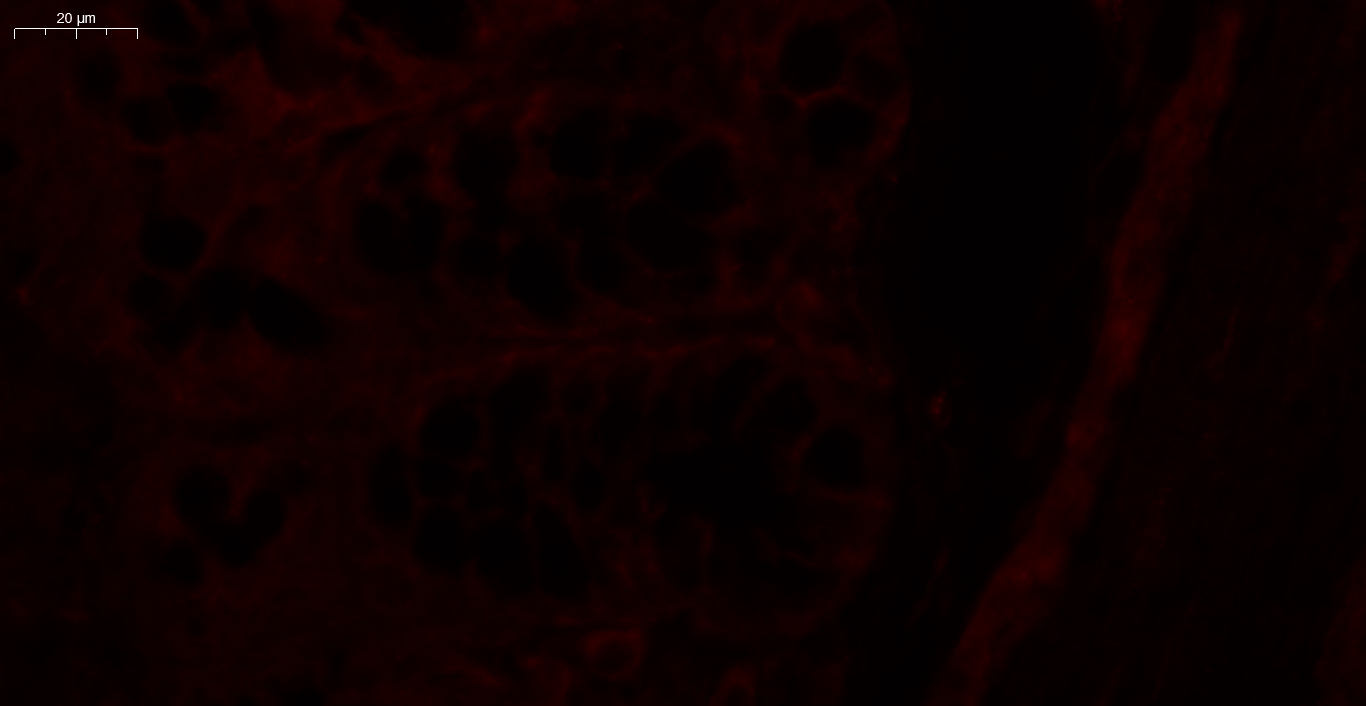

Supplement: Supplemental Information 6 — Immunofluorescence analysis of NF-kB [file peerj-10-14209-s006.zip › Fig. 6 raw data/Immunofluorescence analysis of NF-kB/The group of control/The NF-kB picture of control group.jpg]

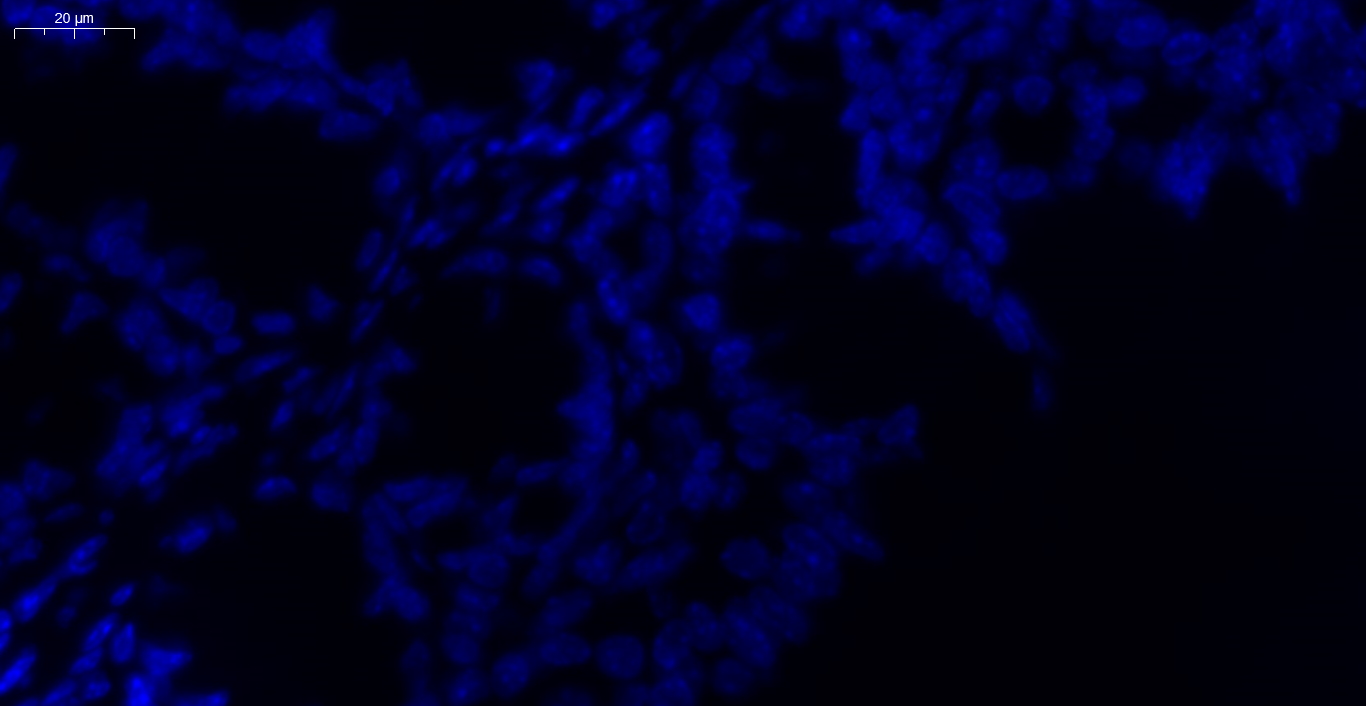

Supplement: Supplemental Information 6 — Immunofluorescence analysis of NF-kB [file peerj-10-14209-s006.zip › Fig. 6 raw data/Immunofluorescence analysis of NF-kB/The group of 100mg kg MN/The DAPI picture of 100 mg kg MN group.jpg]

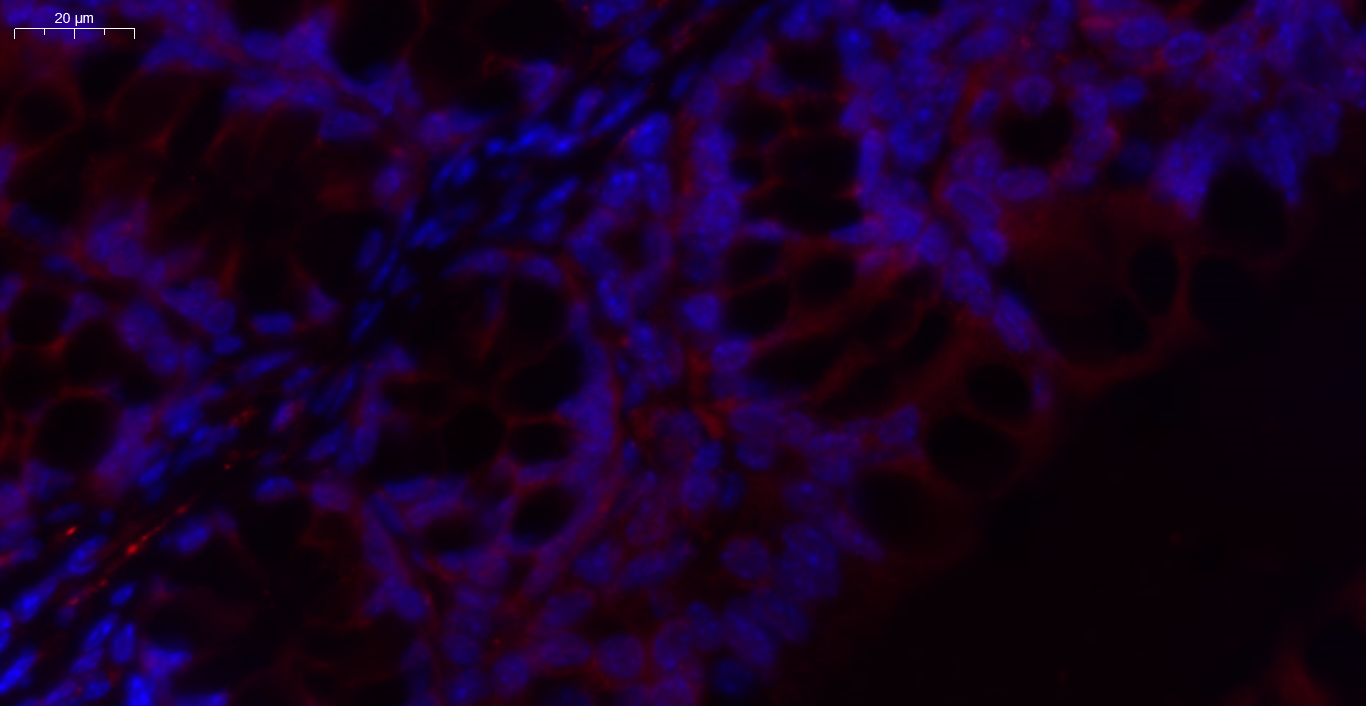

Supplement: Supplemental Information 6 — Immunofluorescence analysis of NF-kB [file peerj-10-14209-s006.zip › Fig. 6 raw data/Immunofluorescence analysis of NF-kB/The group of 100mg kg MN/The Merge picture of 100 mg kg MN group.jpg]

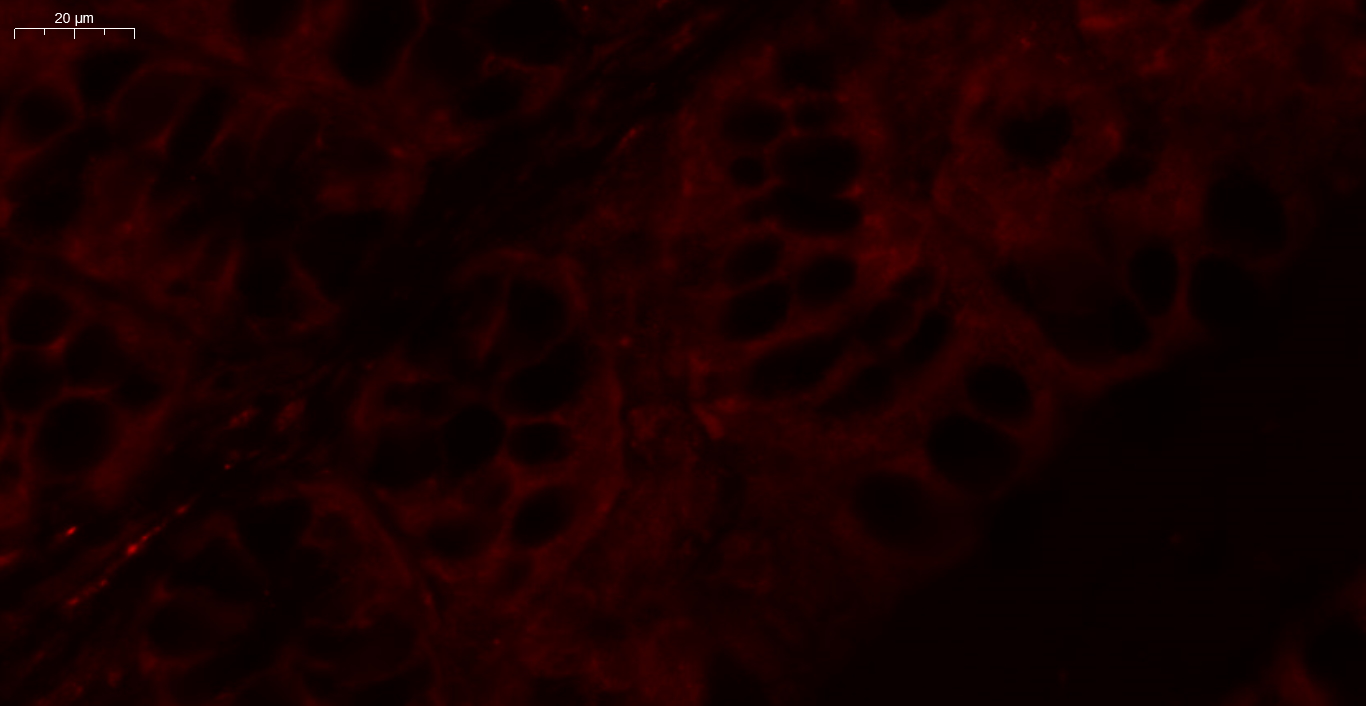

Supplement: Supplemental Information 6 — Immunofluorescence analysis of NF-kB [file peerj-10-14209-s006.zip › Fig. 6 raw data/Immunofluorescence analysis of NF-kB/The group of 100mg kg MN/The NF-a╩B picture of 100 mg kg MN group.jpg]

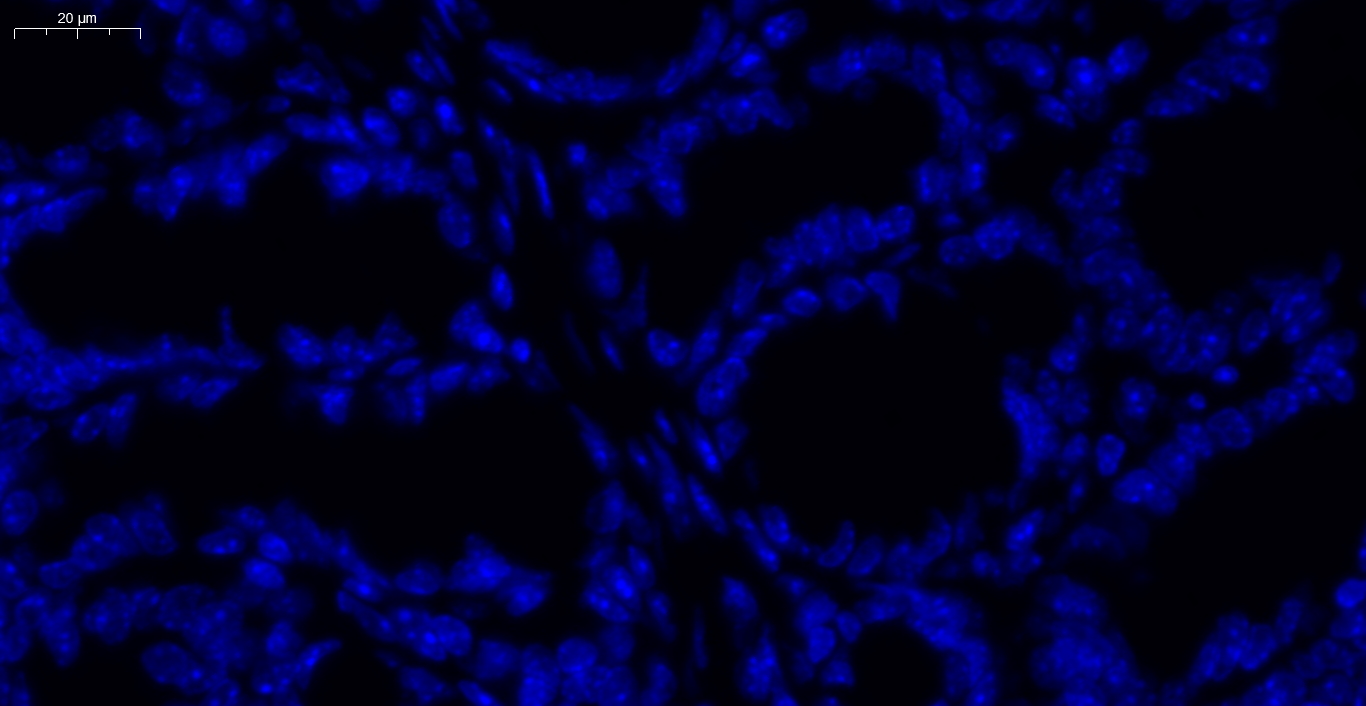

Supplement: Supplemental Information 6 — Immunofluorescence analysis of NF-kB [file peerj-10-14209-s006.zip › Fig. 6 raw data/Immunofluorescence analysis of NF-kB/The group of 200mg kg MN/The DAPI picture of 200mg kg MN group.jpg]

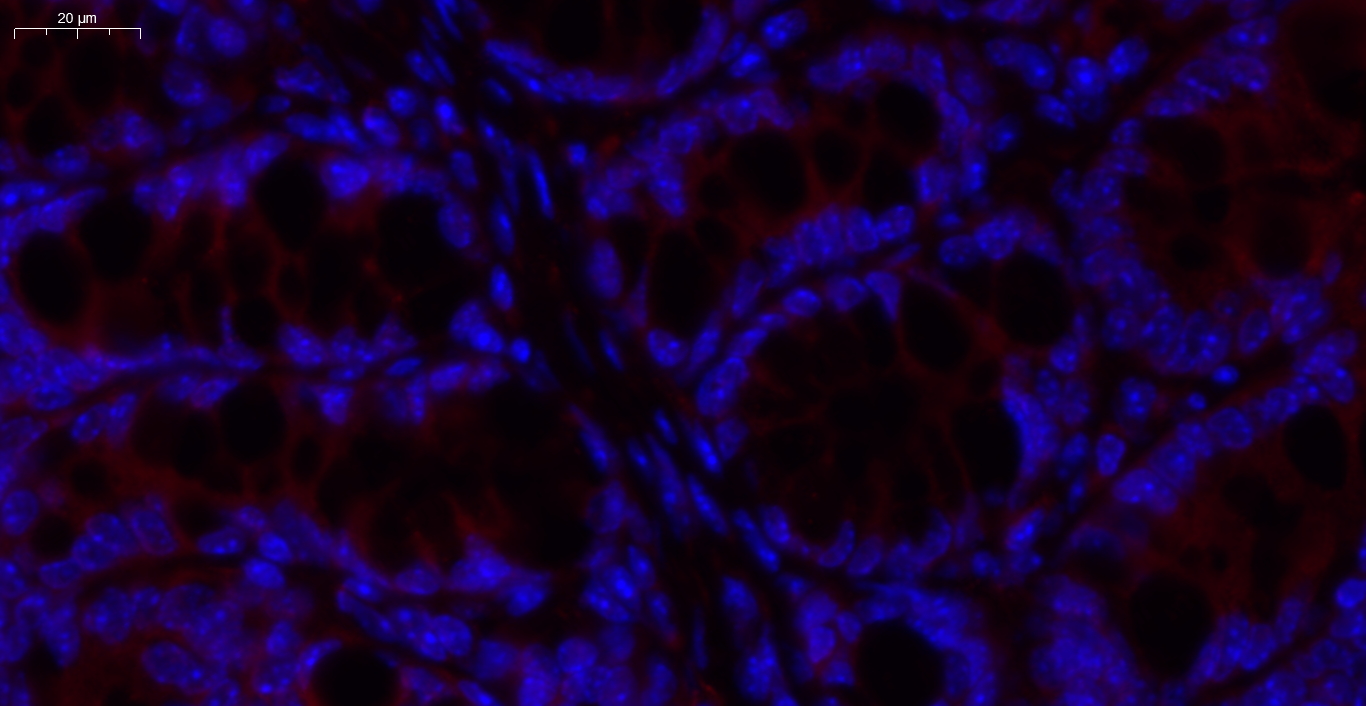

Supplement: Supplemental Information 6 — Immunofluorescence analysis of NF-kB [file peerj-10-14209-s006.zip › Fig. 6 raw data/Immunofluorescence analysis of NF-kB/The group of 200mg kg MN/The Merge picture of 200mg kg MN group.jpg]

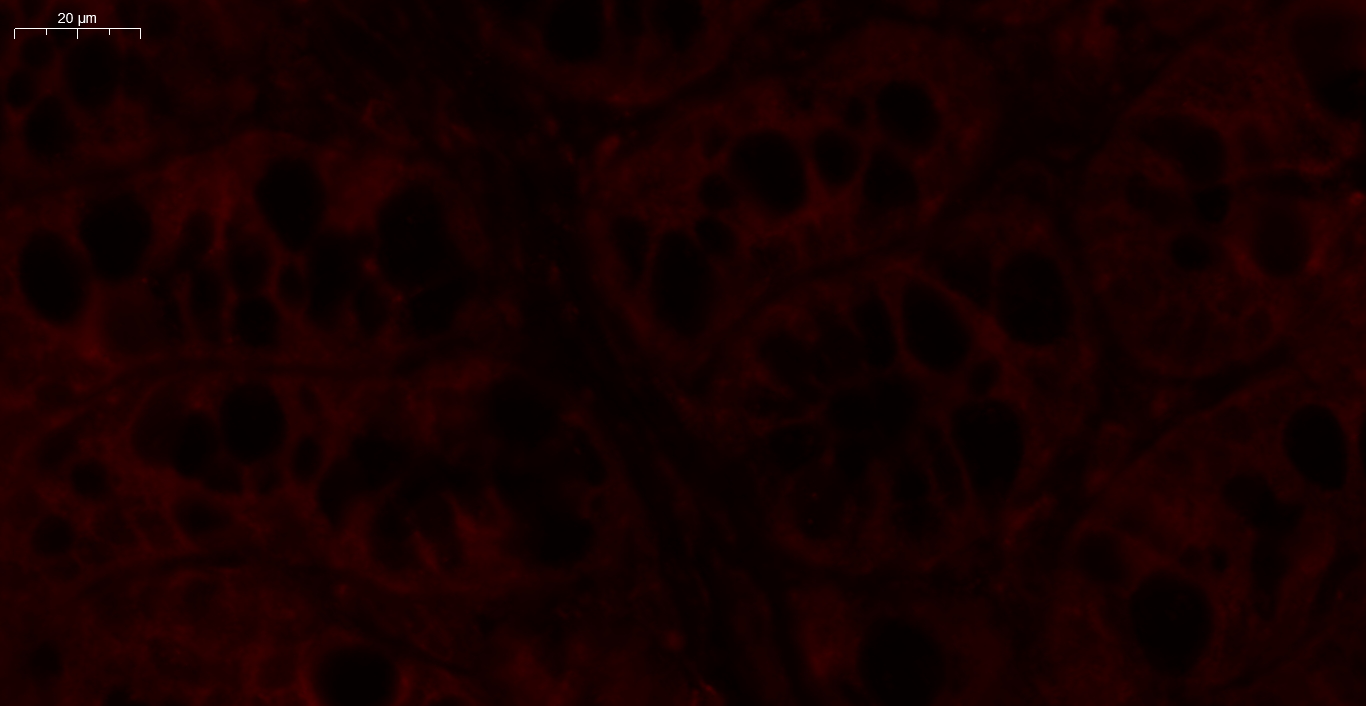

Supplement: Supplemental Information 6 — Immunofluorescence analysis of NF-kB [file peerj-10-14209-s006.zip › Fig. 6 raw data/Immunofluorescence analysis of NF-kB/The group of 200mg kg MN/The NF-kB picture of 200mg kg MN group.jpg]

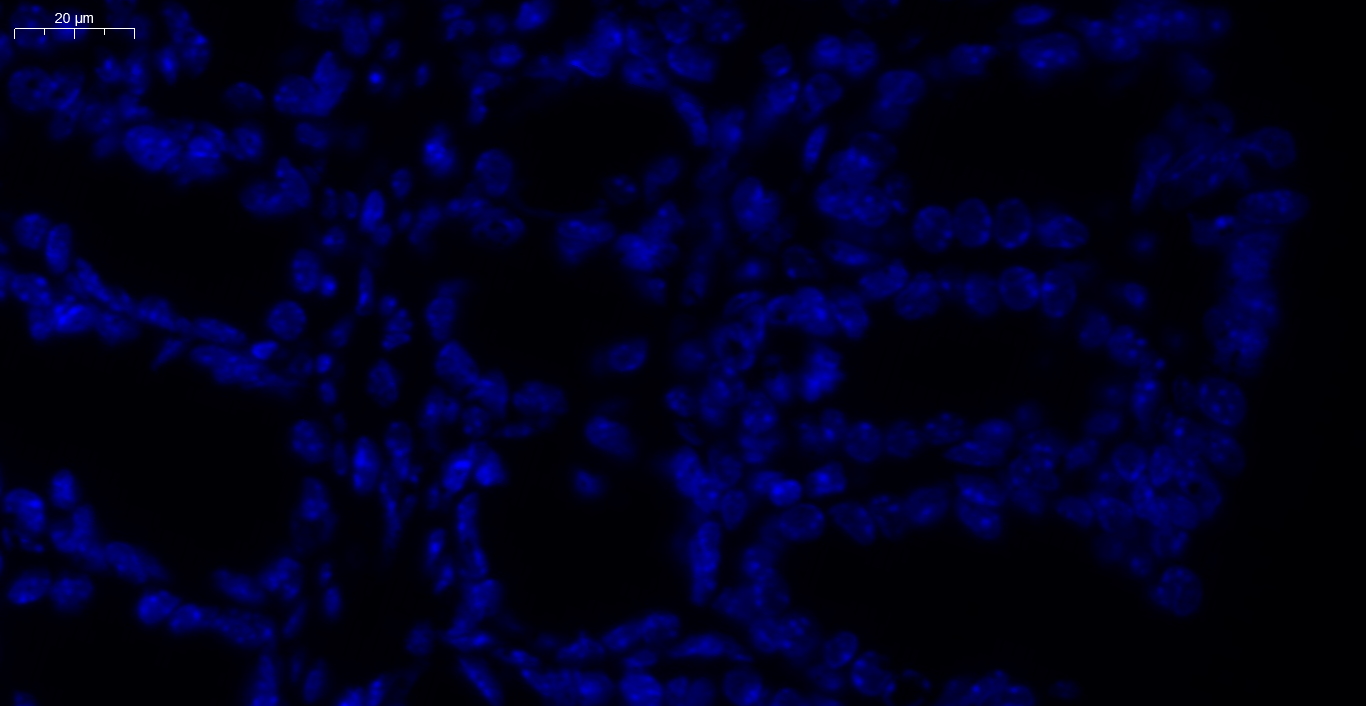

Supplement: Supplemental Information 6 — Immunofluorescence analysis of NF-kB [file peerj-10-14209-s006.zip › Fig. 6 raw data/Immunofluorescence analysis of NF-kB/The group of 500mg kg 5-ASA/The DAPI picture of 500mg kg 5-ASA group.jpg]

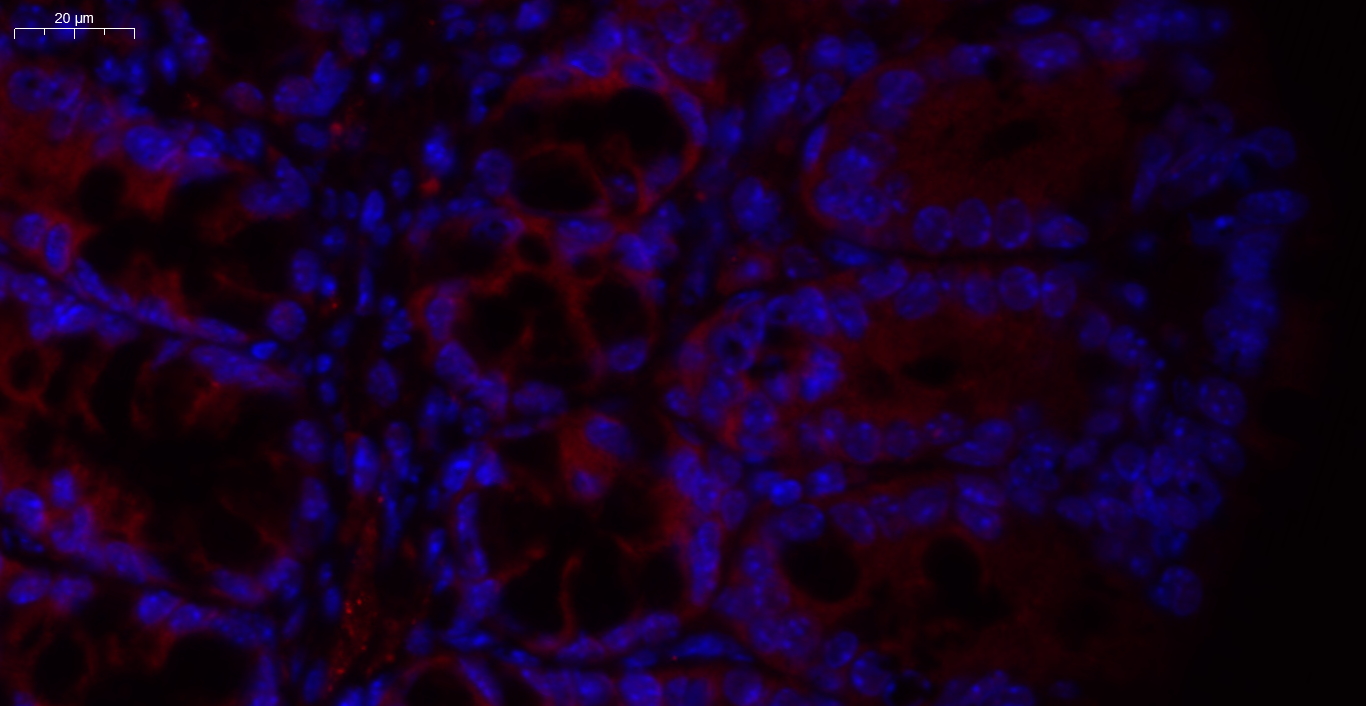

Supplement: Supplemental Information 6 — Immunofluorescence analysis of NF-kB [file peerj-10-14209-s006.zip › Fig. 6 raw data/Immunofluorescence analysis of NF-kB/The group of 500mg kg 5-ASA/The Merge picture of 500mg kg 5-ASA group.jpg]

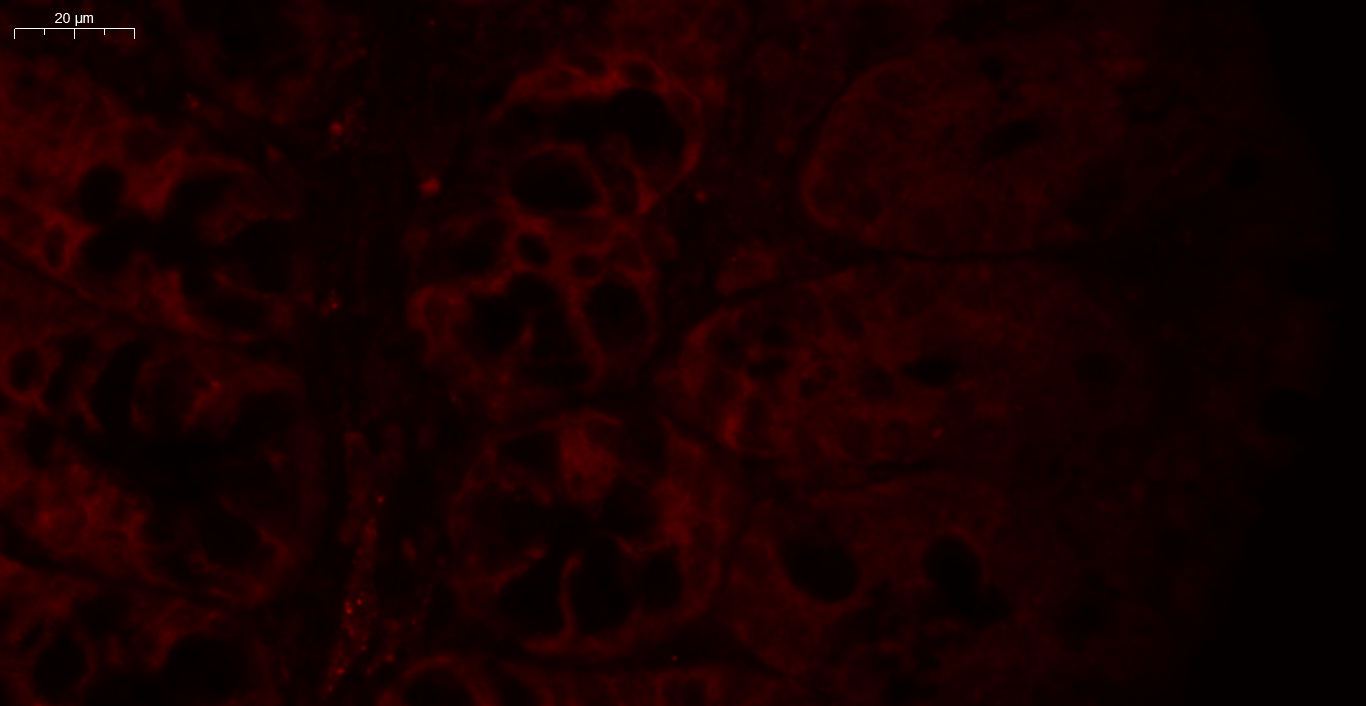

Supplement: Supplemental Information 6 — Immunofluorescence analysis of NF-kB [file peerj-10-14209-s006.zip › Fig. 6 raw data/Immunofluorescence analysis of NF-kB/The group of 500mg kg 5-ASA/The NF-kB picture of 500mg kg 5-ASA group.jpg]

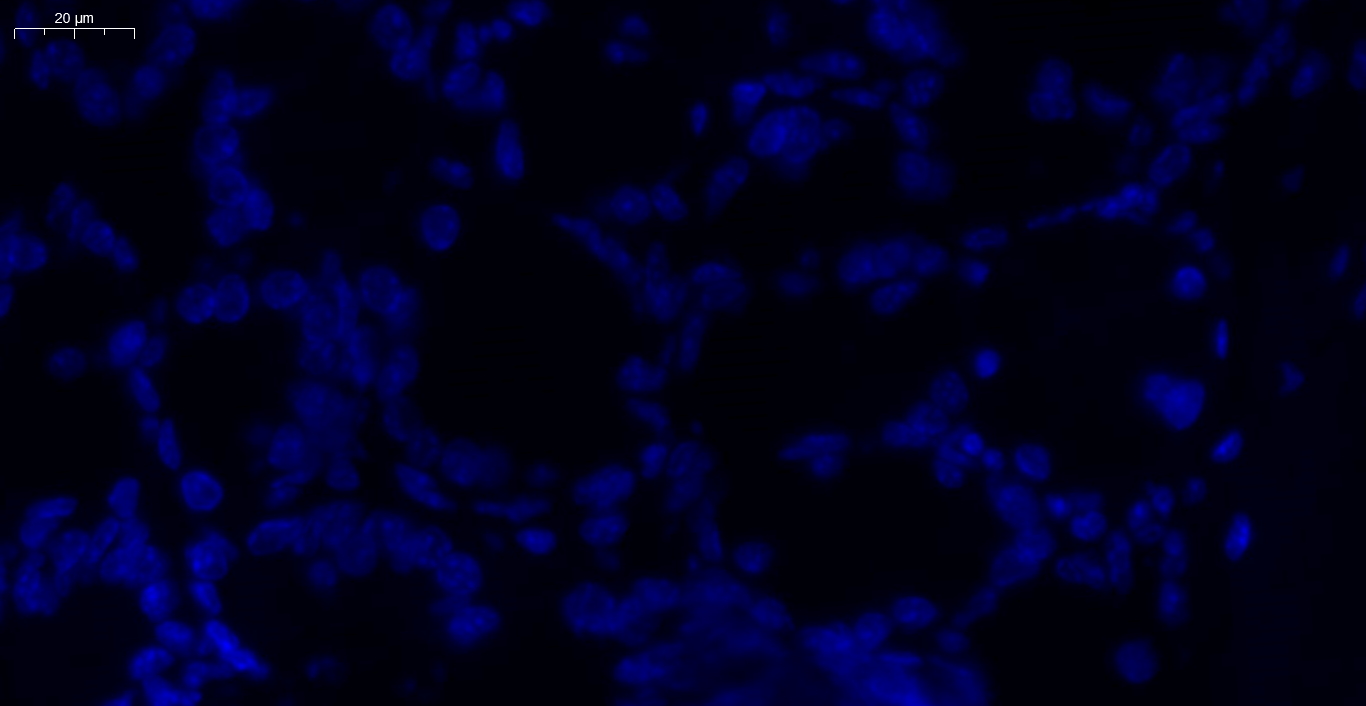

Supplement: Supplemental Information 6 — Immunofluorescence analysis of NF-kB [file peerj-10-14209-s006.zip › Fig. 6 raw data/Immunofluorescence analysis of NF-kB/The group of 50mg kg MN/The DAPI picture of 50 mg kg MN group.jpg]

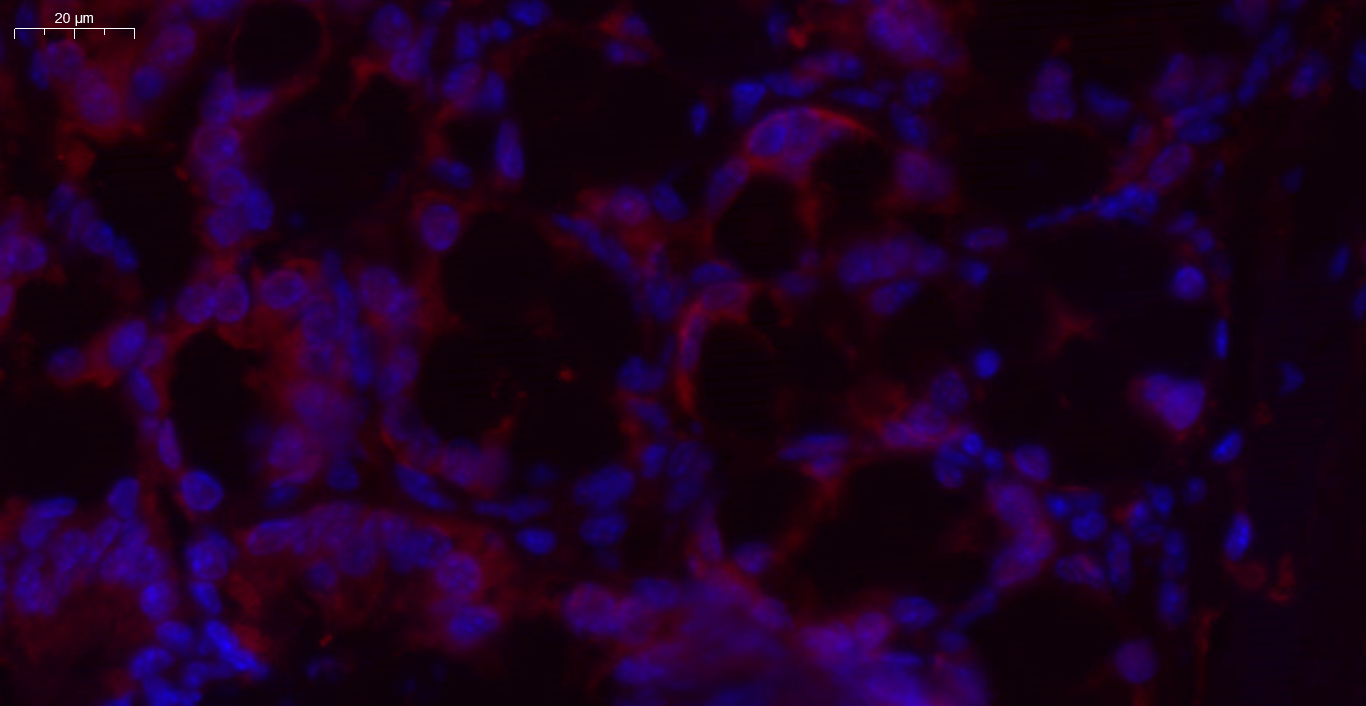

Supplement: Supplemental Information 6 — Immunofluorescence analysis of NF-kB [file peerj-10-14209-s006.zip › Fig. 6 raw data/Immunofluorescence analysis of NF-kB/The group of 50mg kg MN/The Merge picture of 50 mg kg MN group.jpg]

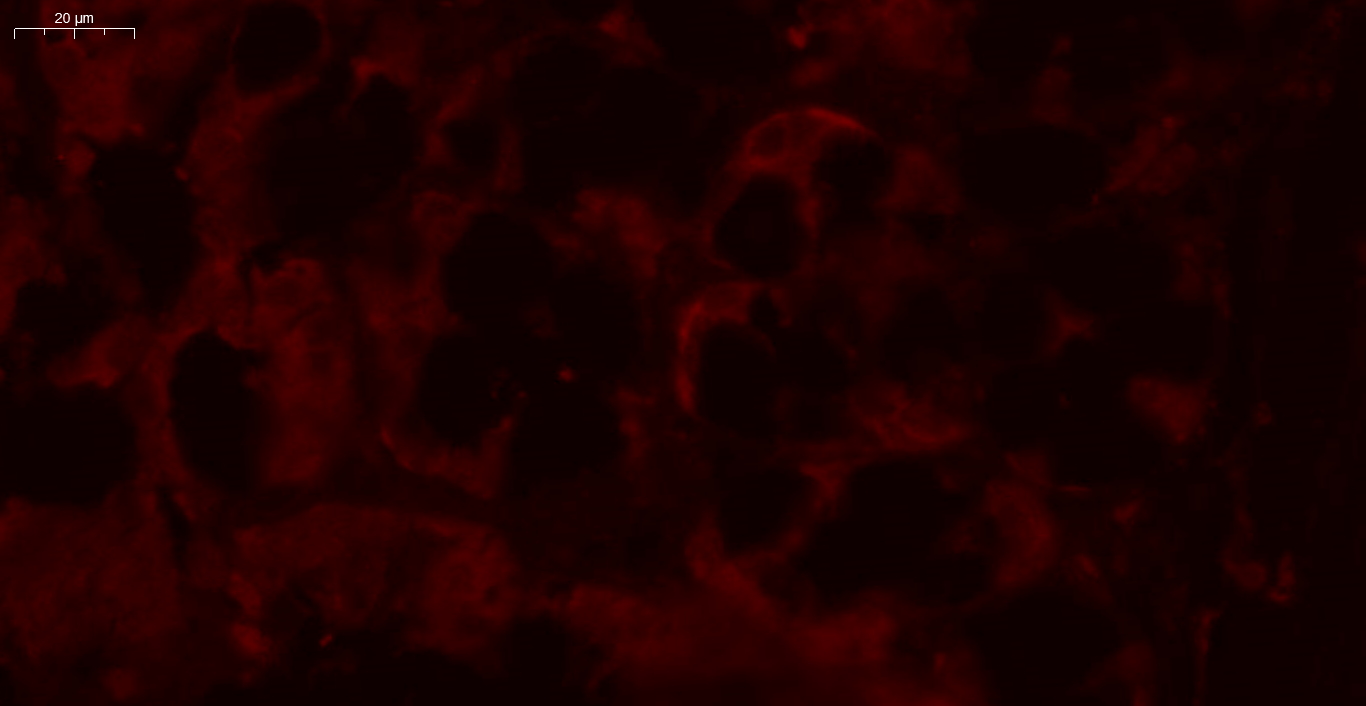

Supplement: Supplemental Information 6 — Immunofluorescence analysis of NF-kB [file peerj-10-14209-s006.zip › Fig. 6 raw data/Immunofluorescence analysis of NF-kB/The group of 50mg kg MN/The NF-kB picture of 50 mg kg MN group.jpg]

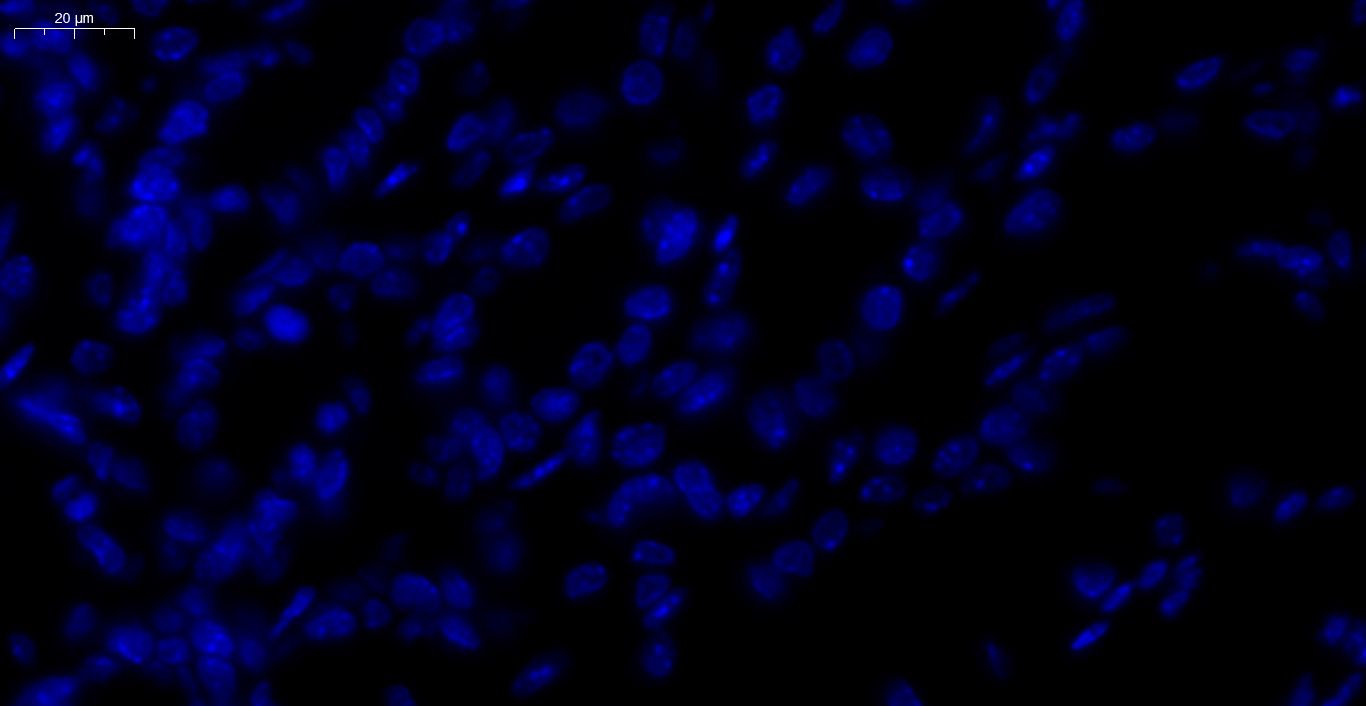

Supplement: Supplemental Information 6 — Immunofluorescence analysis of NF-kB [file peerj-10-14209-s006.zip › Fig. 6 raw data/Immunofluorescence analysis of NF-kB/The group of DSS/The DAPI picture of DSS group.jpg]

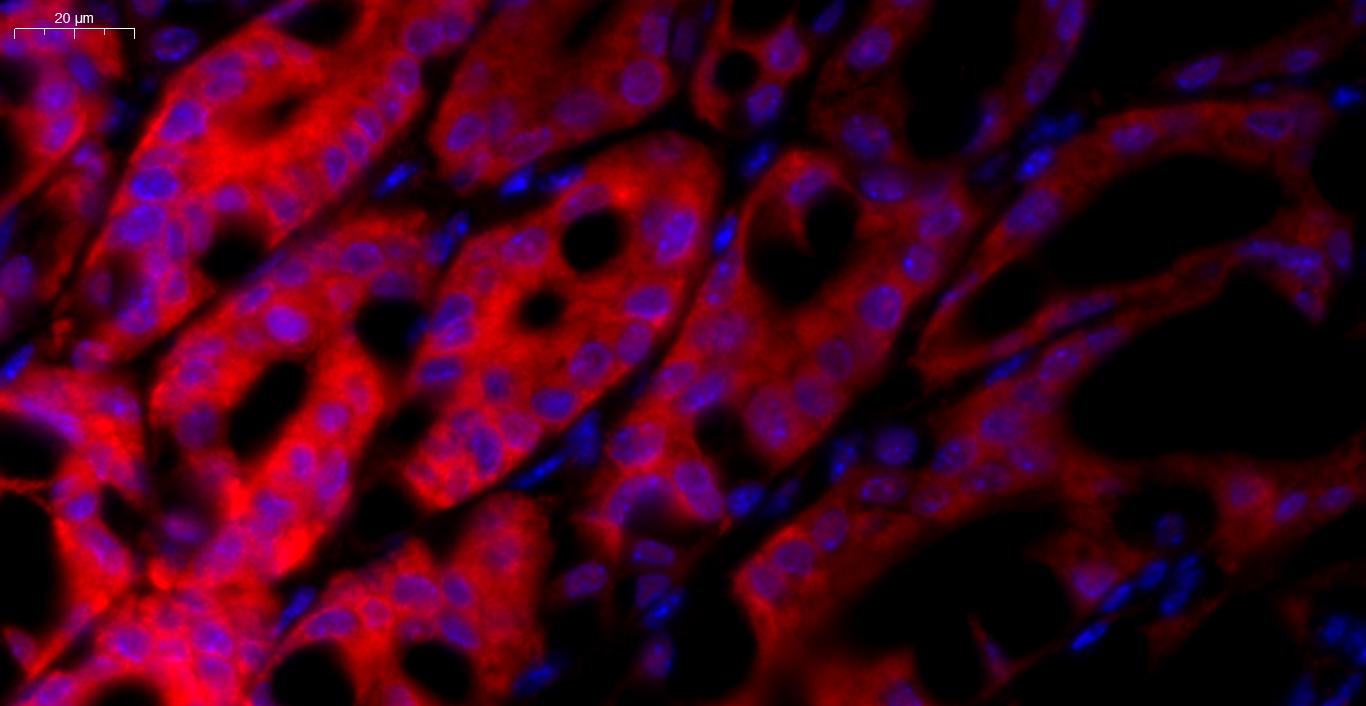

Supplement: Supplemental Information 6 — Immunofluorescence analysis of NF-kB [file peerj-10-14209-s006.zip › Fig. 6 raw data/Immunofluorescence analysis of NF-kB/The group of DSS/The Merge picture of DSS group.jpg]

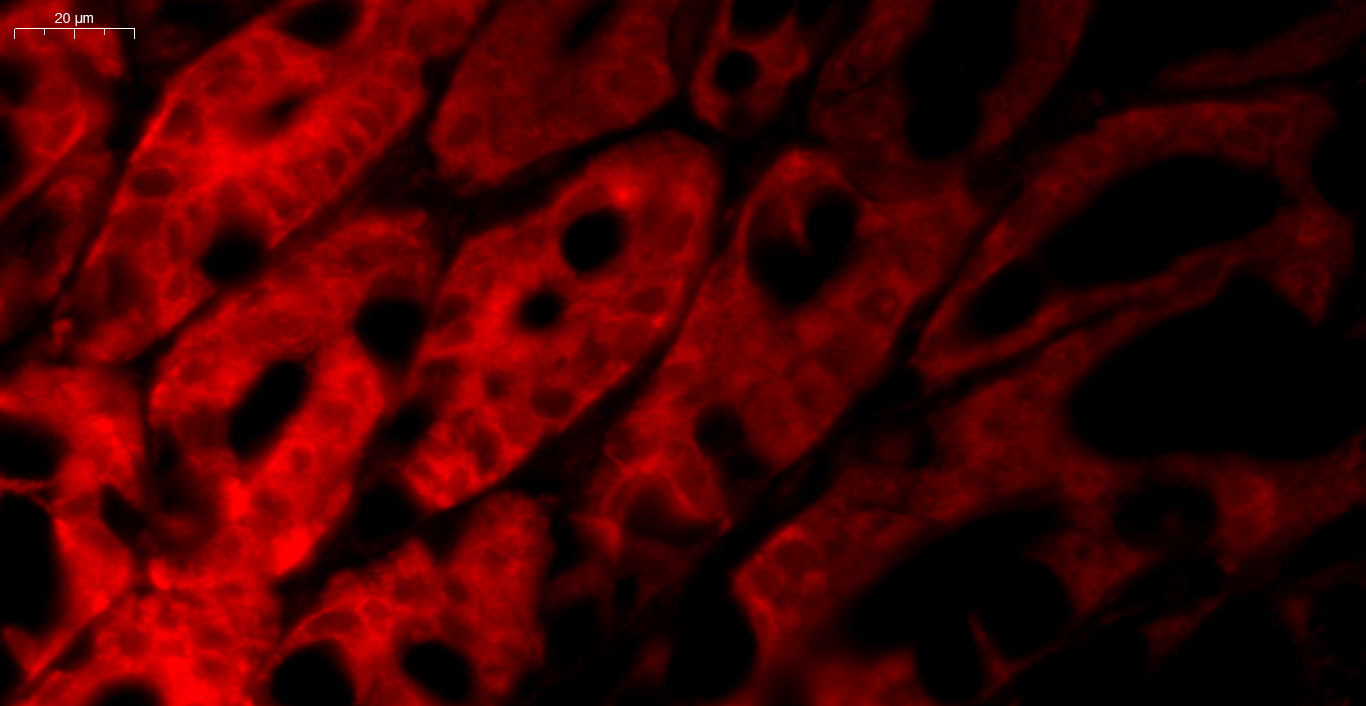

Supplement: Supplemental Information 6 — Immunofluorescence analysis of NF-kB [file peerj-10-14209-s006.zip › Fig. 6 raw data/Immunofluorescence analysis of NF-kB/The group of DSS/The NF-kB picture of DSS group.jpg]

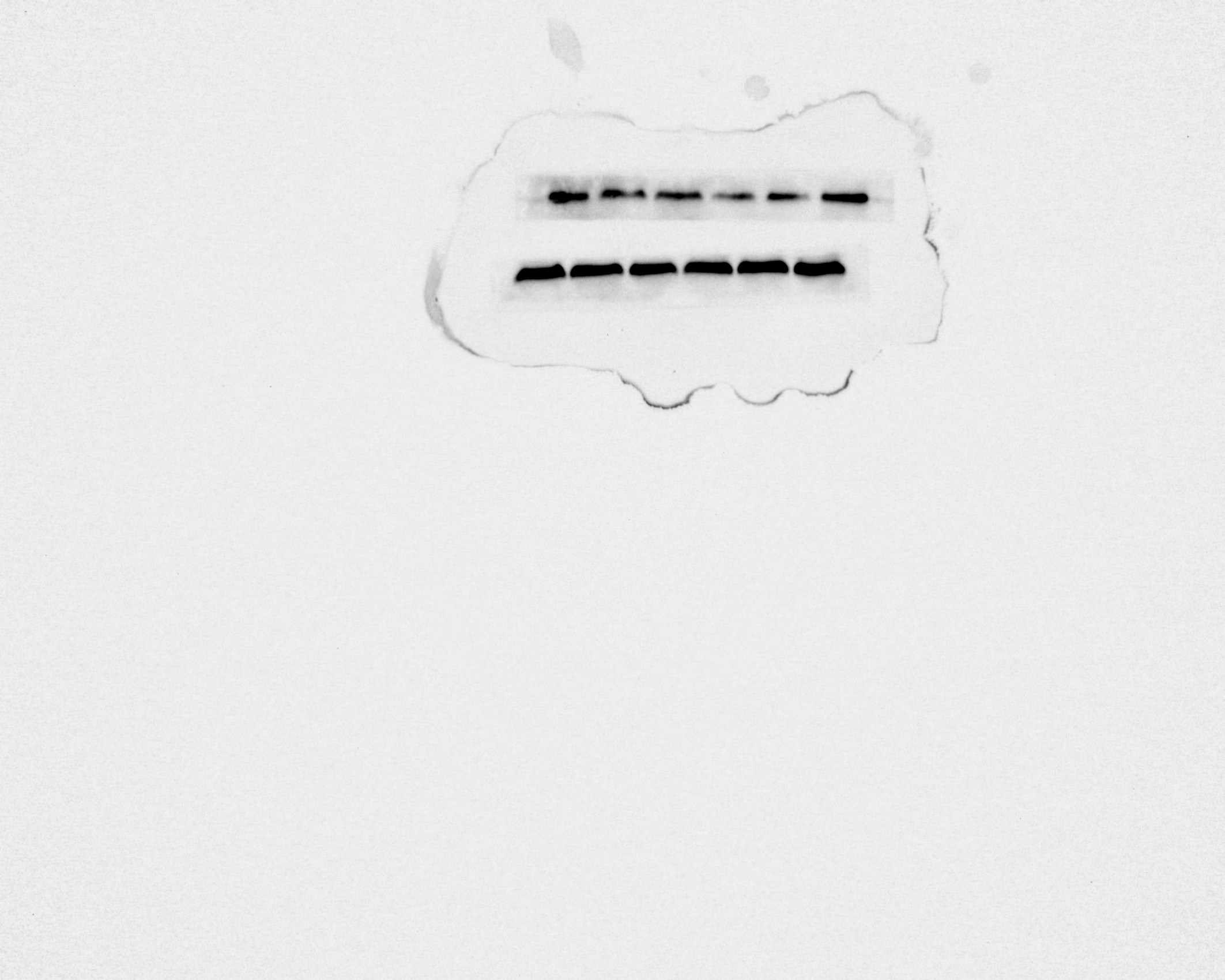

Supplement: Supplemental Information 7 [file peerj-10-14209-s007.zip › Fig. 7 raw data/Figure 7A original Western Blot images/Figure7A,9D,10D a┬-actin.tif]

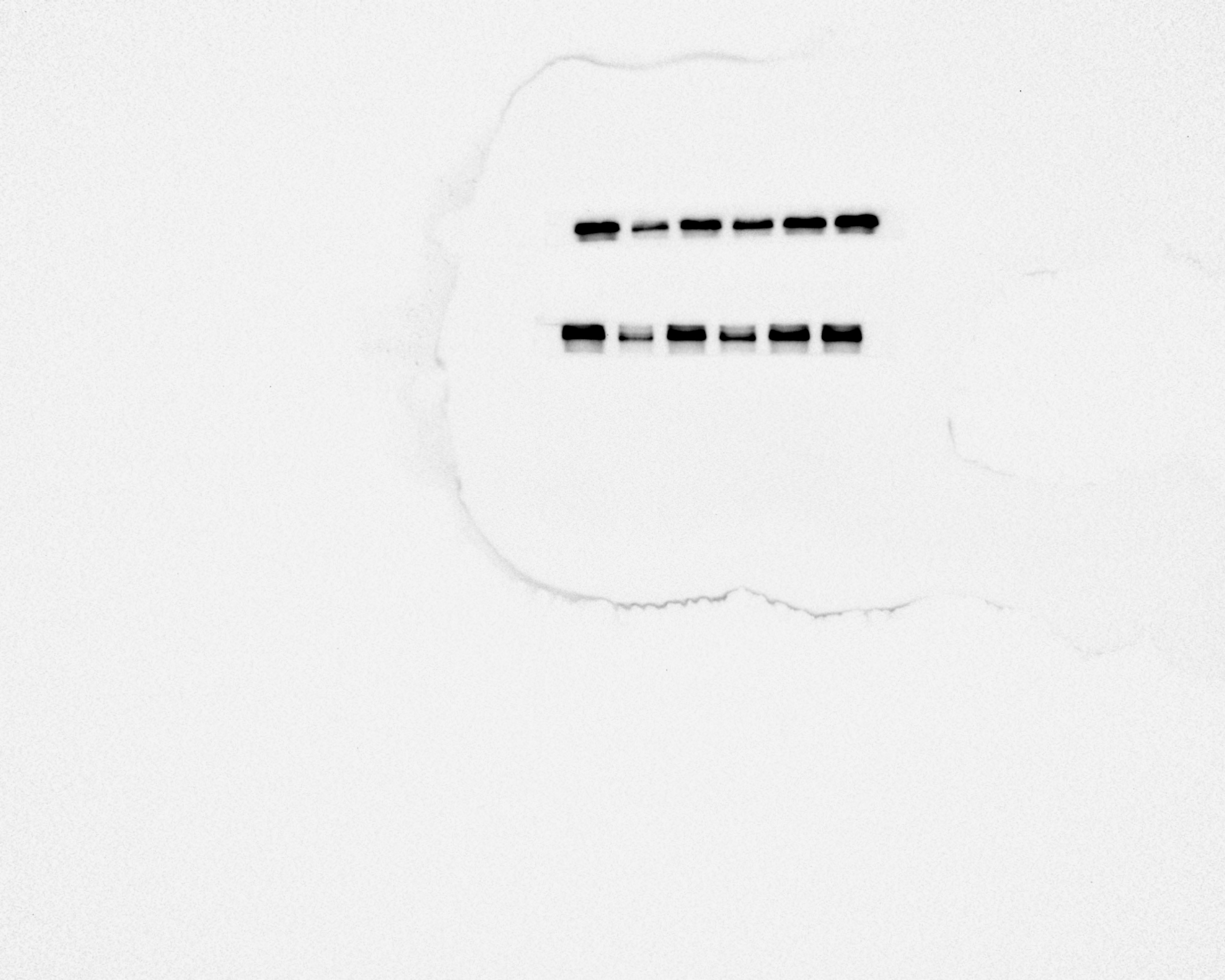

Supplement: Supplemental Information 7 [file peerj-10-14209-s007.zip › Fig. 7 raw data/Figure 7A original Western Blot images/IKB-a┴ First Blot from the top down.jpg]

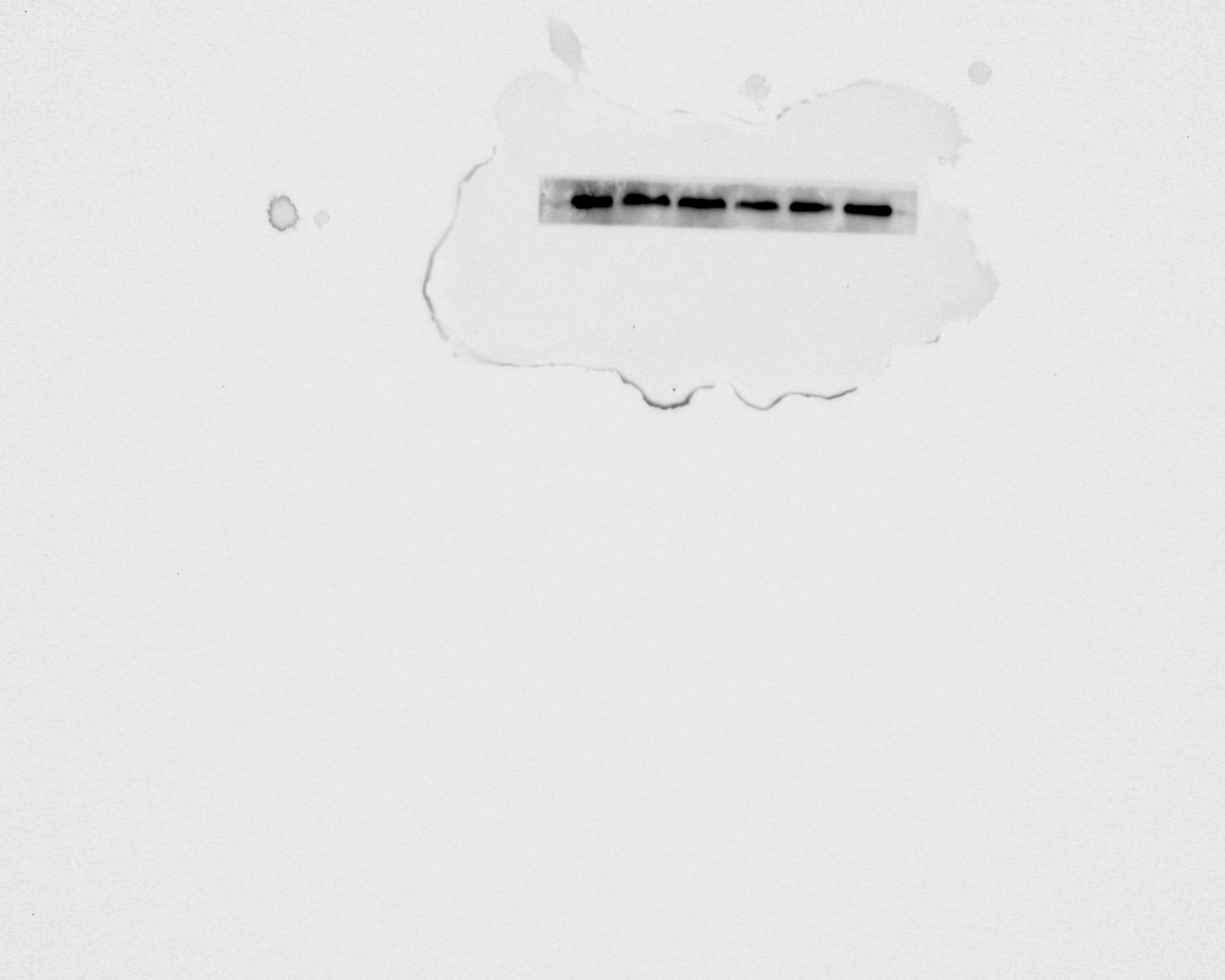

Supplement: Supplemental Information 7 [file peerj-10-14209-s007.zip › Fig. 7 raw data/Figure 7A original Western Blot images/IKK-a┴a┬.tif]

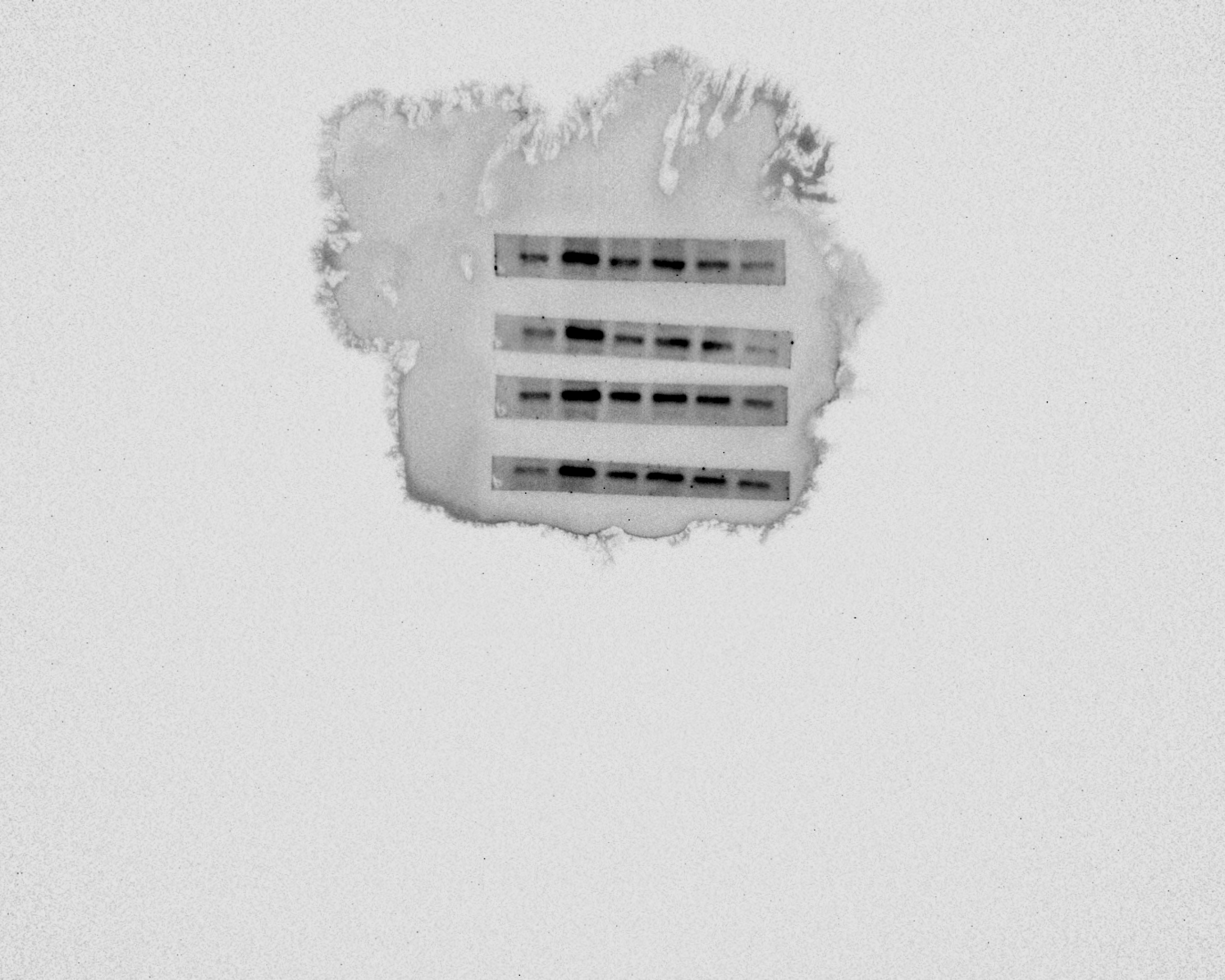

Supplement: Supplemental Information 7 [file peerj-10-14209-s007.zip › Fig. 7 raw data/Figure 7A original Western Blot images/P65.tif]

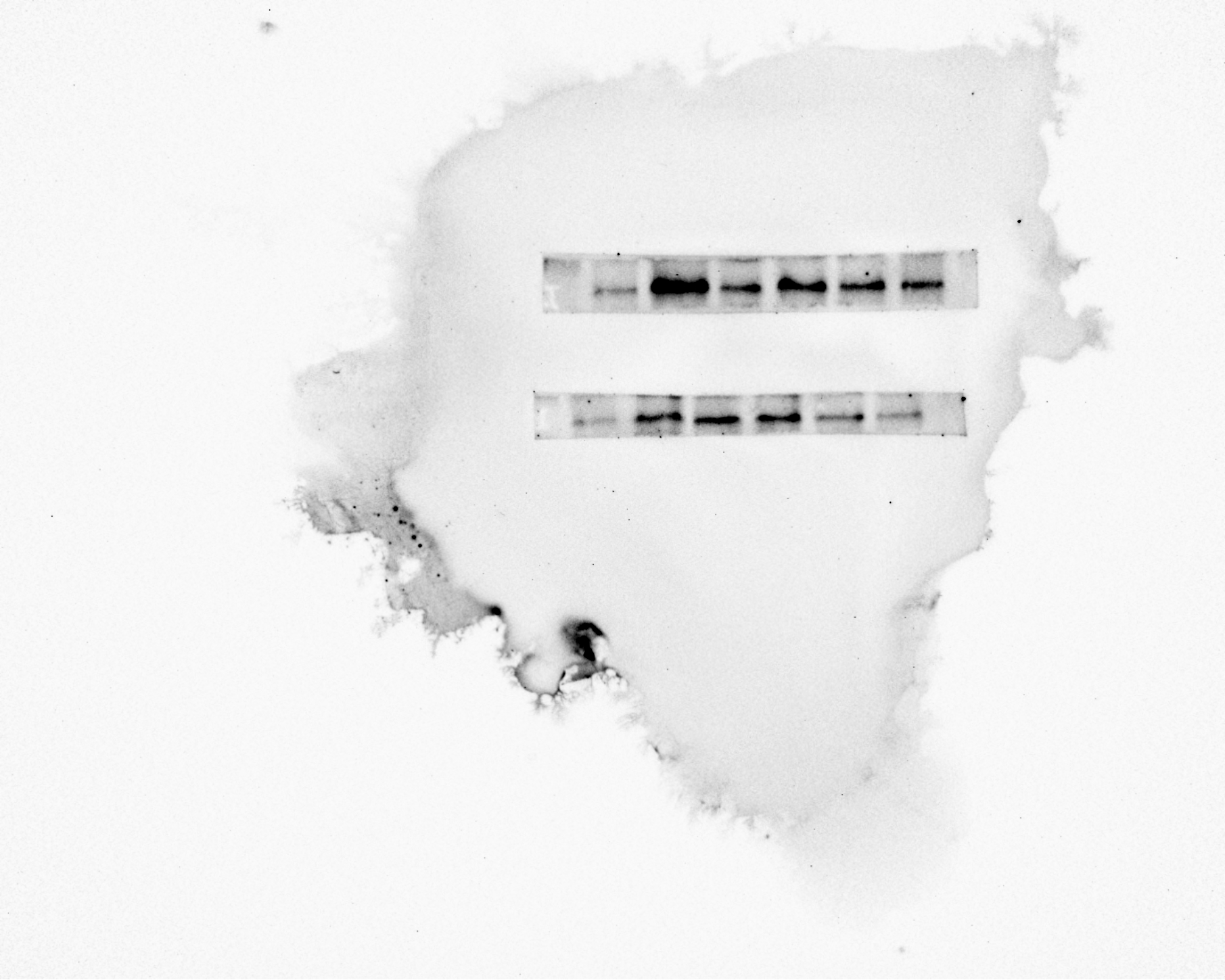

Supplement: Supplemental Information 7 [file peerj-10-14209-s007.zip › Fig. 7 raw data/Figure 7A original Western Blot images/p-IKBa┴ First Blot from the top down.jpg]

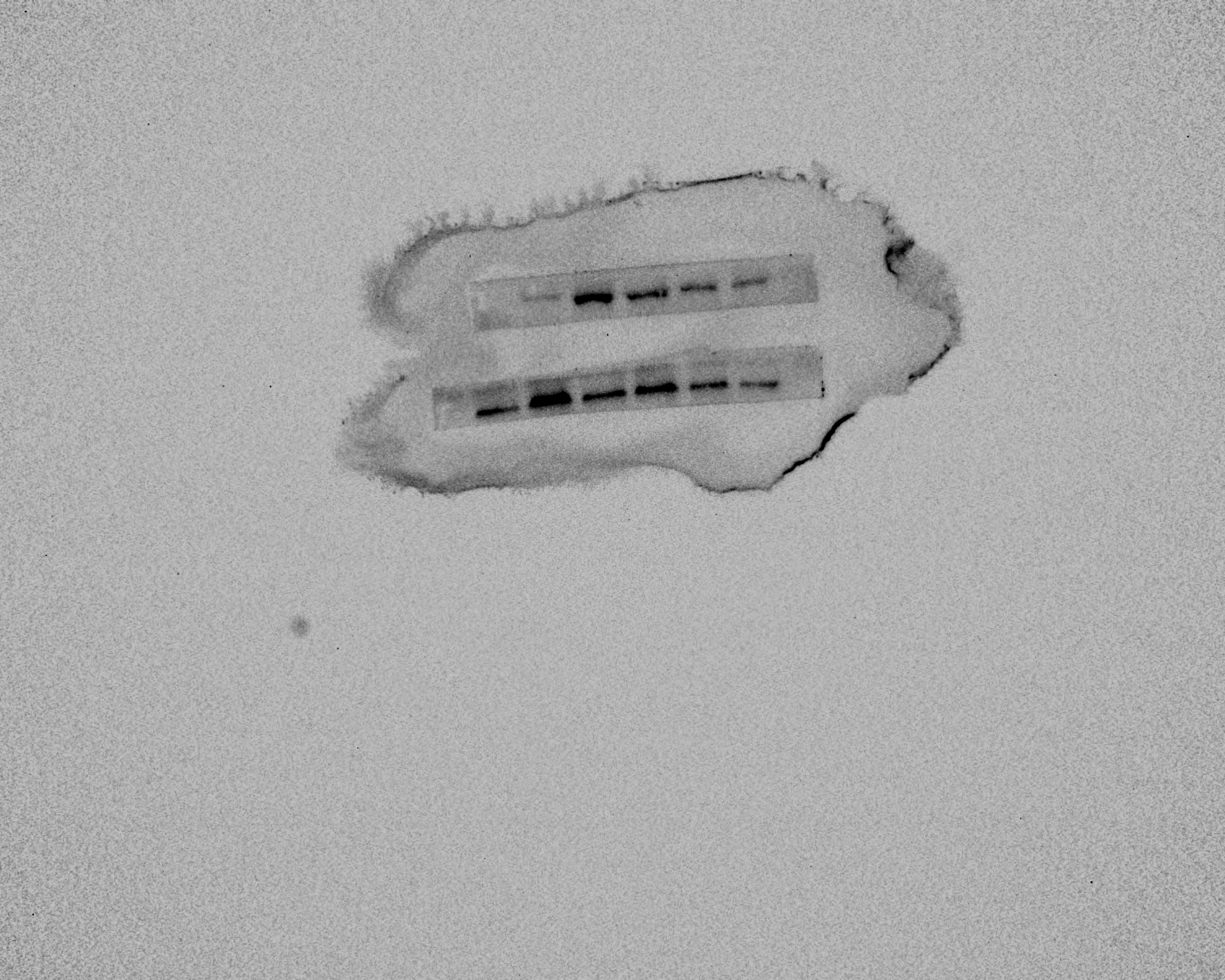

Supplement: Supplemental Information 7 [file peerj-10-14209-s007.zip › Fig. 7 raw data/Figure 7A original Western Blot images/p-IKKa┴ a┬ First Blot from the top down.jpg]

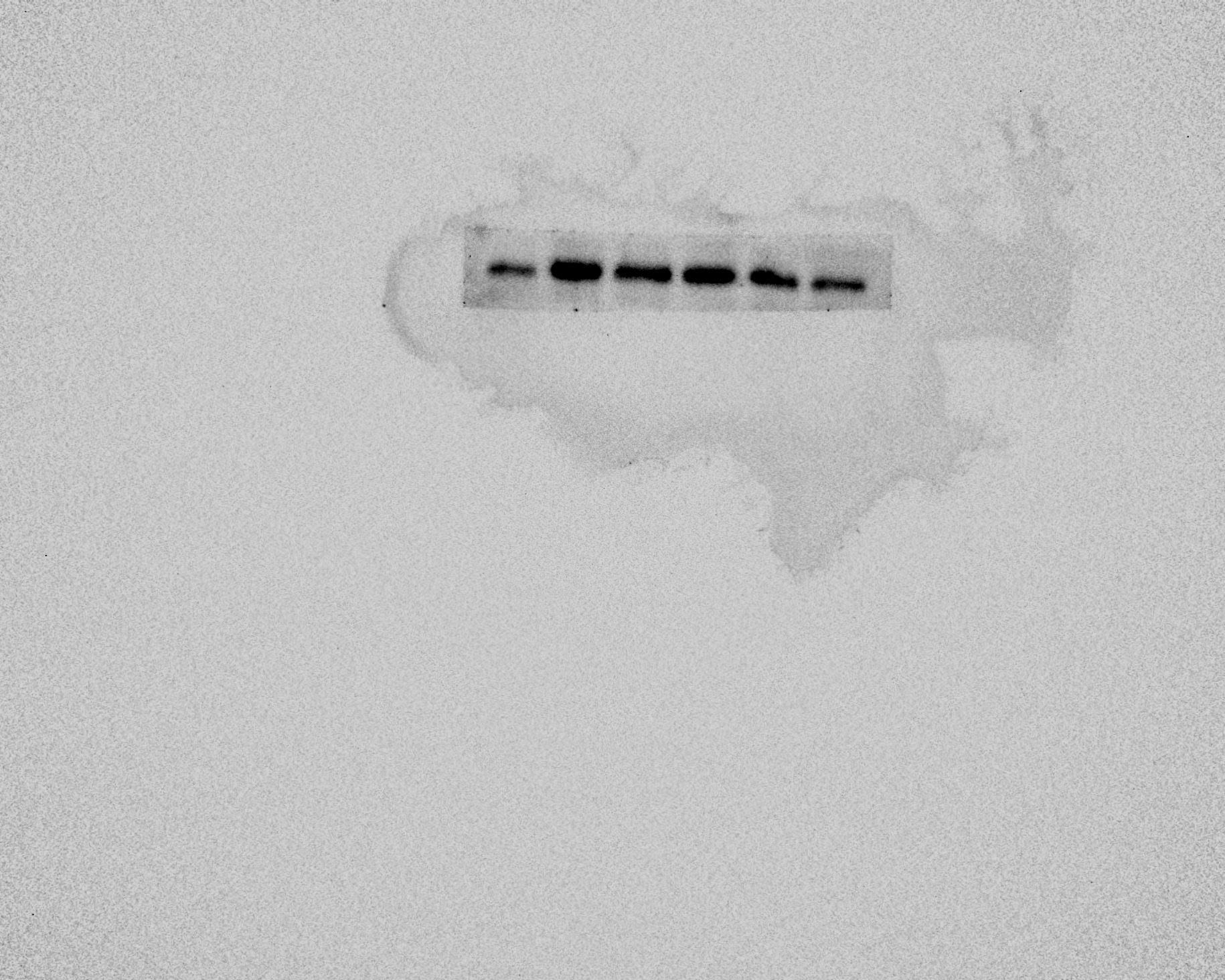

Supplement: Supplemental Information 7 [file peerj-10-14209-s007.zip › Fig. 7 raw data/Figure 7A original Western Blot images/p-P65.jpg]

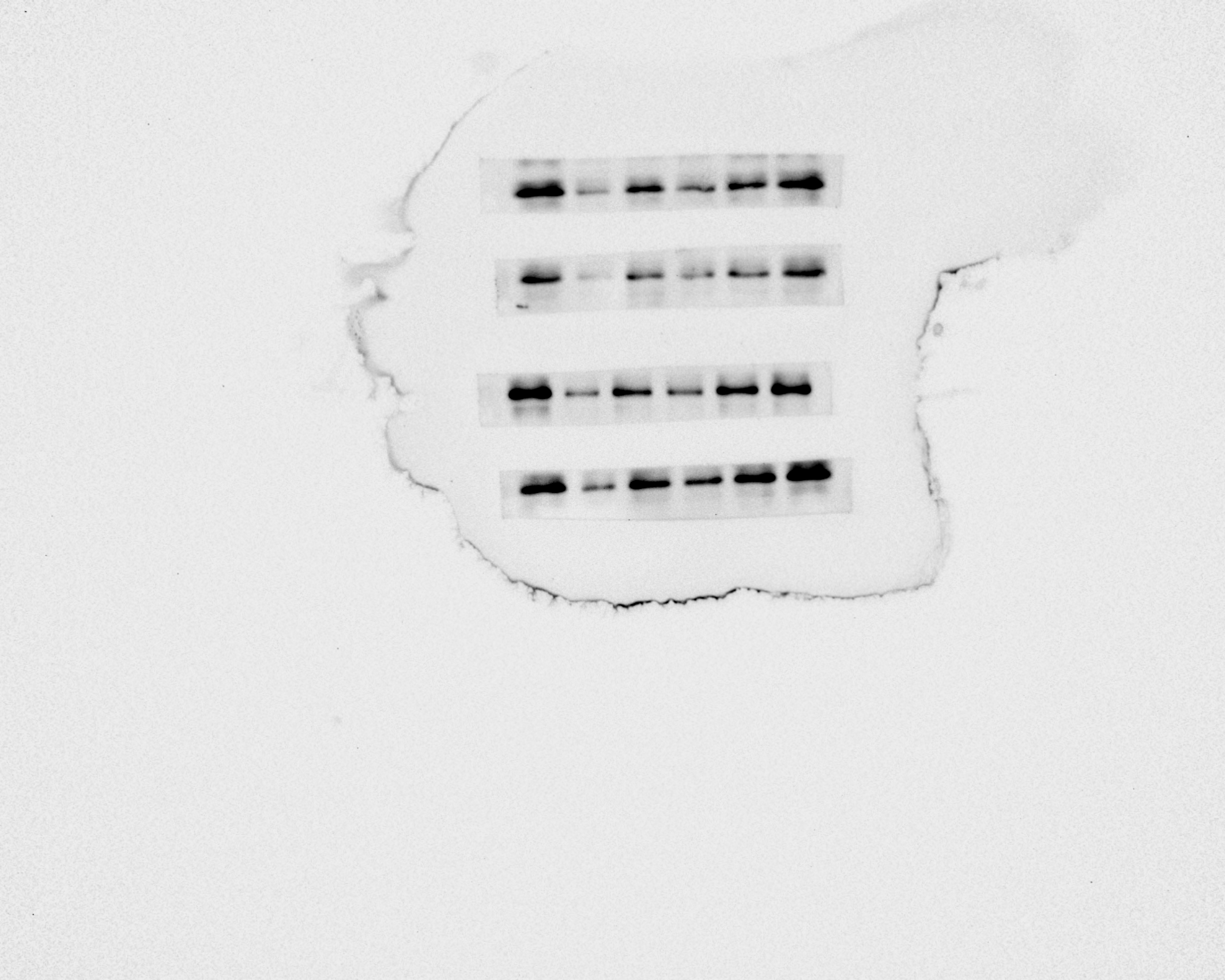

Supplement: Supplemental Information 8 — Immunofluorescence analysis of Nrf-2 [file peerj-10-14209-s008.zip › Fig. 8 raw data/Figure 8A original Western Blot images/HO-1 Third Blot from the top down.jpg]

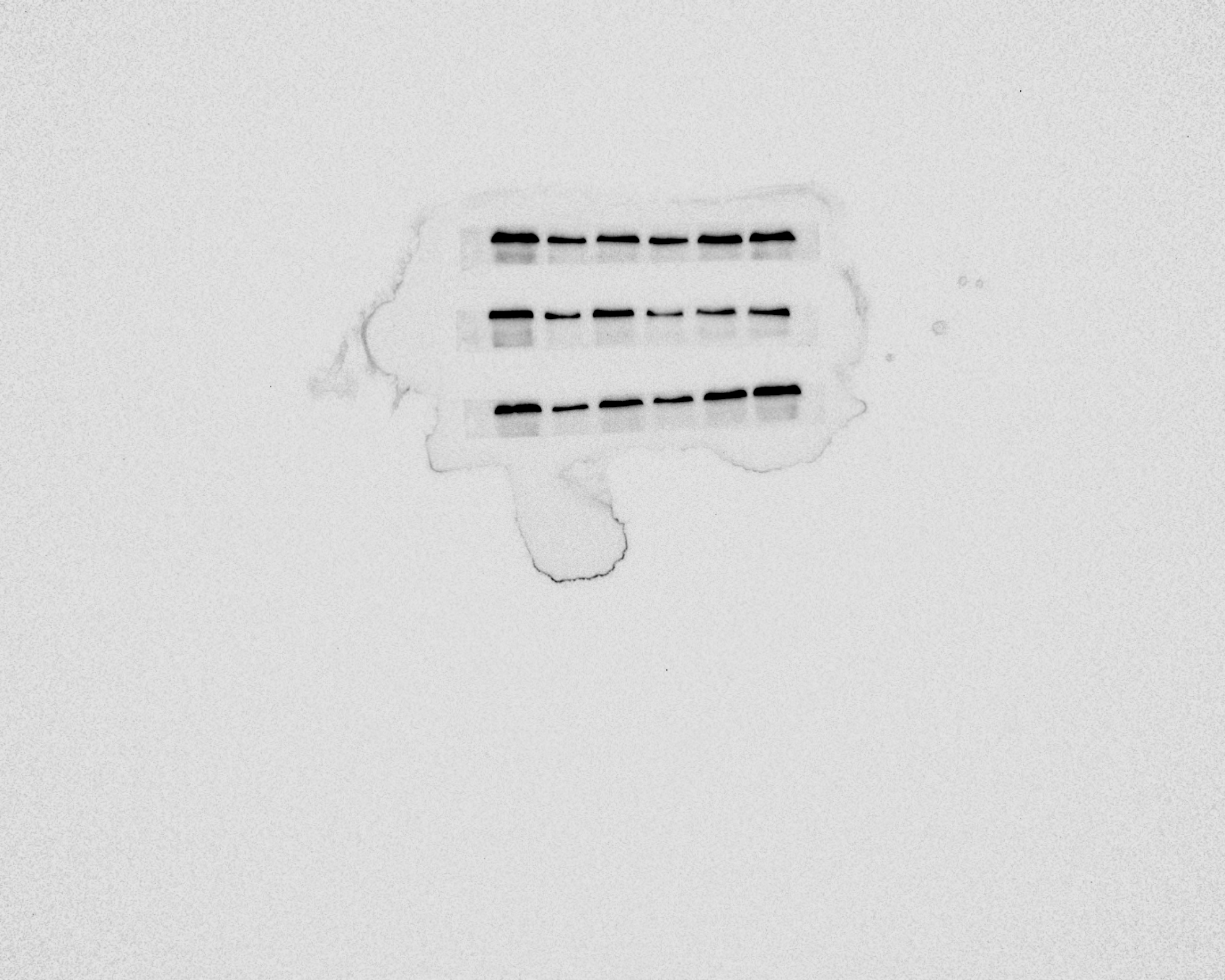

Supplement: Supplemental Information 8 — Immunofluorescence analysis of Nrf-2 [file peerj-10-14209-s008.zip › Fig. 8 raw data/Figure 8A original Western Blot images/Keap-1 Third Blot from the top down.jpg]

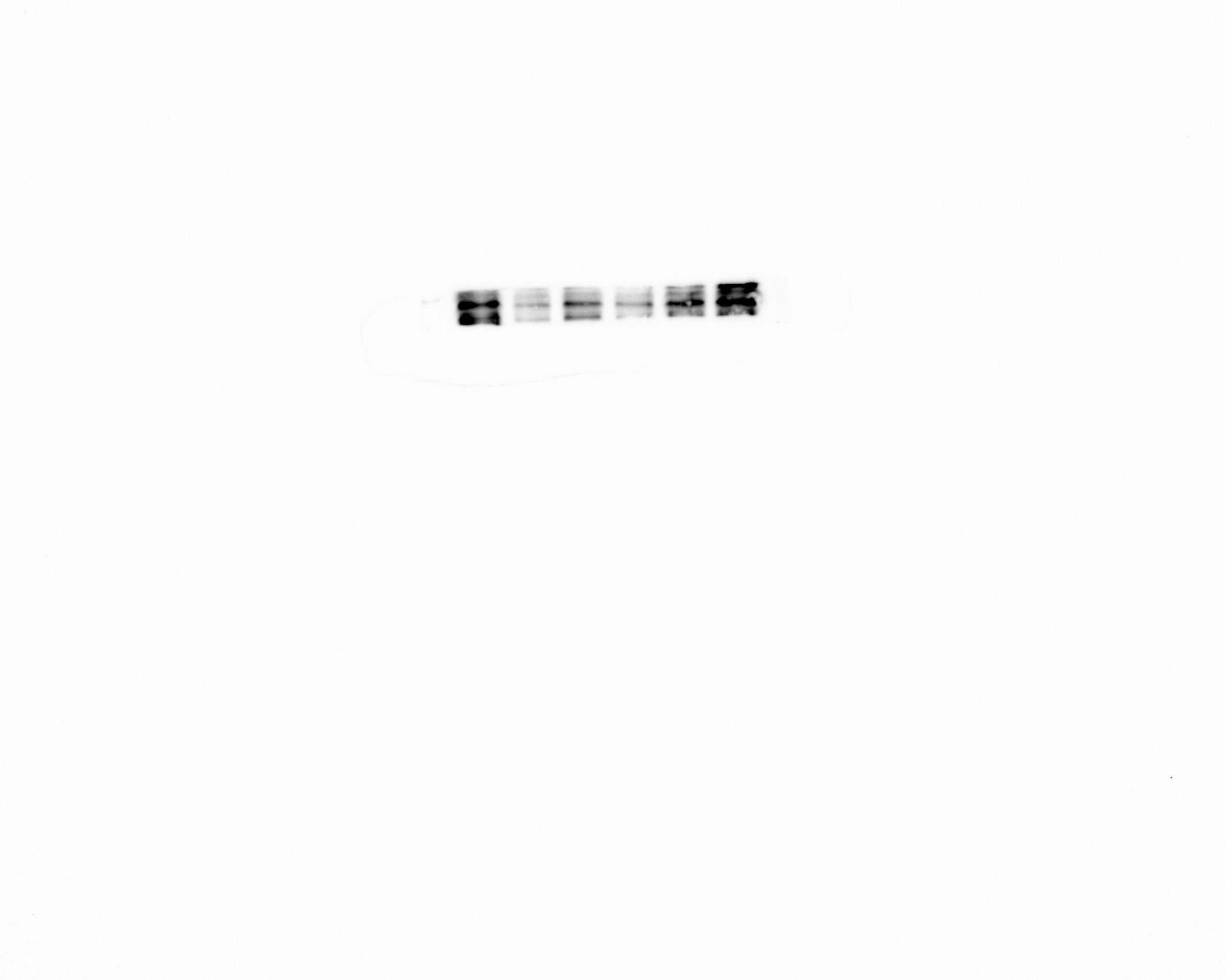

Supplement: Supplemental Information 8 — Immunofluorescence analysis of Nrf-2 [file peerj-10-14209-s008.zip › Fig. 8 raw data/Figure 8A original Western Blot images/Nrf-2.jpg]

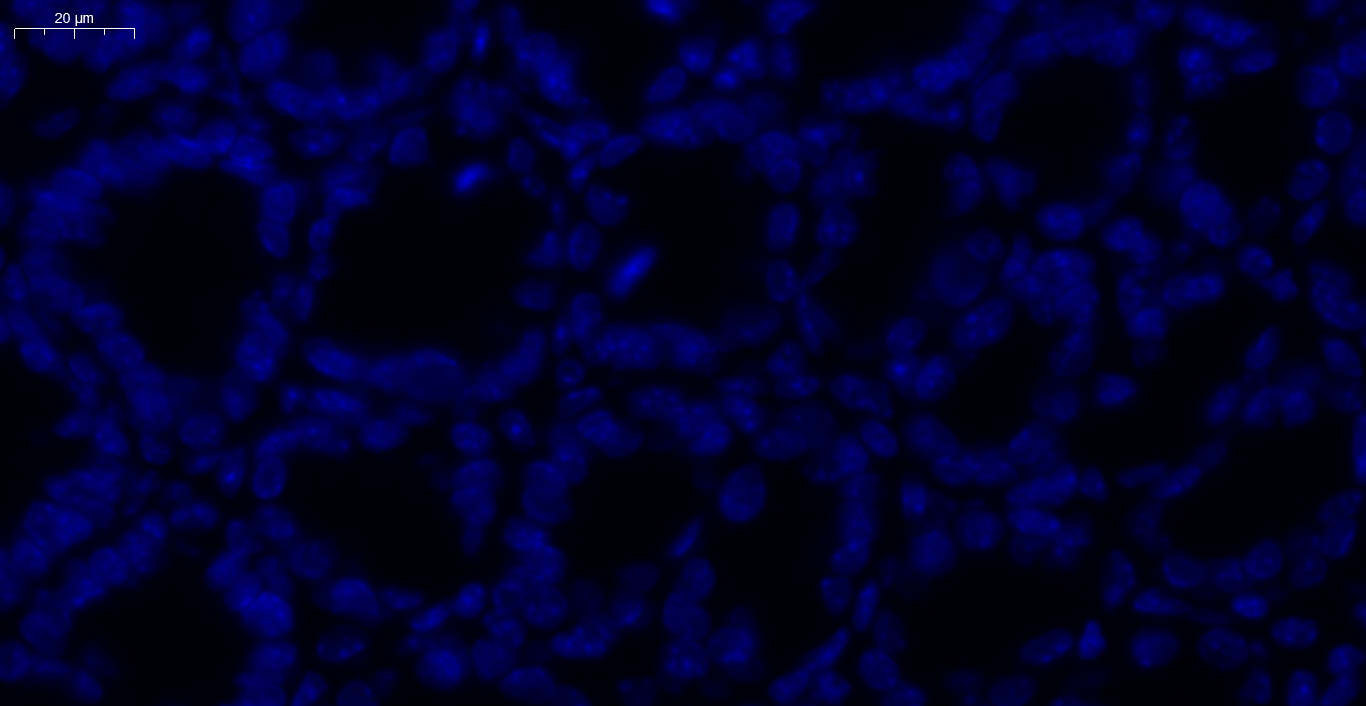

Supplement: Supplemental Information 8 — Immunofluorescence analysis of Nrf-2 [file peerj-10-14209-s008.zip › Fig. 8 raw data/Immunofluorescence analysis of Nrf-2/The group of control/The DAPI picture of control group.jpg]

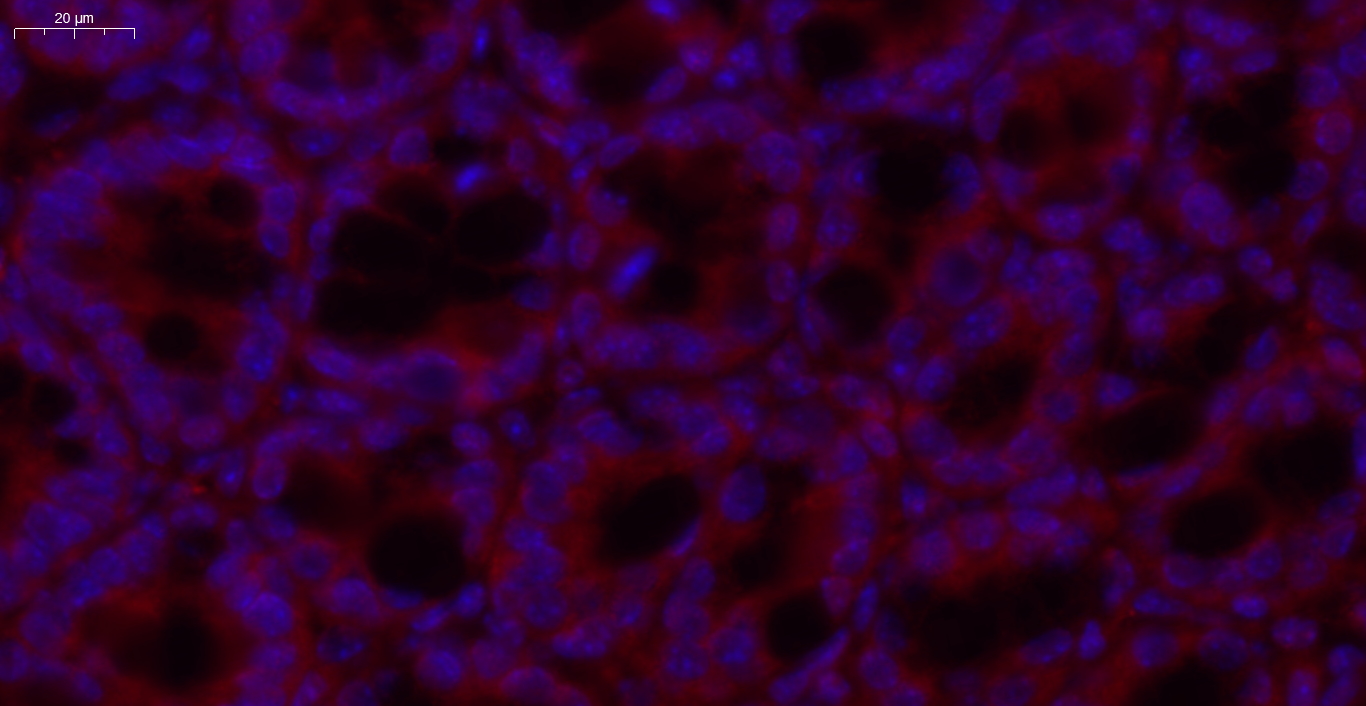

Supplement: Supplemental Information 8 — Immunofluorescence analysis of Nrf-2 [file peerj-10-14209-s008.zip › Fig. 8 raw data/Immunofluorescence analysis of Nrf-2/The group of control/The Merge picture of control group.jpg]

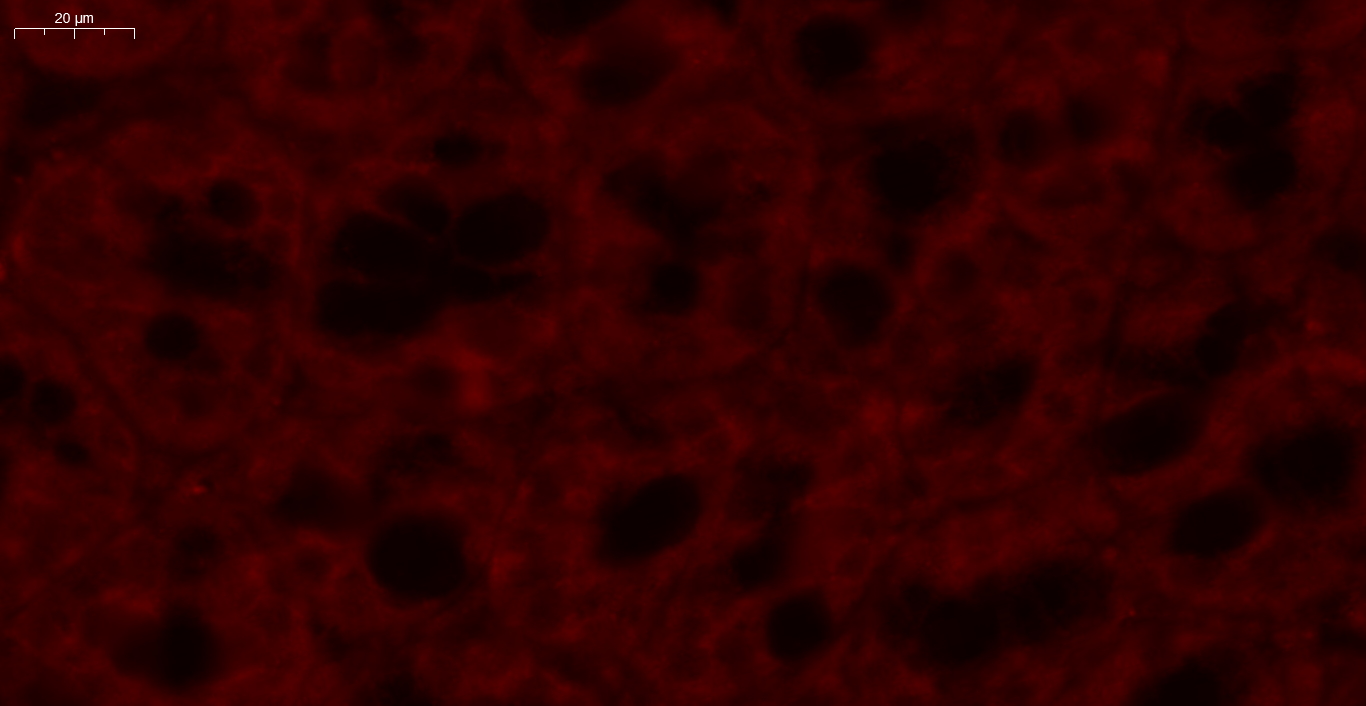

Supplement: Supplemental Information 8 — Immunofluorescence analysis of Nrf-2 [file peerj-10-14209-s008.zip › Fig. 8 raw data/Immunofluorescence analysis of Nrf-2/The group of control/The Nrf-2 picture of control group.jpg]

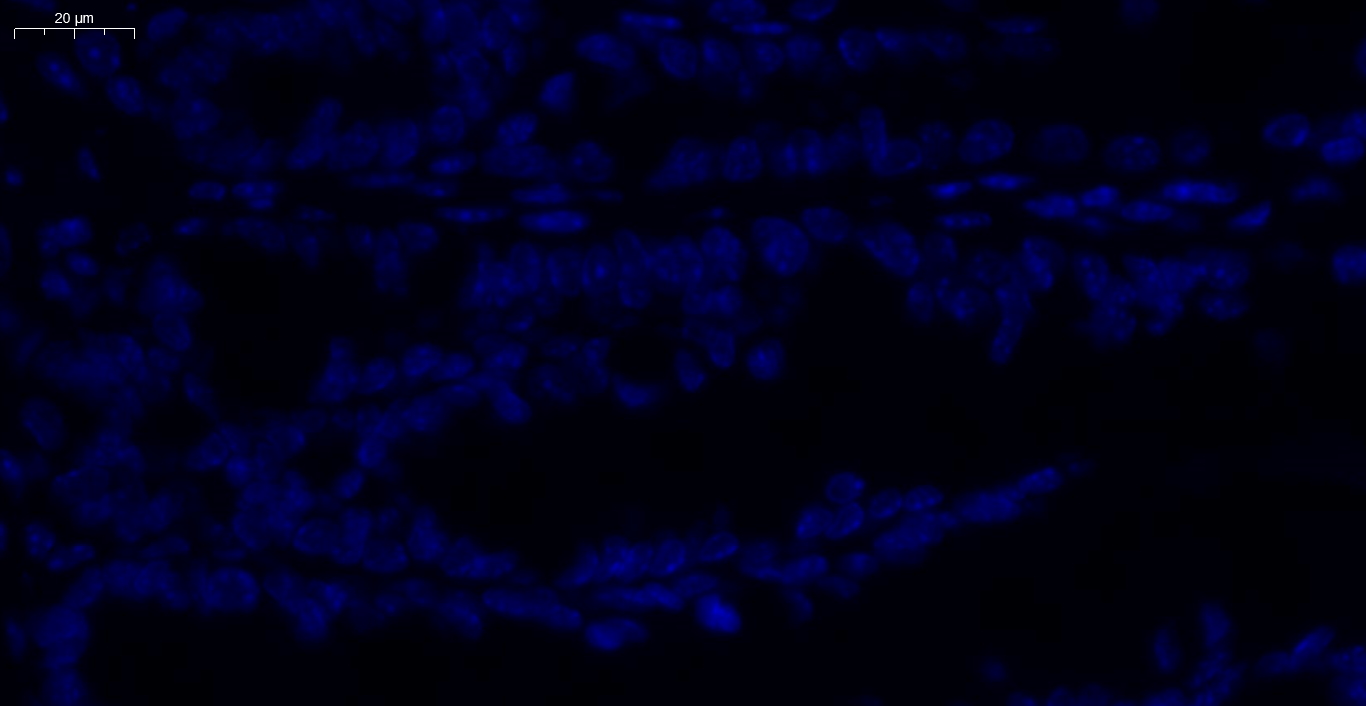

Supplement: Supplemental Information 8 — Immunofluorescence analysis of Nrf-2 [file peerj-10-14209-s008.zip › Fig. 8 raw data/Immunofluorescence analysis of Nrf-2/The group of 100mg kg MN/The DAPI picture of 100 mg kg MN group.jpg]

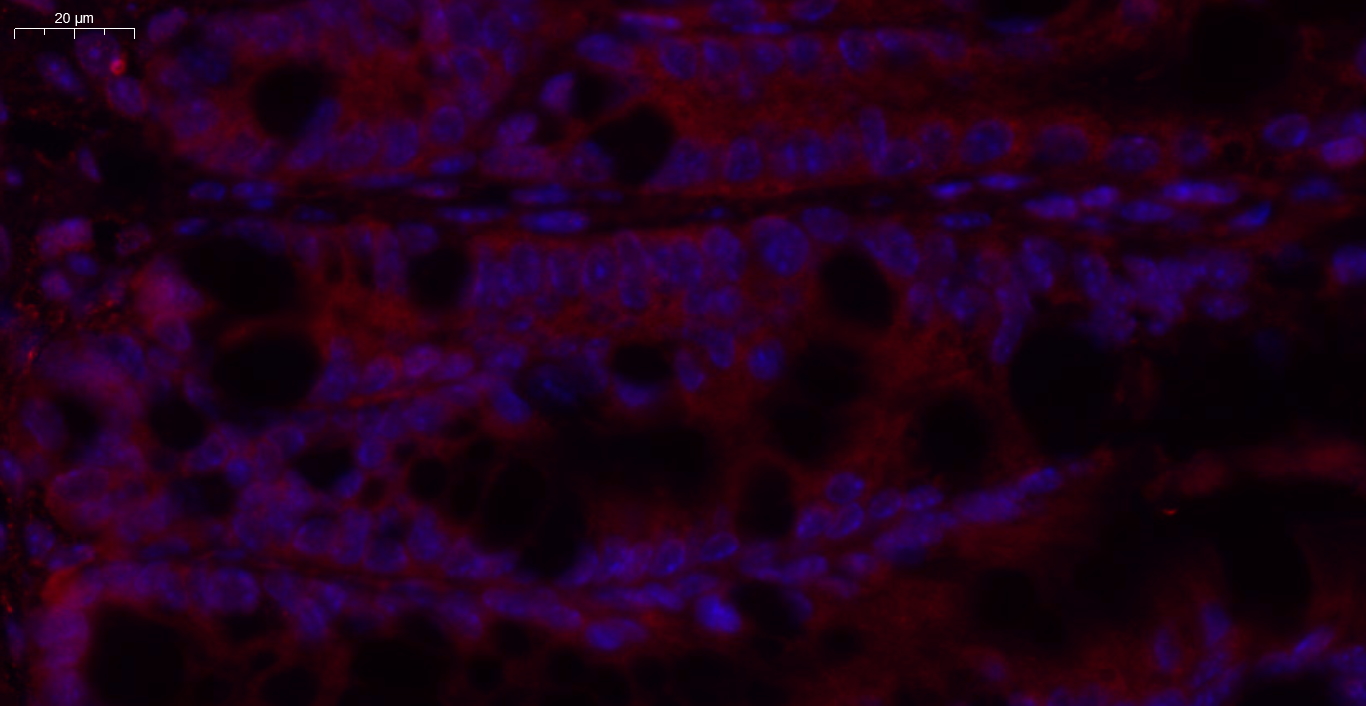

Supplement: Supplemental Information 8 — Immunofluorescence analysis of Nrf-2 [file peerj-10-14209-s008.zip › Fig. 8 raw data/Immunofluorescence analysis of Nrf-2/The group of 100mg kg MN/The Merge picture of 100 mg kg MN group.jpg]

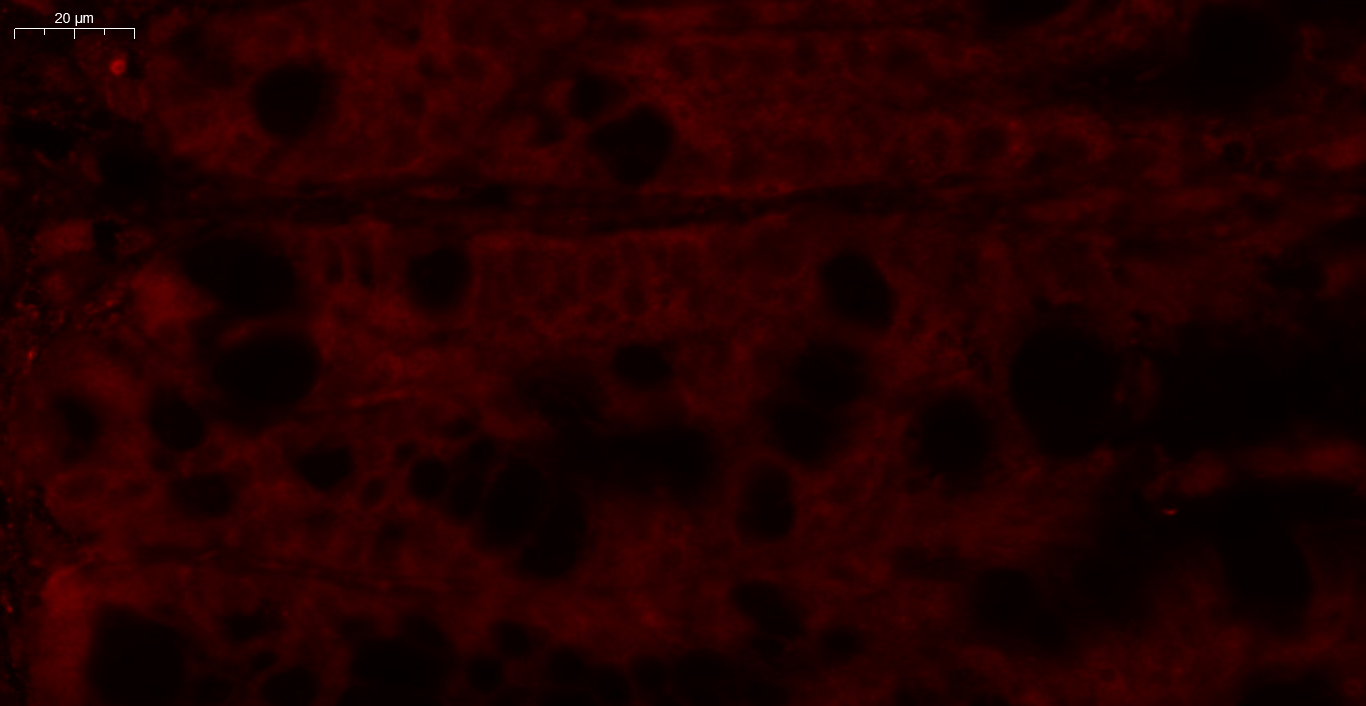

Supplement: Supplemental Information 8 — Immunofluorescence analysis of Nrf-2 [file peerj-10-14209-s008.zip › Fig. 8 raw data/Immunofluorescence analysis of Nrf-2/The group of 100mg kg MN/The Nrf-2 picture of 100 mg kg MN group.jpg]

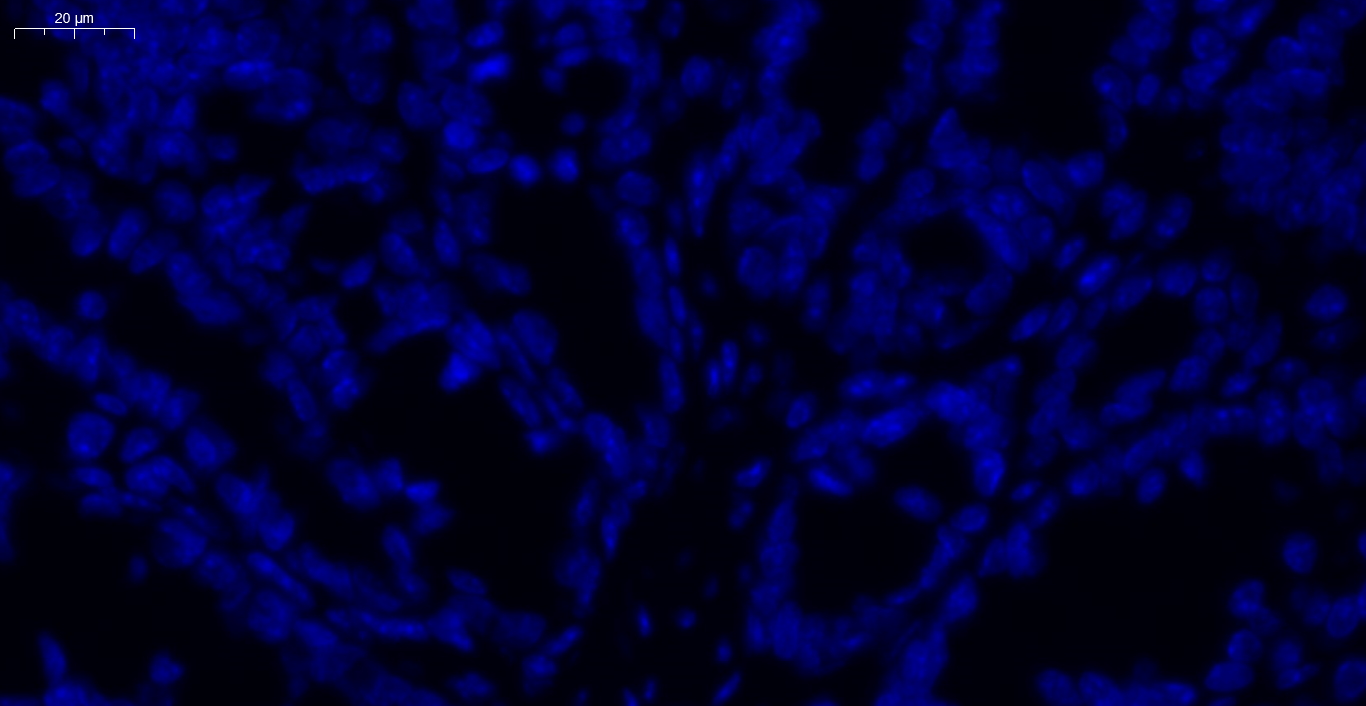

Supplement: Supplemental Information 8 — Immunofluorescence analysis of Nrf-2 [file peerj-10-14209-s008.zip › Fig. 8 raw data/Immunofluorescence analysis of Nrf-2/The group of 200mg kg MN -/The DAPI picture of 200mg kg MN group.jpg]

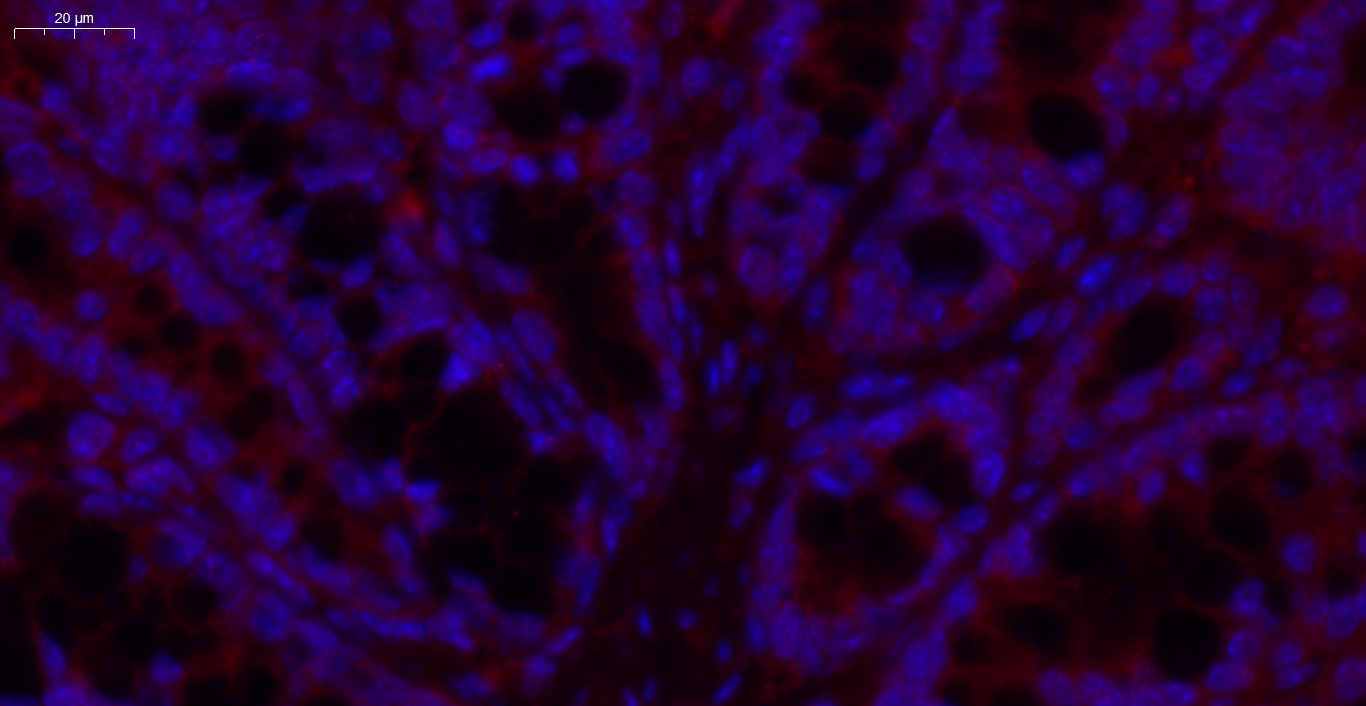

Supplement: Supplemental Information 8 — Immunofluorescence analysis of Nrf-2 [file peerj-10-14209-s008.zip › Fig. 8 raw data/Immunofluorescence analysis of Nrf-2/The group of 200mg kg MN -/The Merge picture of 200mg kg MN group.jpg]

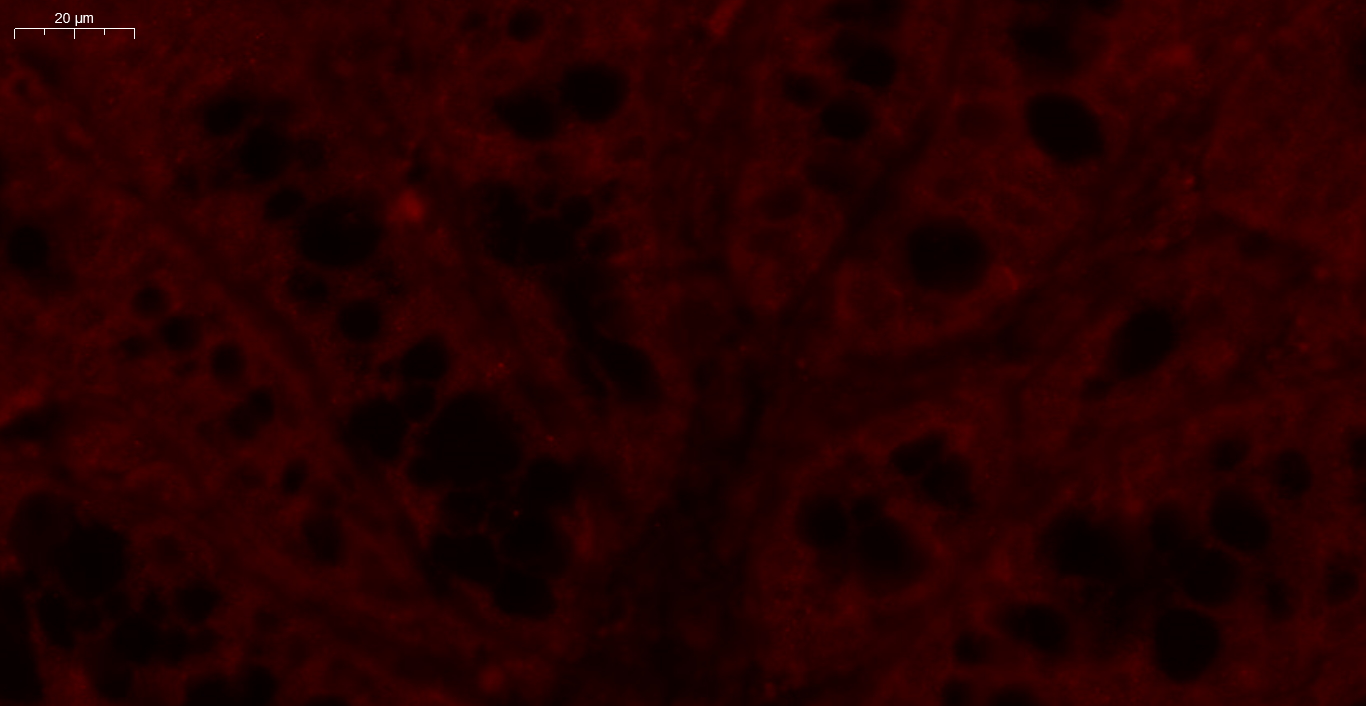

Supplement: Supplemental Information 8 — Immunofluorescence analysis of Nrf-2 [file peerj-10-14209-s008.zip › Fig. 8 raw data/Immunofluorescence analysis of Nrf-2/The group of 200mg kg MN -/The Nrf-2 picture of 200mg kg MN group.jpg]

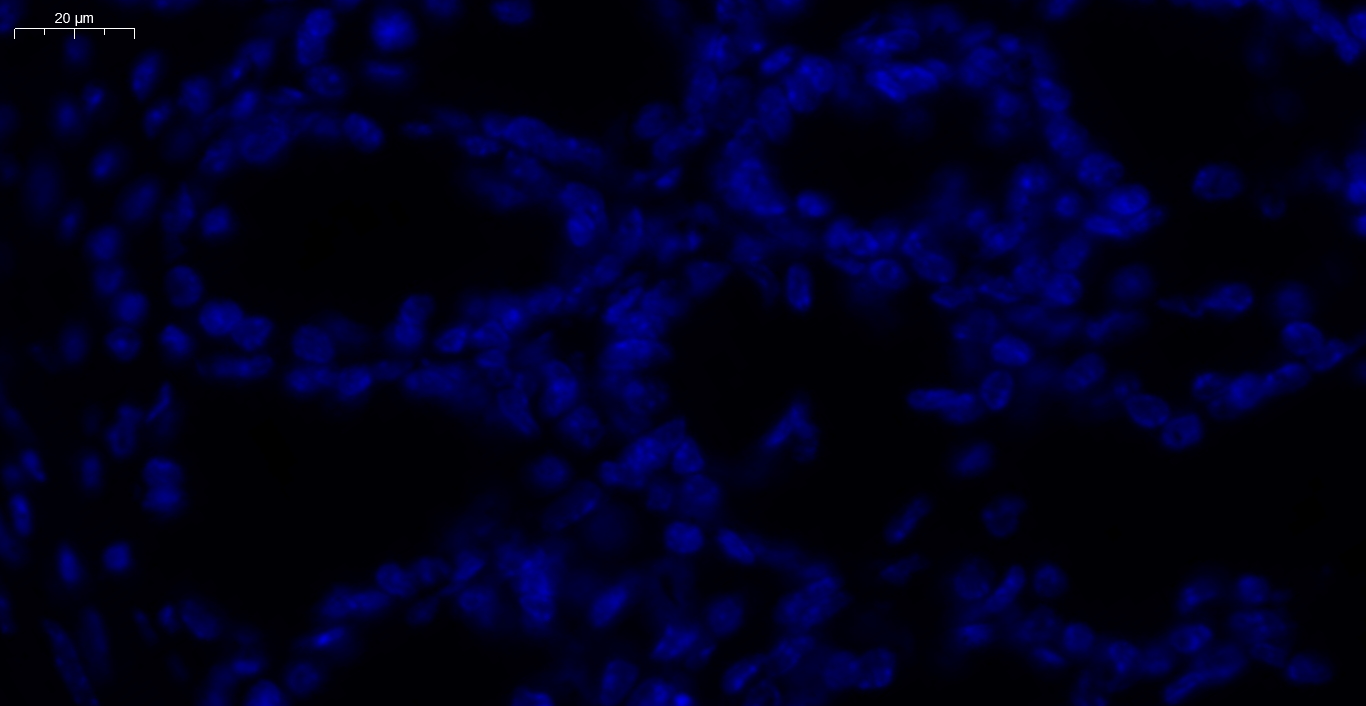

Supplement: Supplemental Information 8 — Immunofluorescence analysis of Nrf-2 [file peerj-10-14209-s008.zip › Fig. 8 raw data/Immunofluorescence analysis of Nrf-2/The group of 500mg kg 5-ASA/The DAPI picture of 500mg kg 5-ASA group.jpg]

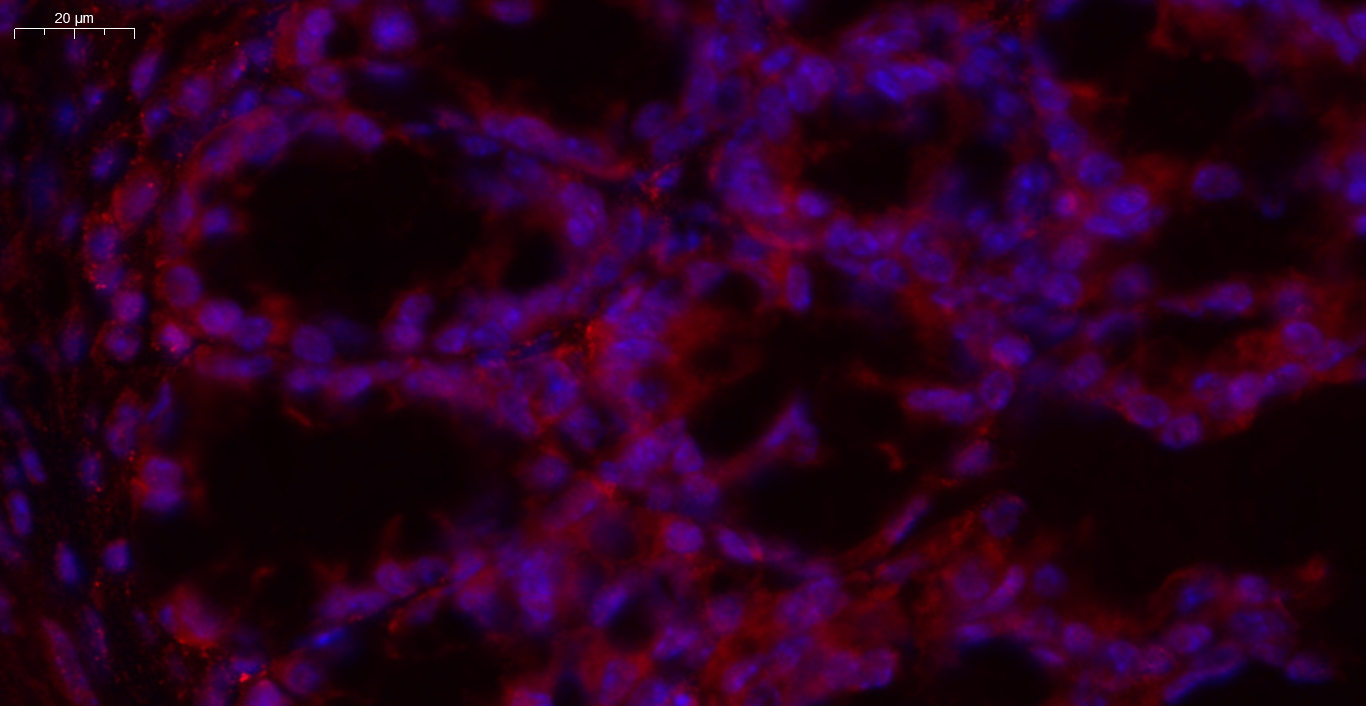

Supplement: Supplemental Information 8 — Immunofluorescence analysis of Nrf-2 [file peerj-10-14209-s008.zip › Fig. 8 raw data/Immunofluorescence analysis of Nrf-2/The group of 500mg kg 5-ASA/The Merge picture of 500mg kg 5-ASA group.jpg]

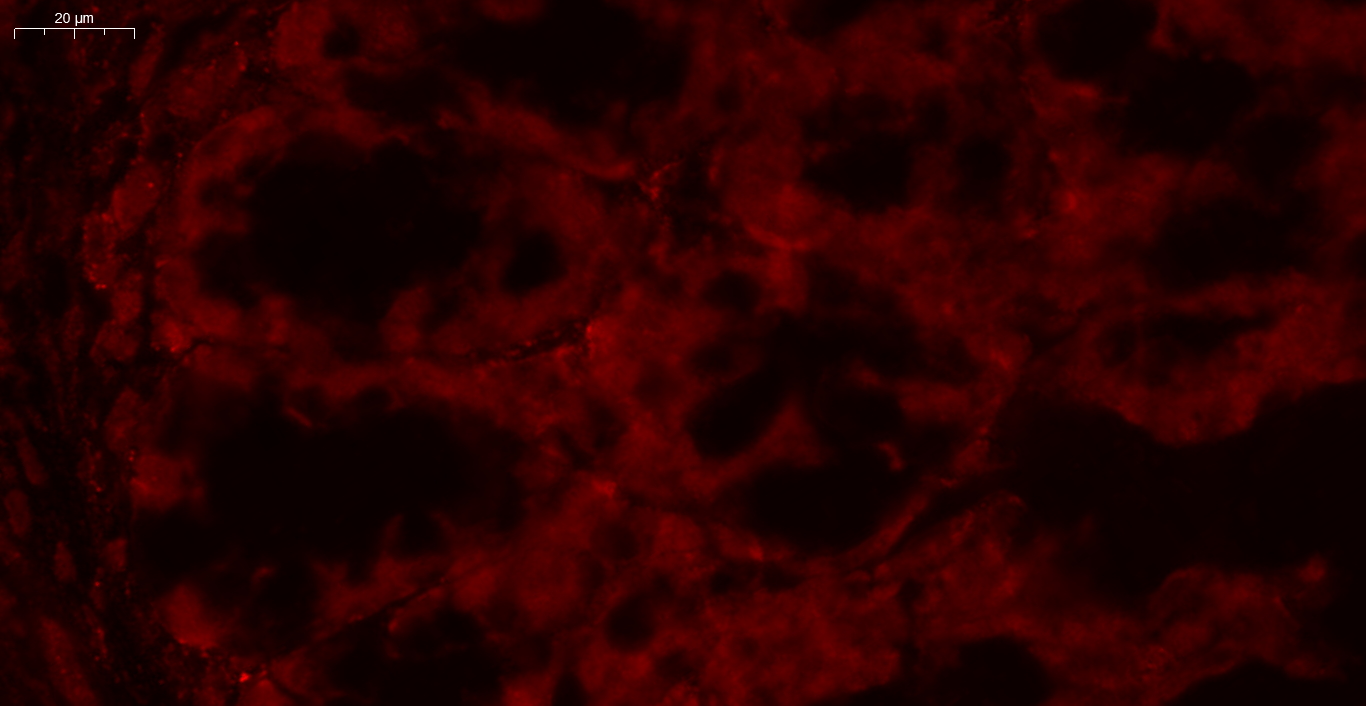

Supplement: Supplemental Information 8 — Immunofluorescence analysis of Nrf-2 [file peerj-10-14209-s008.zip › Fig. 8 raw data/Immunofluorescence analysis of Nrf-2/The group of 500mg kg 5-ASA/The Nrf-2 picture of 500mg kg 5-ASA group.jpg]

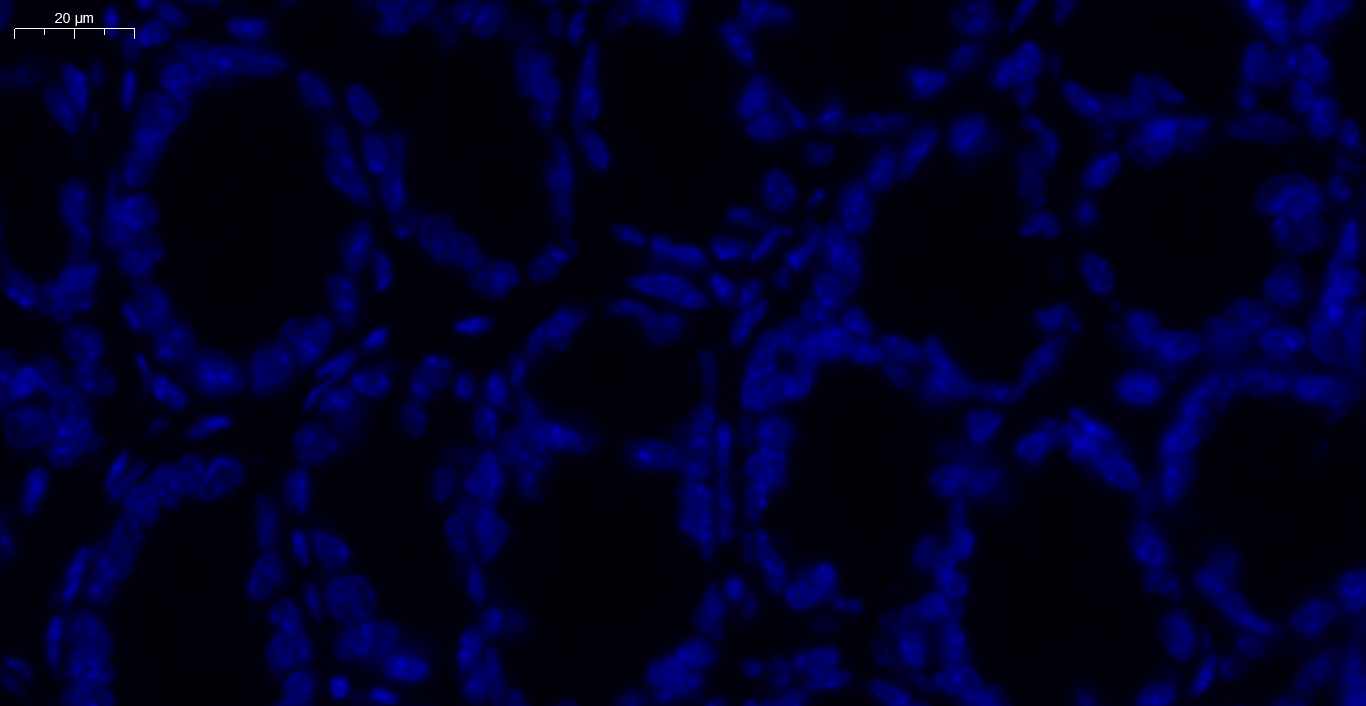

Supplement: Supplemental Information 8 — Immunofluorescence analysis of Nrf-2 [file peerj-10-14209-s008.zip › Fig. 8 raw data/Immunofluorescence analysis of Nrf-2/The group of 50mg kg MN/The DAPI picture of 50 mg kg MN group.jpg]

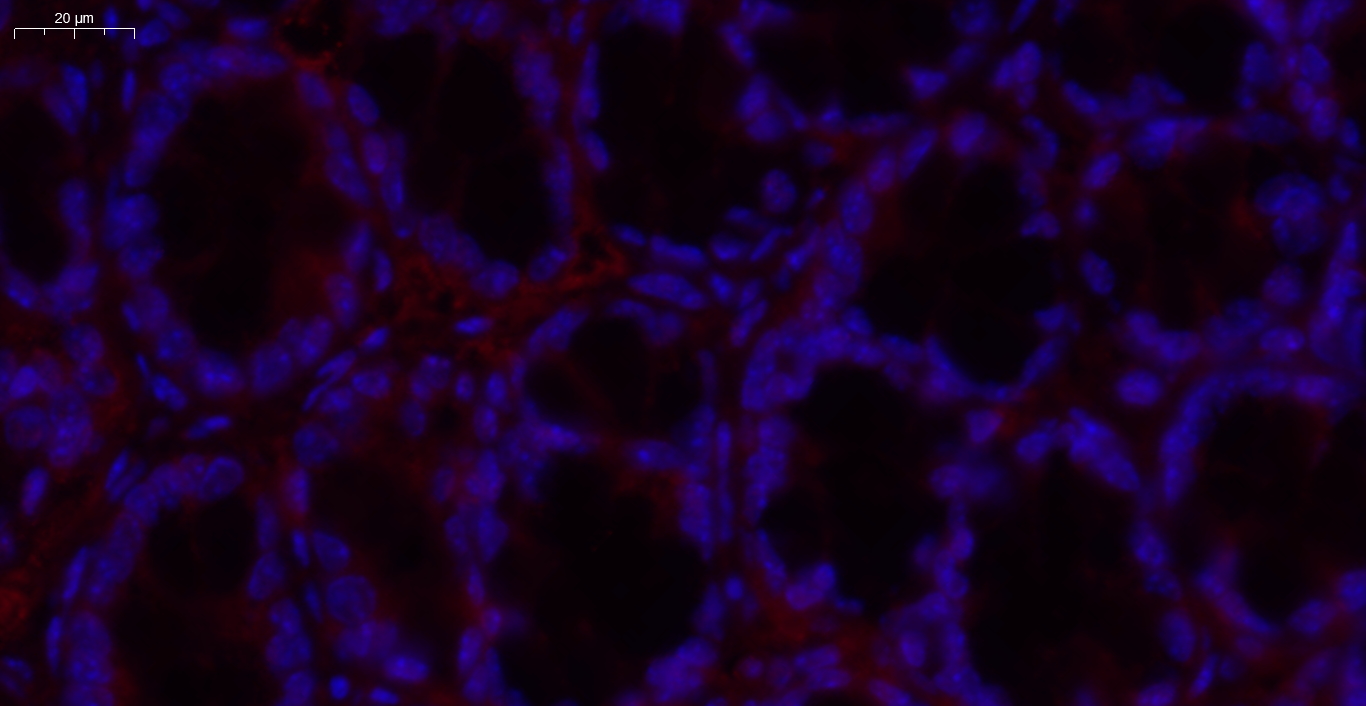

Supplement: Supplemental Information 8 — Immunofluorescence analysis of Nrf-2 [file peerj-10-14209-s008.zip › Fig. 8 raw data/Immunofluorescence analysis of Nrf-2/The group of 50mg kg MN/The Merge picture of 50 mg kg MN group.jpg]

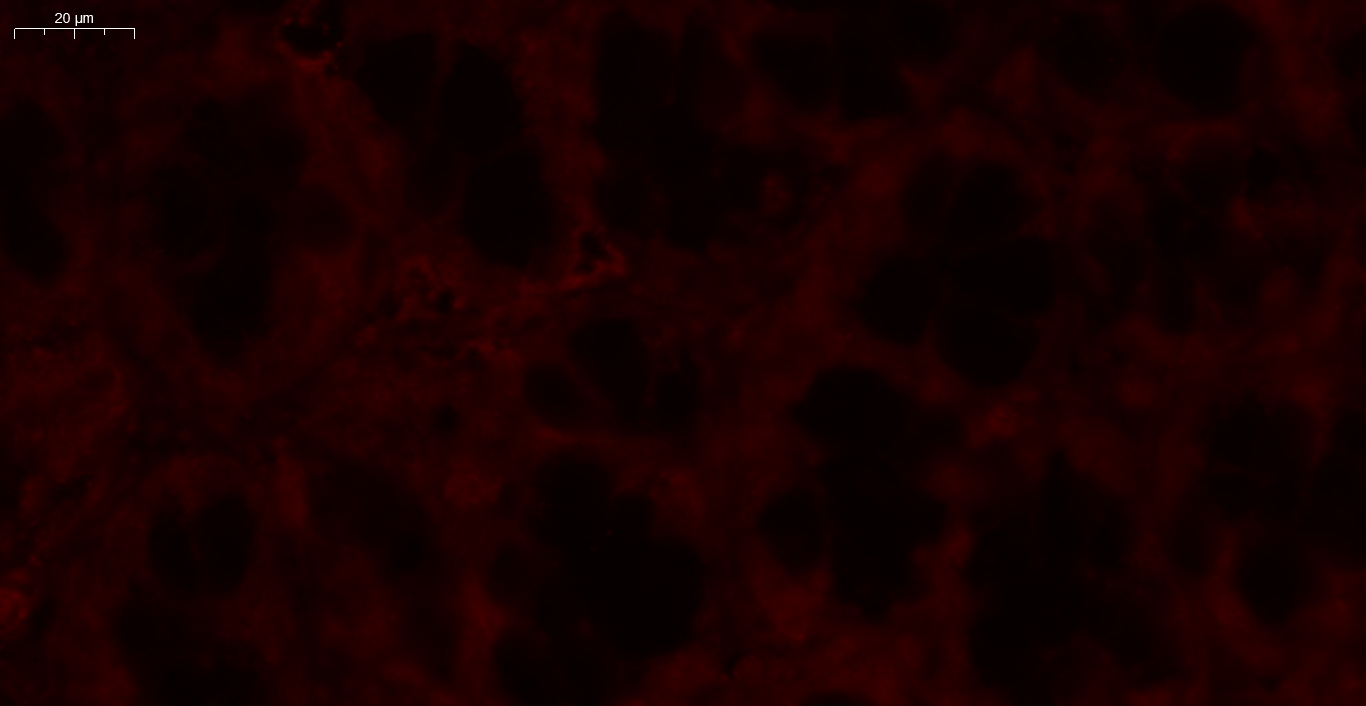

Supplement: Supplemental Information 8 — Immunofluorescence analysis of Nrf-2 [file peerj-10-14209-s008.zip › Fig. 8 raw data/Immunofluorescence analysis of Nrf-2/The group of 50mg kg MN/The Nrf-2 picture of 50 mg kg MN group.jpg]

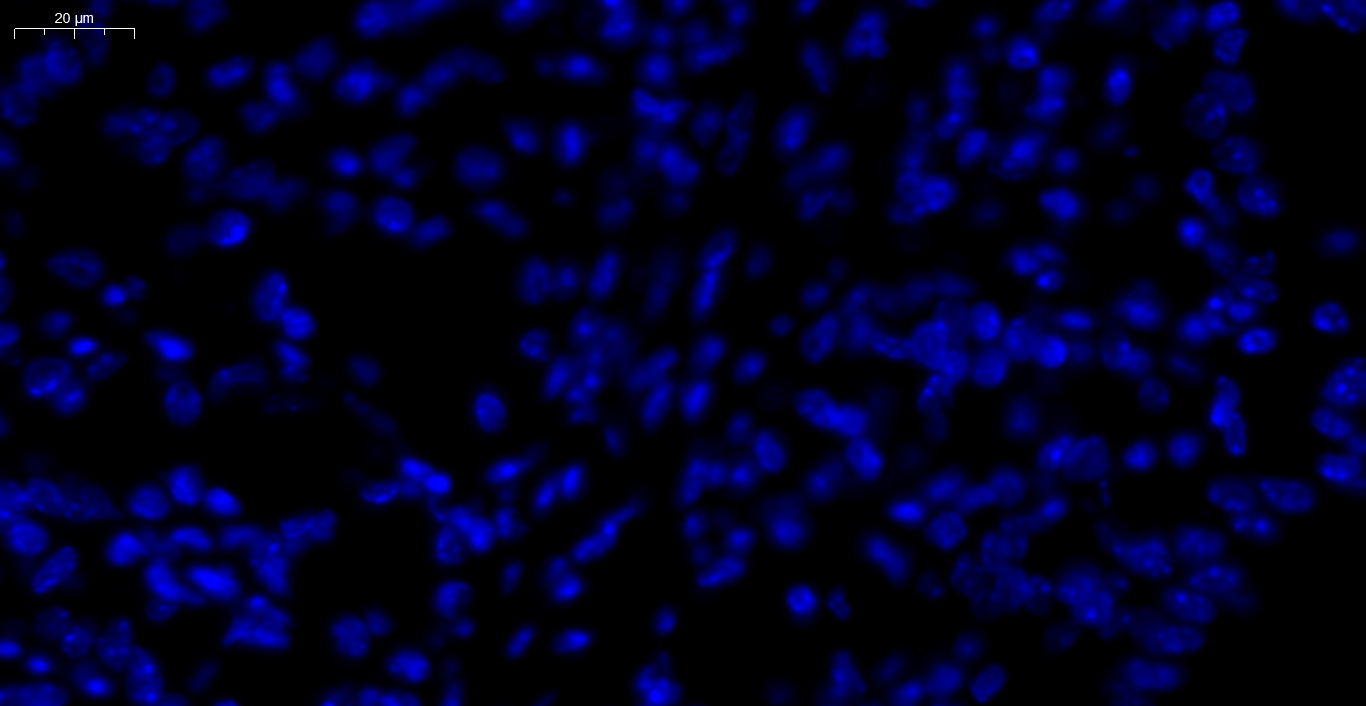

Supplement: Supplemental Information 8 — Immunofluorescence analysis of Nrf-2 [file peerj-10-14209-s008.zip › Fig. 8 raw data/Immunofluorescence analysis of Nrf-2/The group of DSS/The DAPI picture of DSS group.jpg]

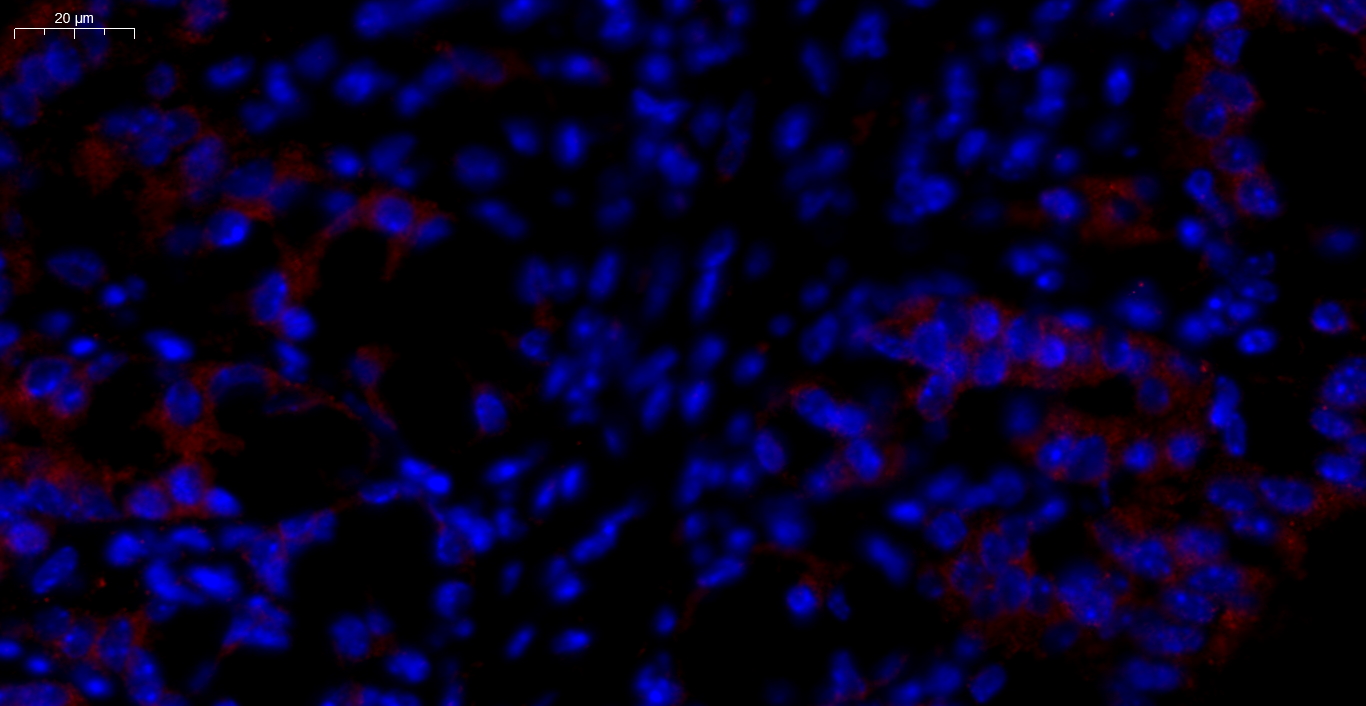

Supplement: Supplemental Information 8 — Immunofluorescence analysis of Nrf-2 [file peerj-10-14209-s008.zip › Fig. 8 raw data/Immunofluorescence analysis of Nrf-2/The group of DSS/The Merge picture of DSS group.jpg]

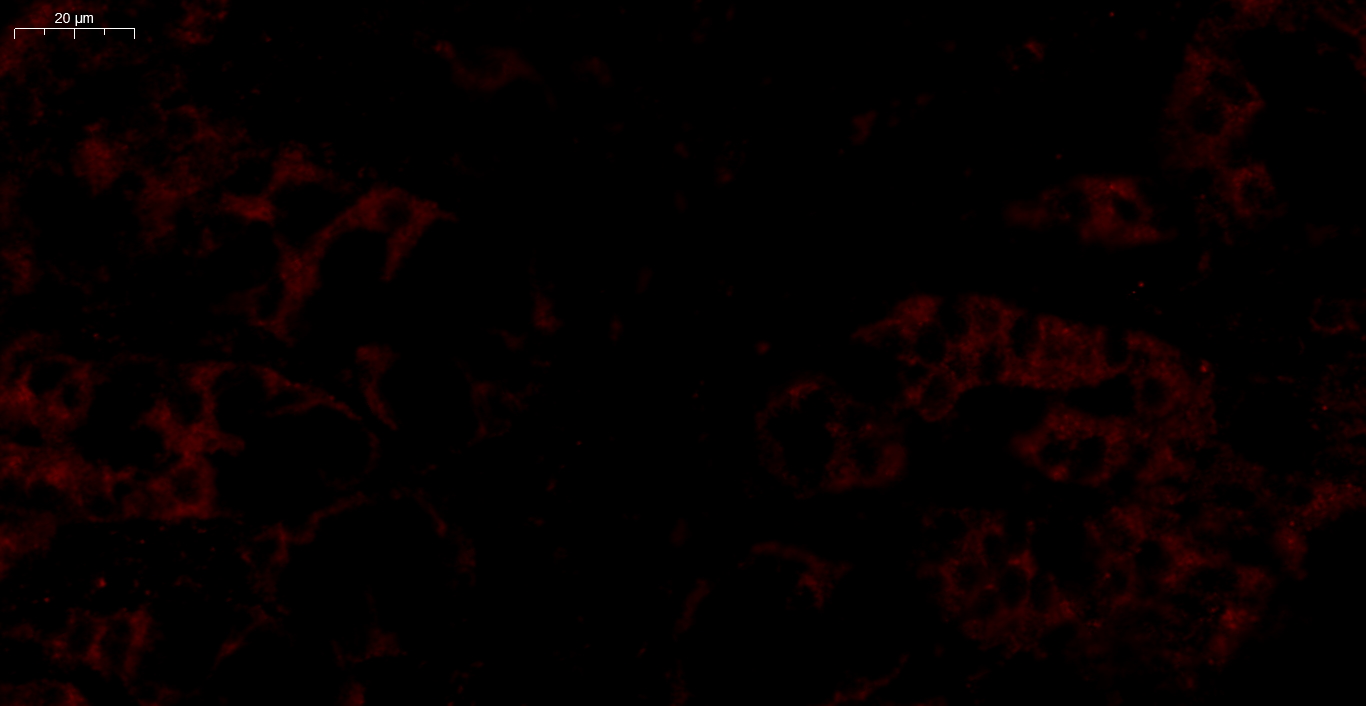

Supplement: Supplemental Information 8 — Immunofluorescence analysis of Nrf-2 [file peerj-10-14209-s008.zip › Fig. 8 raw data/Immunofluorescence analysis of Nrf-2/The group of DSS/The Nrf-2 picture of DSS group.jpg]

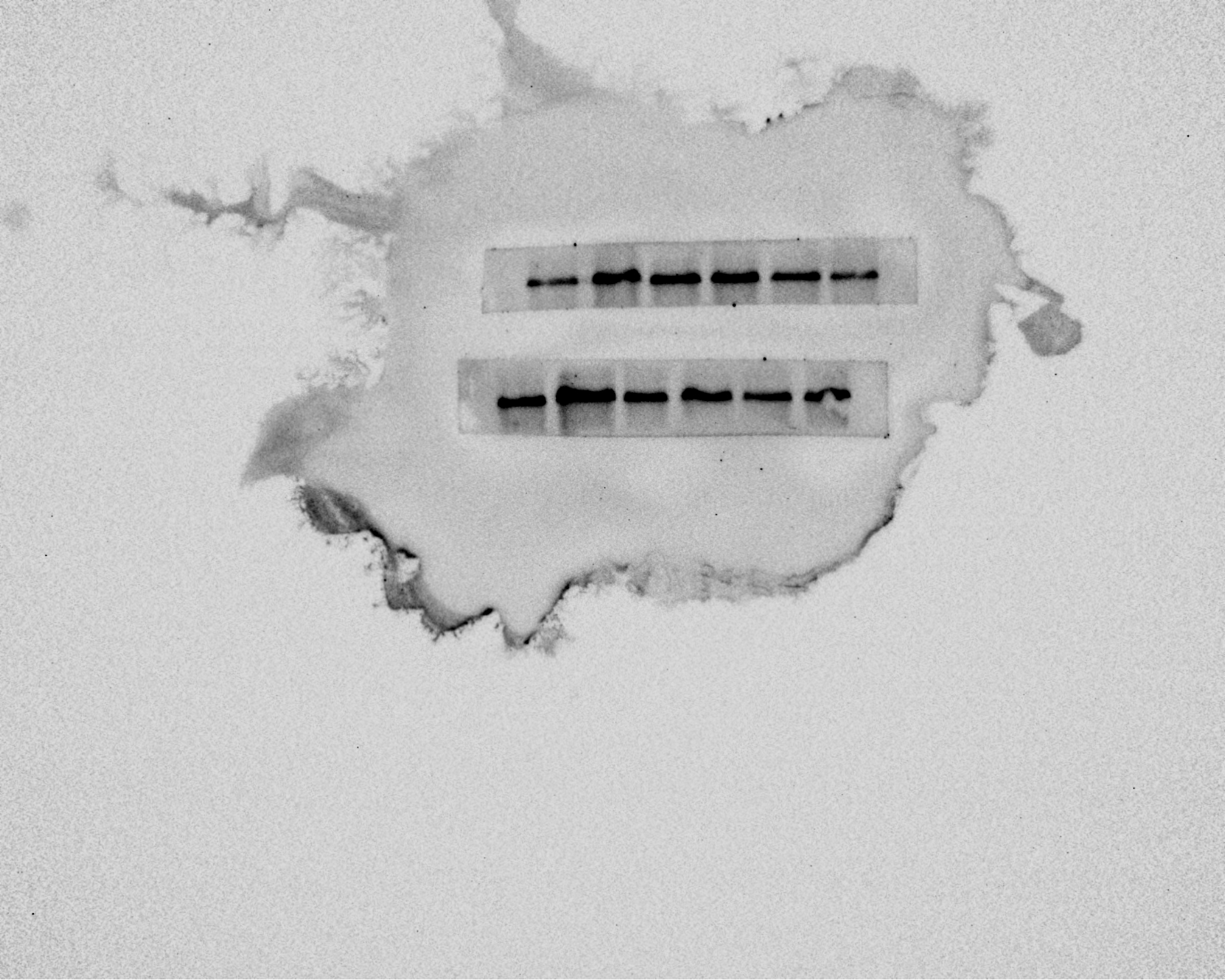

Supplement: Supplemental Information 9 [file peerj-10-14209-s009.zip › Fig. 9 raw data/Figure 9 original Western Blot images/A pJAK2.jpg]

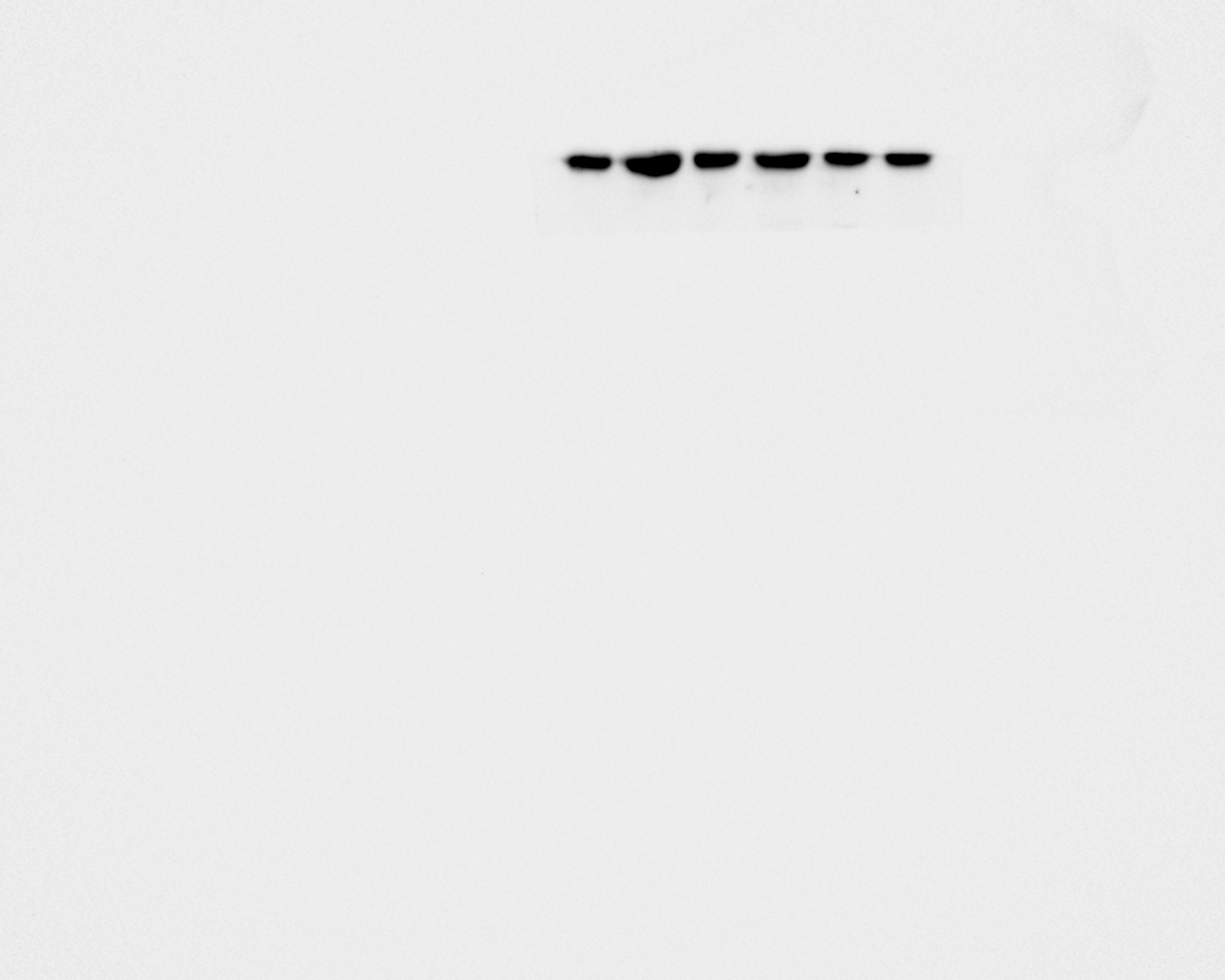

Supplement: Supplemental Information 9 [file peerj-10-14209-s009.zip › Fig. 9 raw data/Figure 9 original Western Blot images/A pSTAT3.jpg]

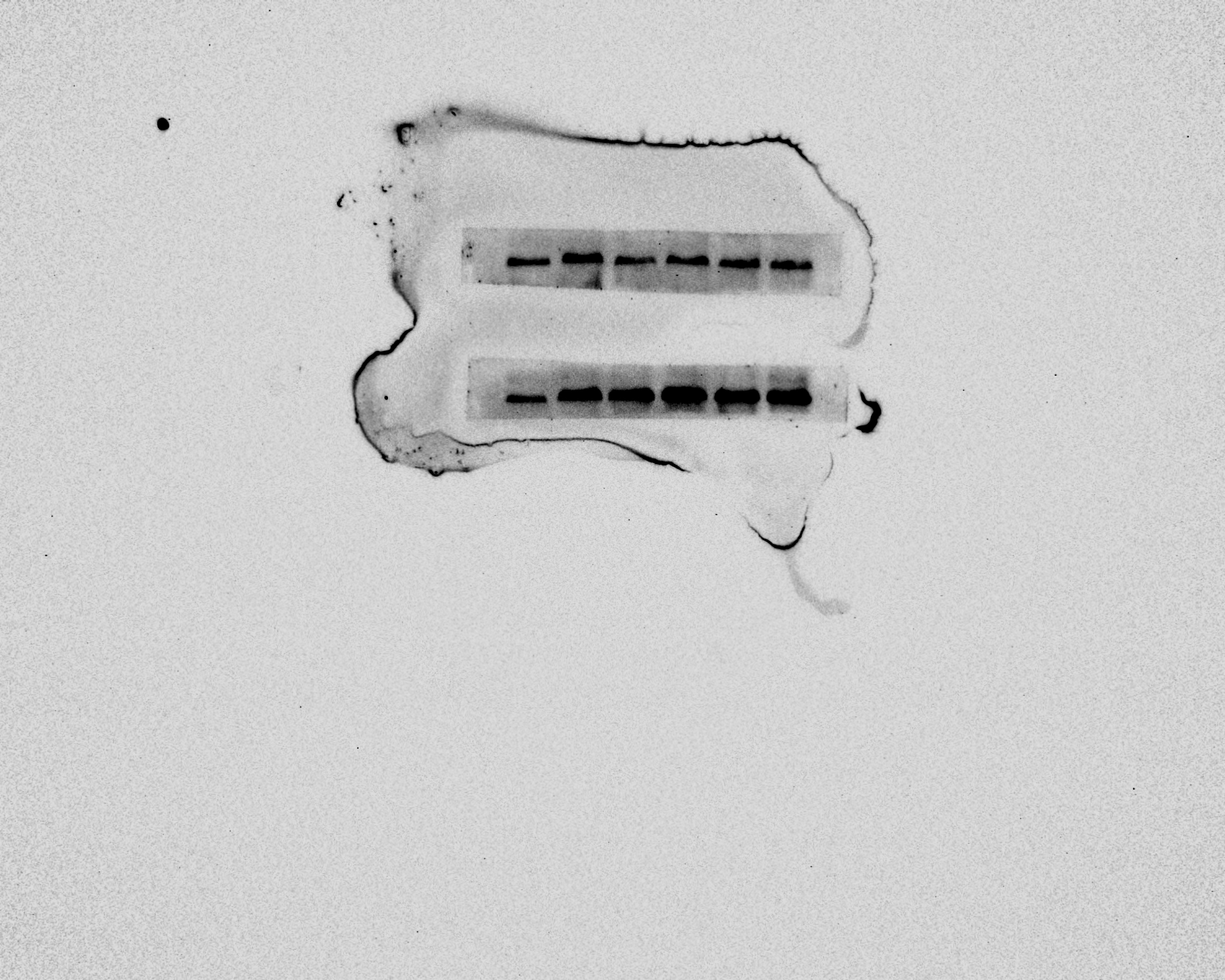

Supplement: Supplemental Information 9 [file peerj-10-14209-s009.zip › Fig. 9 raw data/Figure 9 original Western Blot images/B pJAK2 First Blot from the top down.jpg]

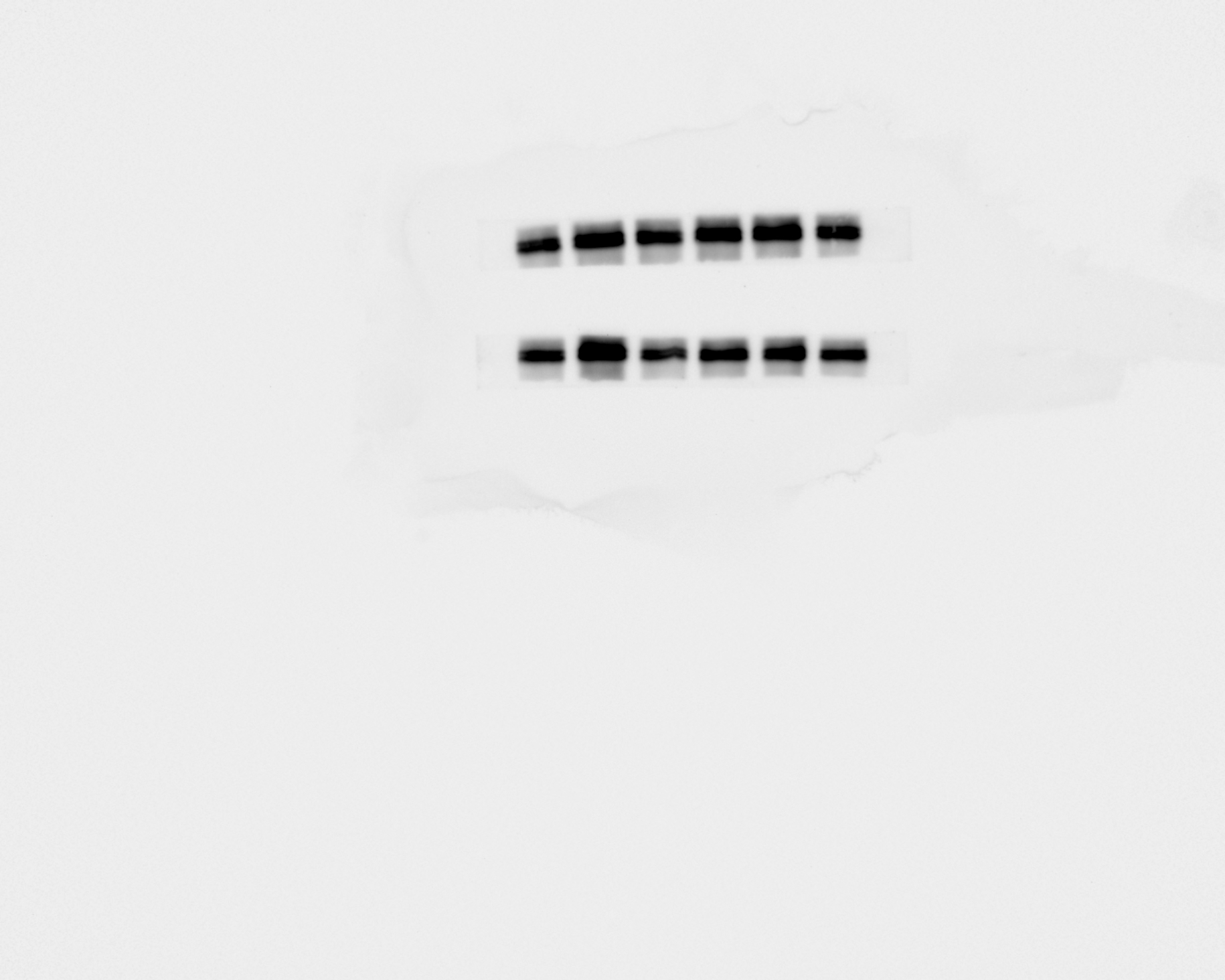

Supplement: Supplemental Information 9 [file peerj-10-14209-s009.zip › Fig. 9 raw data/Figure 9 original Western Blot images/B pSTAT3 Second Blot from the top down.jpg]

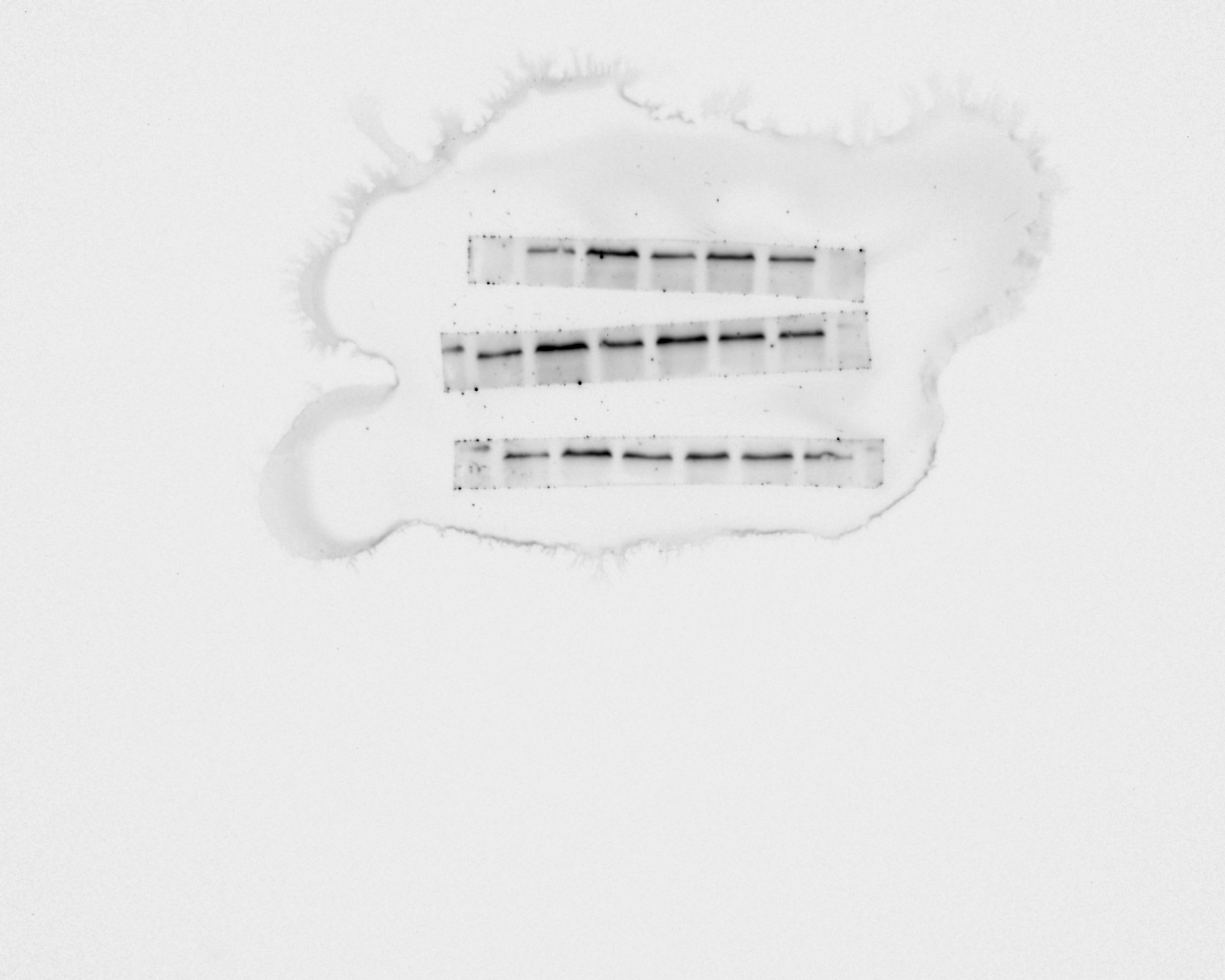

Supplement: Supplemental Information 10 [file peerj-10-14209-s010.zip › Fig. 10 raw data/Figure 10 original Western Blot images/A iNOS Second Blot from the top down.jpg]

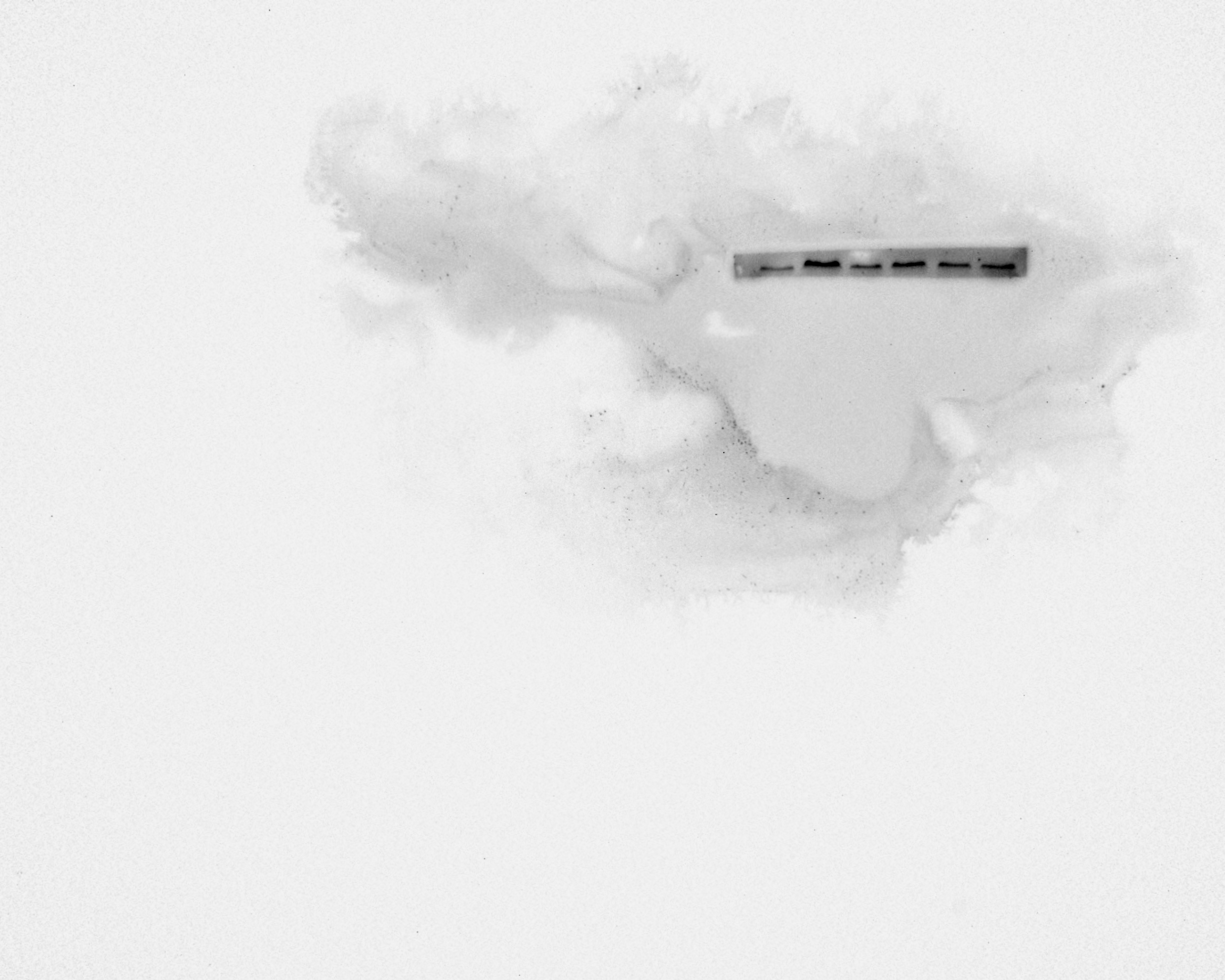

Supplement: Supplemental Information 10 [file peerj-10-14209-s010.zip › Fig. 10 raw data/Figure 10 original Western Blot images/B iNOS.tif]

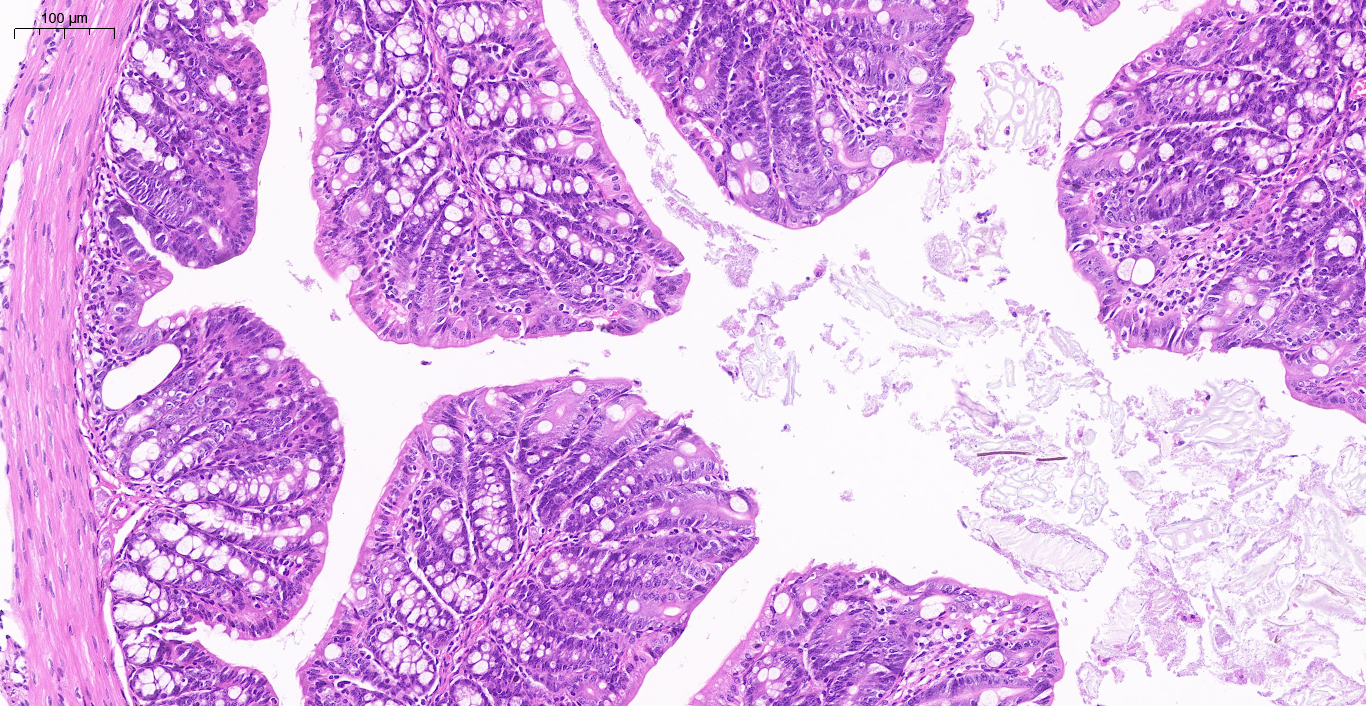

Supplement: Supplemental Information 11 — H&E data [file peerj-10-14209-s011.zip › Fig. 11 raw data/H&E data/100 mgkg MN.tif]

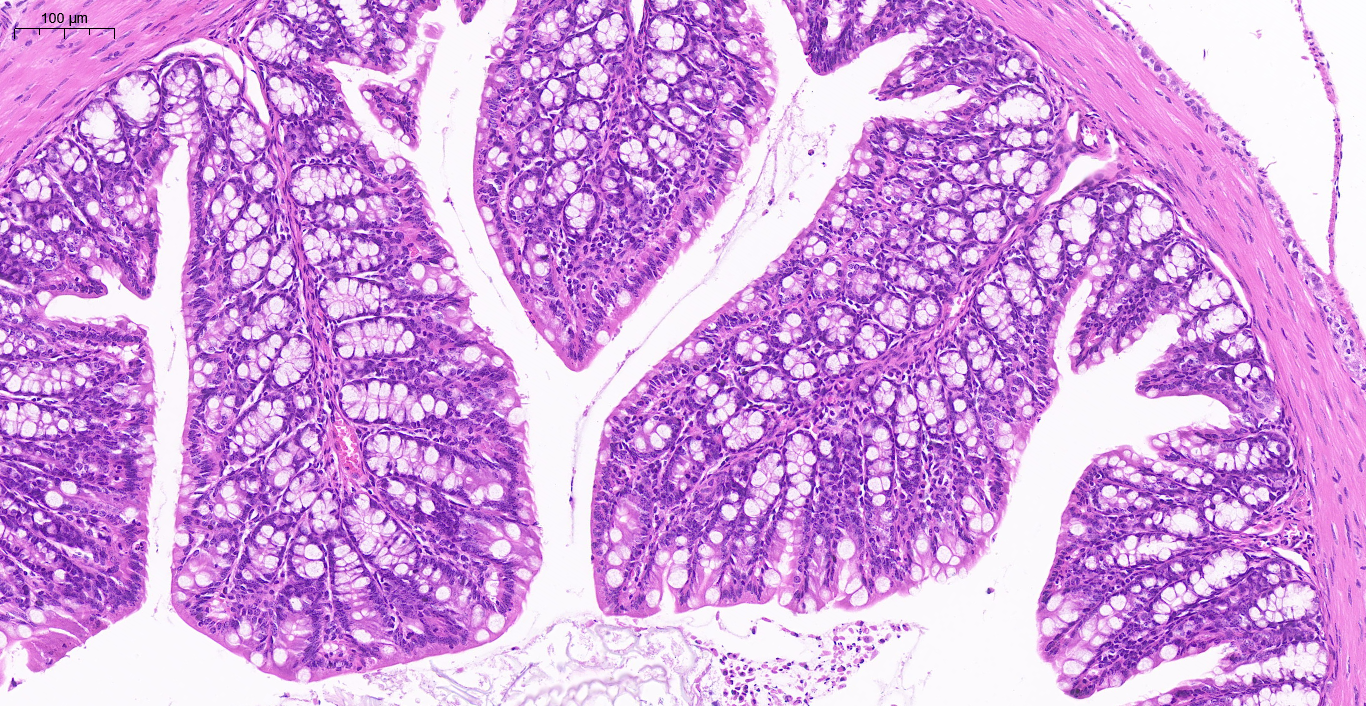

Supplement: Supplemental Information 11 — H&E data [file peerj-10-14209-s011.zip › Fig. 11 raw data/H&E data/200 mgkg MN.tif]

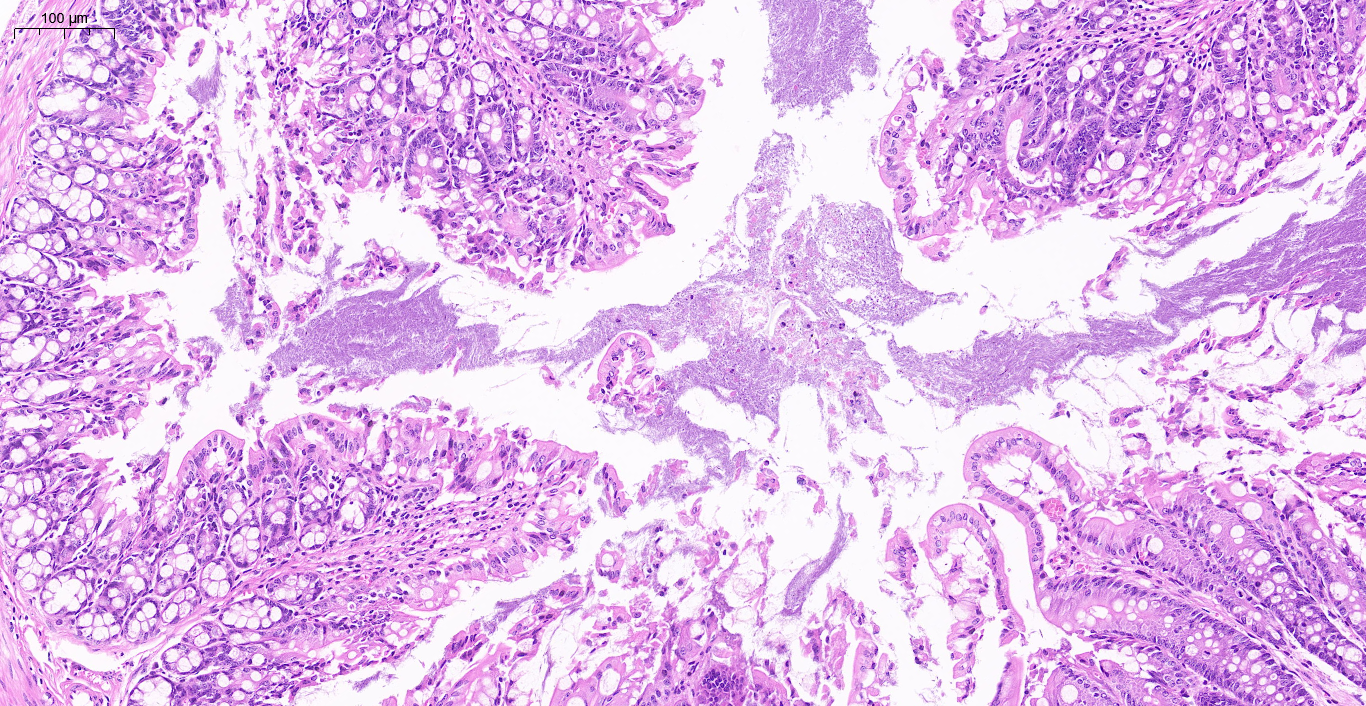

Supplement: Supplemental Information 11 — H&E data [file peerj-10-14209-s011.zip › Fig. 11 raw data/H&E data/50 mgkg MN.tif]

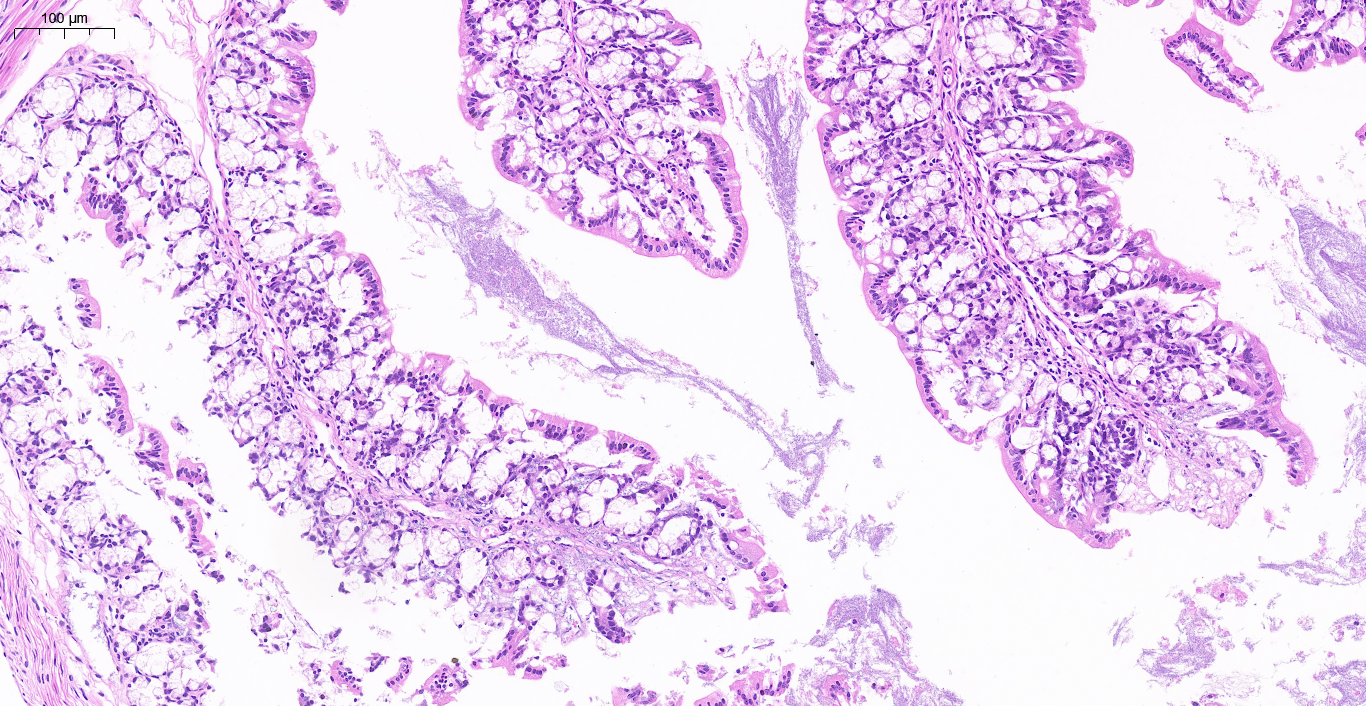

Supplement: Supplemental Information 11 — H&E data [file peerj-10-14209-s011.zip › Fig. 11 raw data/H&E data/500 mgkg 5-ASA.tif]

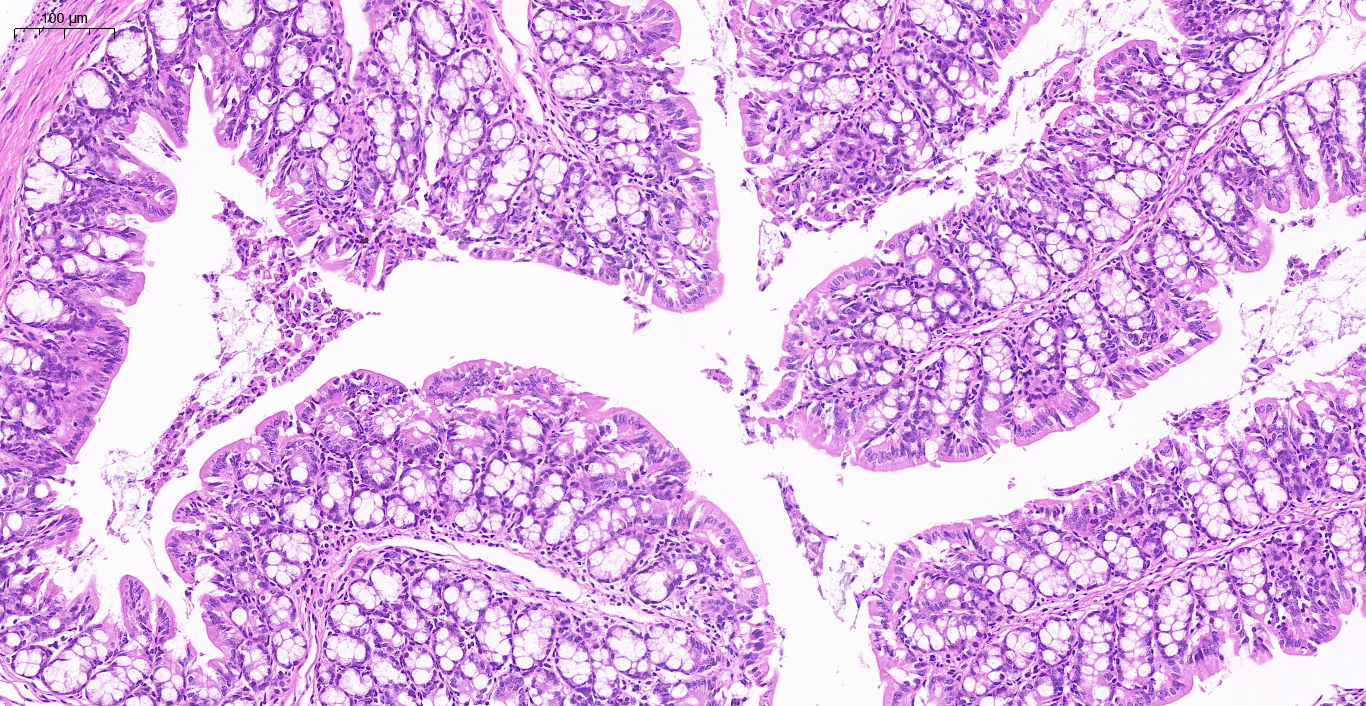

Supplement: Supplemental Information 11 — H&E data [file peerj-10-14209-s011.zip › Fig. 11 raw data/H&E data/Control.tif]

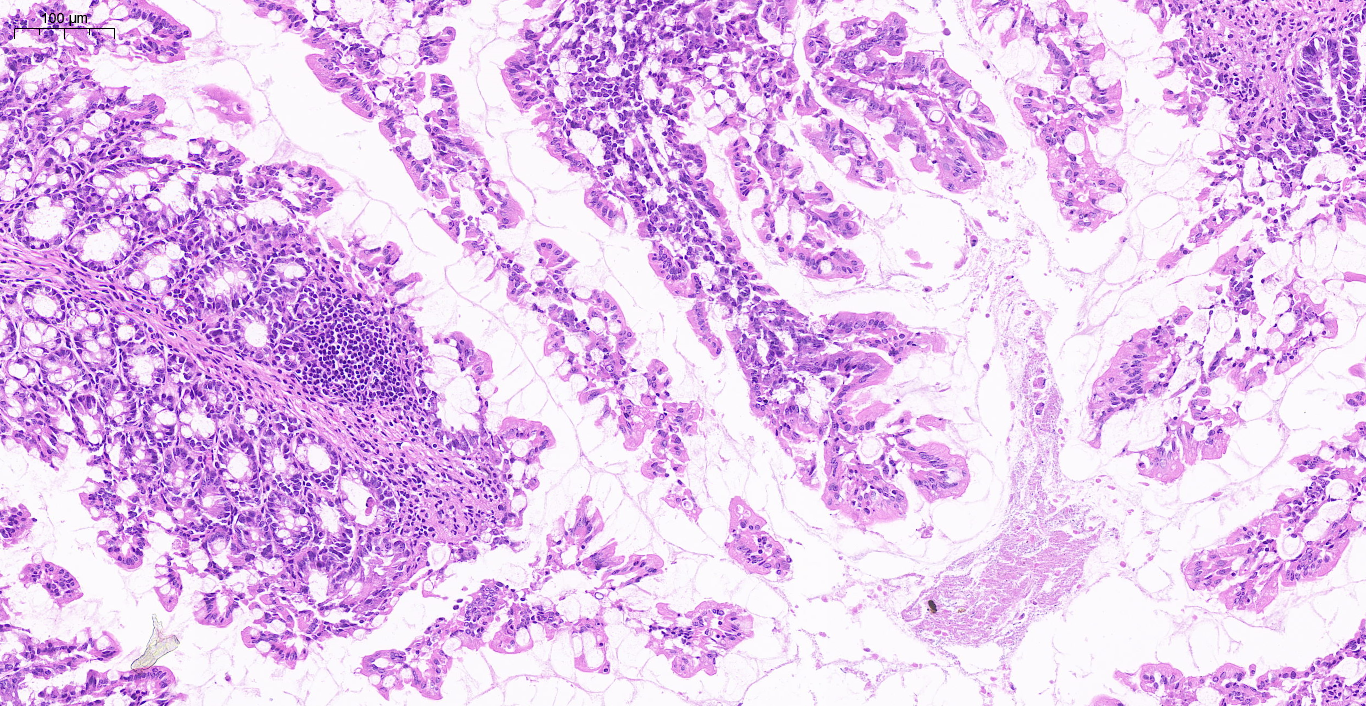

Supplement: Supplemental Information 11 — H&E data [file peerj-10-14209-s011.zip › Fig. 11 raw data/H&E data/DSS.tif]
