# Supplementary material for: Anti-inflammatory and anti-oxidant properties of Melianodiol on DSS-induced ulcerative colitis in mice
Source: PeerJ. 2022 Oct 25;10:e14209. doi: 10.7717/peerj.14209 (PMC9615967; doi:10.7717/peerj.14209)
Supplement: Supplemental Information 12 [file peerj-10-14209-s012.docx]

**A B**


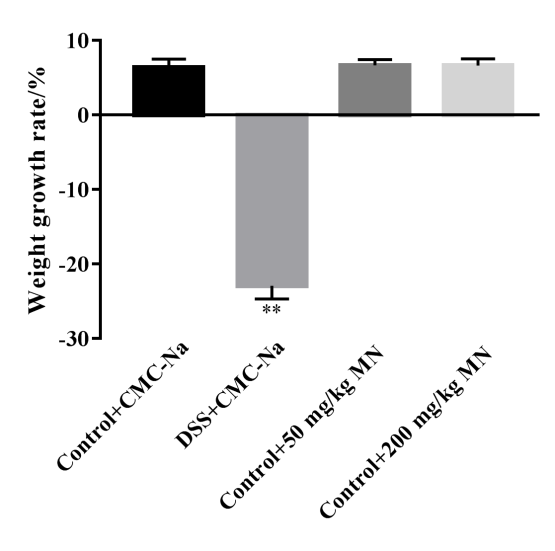

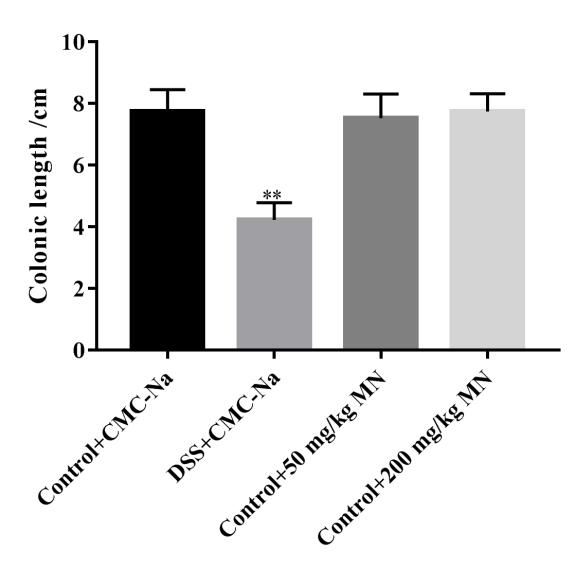


**FIGURE S1** Effect of MN (Melianodiol) on body weight change(%)**(A)** and colon length **(B)** in mice.


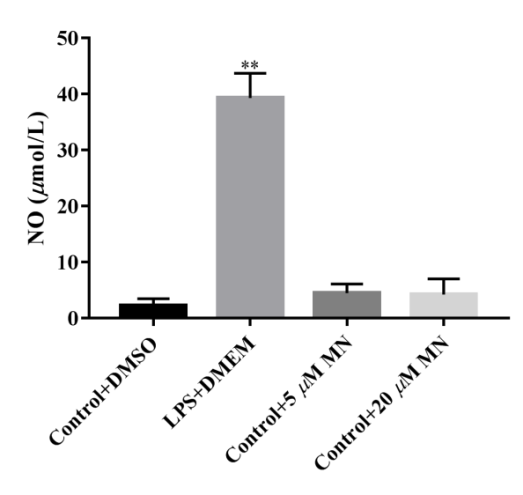


**FIGURE S2** Effect of MN on NO in RAW264.7 cells.
